# Rational design, synthesis, and evaluation of novel polypharmacological compounds targeting Na_V_1.5, K_V_1.5, and K_2_P channels for atrial fibrillation

**DOI:** 10.1016/j.jbc.2025.108387

**Published:** 2025-03-05

**Authors:** Lorena Camargo-Ayala, Mauricio Bedoya, Albert Dasí, Merten Prüser, Sven Schütte, Luis Prent-Peñaloza, Francisco Adasme-Carreño, Aytug K. Kiper, Susanne Rinné, Paola Andrea Camargo-Ayala, Paula A. Peña-Martínez, Alfonso Bueno-Orovio, Diego Varela, Felix Wiedmann, José C.E. Márquez Montesinos, Yuliet Mazola, Whitney Venturini, Rafael Zúñiga, Leandro Zúñiga, Constanze Schmidt, Blanca Rodriguez, Ursula Ravens, Niels Decher, Margarita Gutiérrez, Wendy González

**Affiliations:** 1 Doctorado en Ciencias Mención I + D de Productos Bioactivos, Instituto de Química de Recursos Naturales, Laboratorio de Síntesis Orgánica, Universidad de Talca, Talca, Chile; 2 Centro de Investigación de Estudios Avanzados del Maule (CIEAM), Vicerrectoría de Investigación y Postgrado, Universidad Católica del Maule, Talca, Chile; 3 Laboratorio de Bioinformática y Química Computacional (LBQC), Departamento de Medicina Traslacional, Facultad de Medicina, Universidad Católica del Maule, Talca, Chile; 4 Department of Computer Science, British Heart Foundation Centre of Research Excellence, University of Oxford, Oxford, UK; 5 Department of Cardiology, University of Heidelberg, Heidelberg, Germany; 6 DZHK (German Center for Cardiovascular Research), partner site Heidelberg /Mannheim, University of Heidelberg, Heidelberg, Germany; 7 HCR, Heidelberg Center for Heart Rhythm Disorders, University of Heidelberg, Heidelberg, Germany; 8 Institute for Physiology and Pathophysiology, Philipps-University Marburg, Marburg, Germany; 9 Departamento de Ciencias Químicas, Facultad de Ciencias Exactas, Universidad Andrés Bello, Viña del Mar, Chile; 10 Institute of Physiology, University Medicine Greifswald, Greifswald, Germany; 11 Doctorado en Ciencias Biomédicas, Laboratorio de Patología Molecular, Departamento de Ciencias Básicas Biomédicas, Facultad de Ciencias de la Salud, Universidad de Talca, Talca, Chile; 12 Doctorado en Ciencias Agrarias, Facultad de Ciencias Agrarias, Universidad de Talca, Talca, Chile; 13 Laboratorio de Química Enológica, Facultad de Ciencias Agrarias, Universidad de Talca, Talca, Chile; 14 Millennium Nucleus of Ion Channels-Associated Diseases (MiNICAD), Santiago, Chile; 15 Program of Physiology and Biophysics, Institute of Biomedical Sciences, Faculty of Medicine, Universidad de Chile, Santiago, Chile; 16 Centro de Bioinformática, Simulación y Modelado (CBSM), Universidad de Talca, Talca, Chile; 17 Centro de Nanomedicina, Diagnóstico y Desarrollo de Fármacos (ND3), Laboratorio de Fisiología Molecular, Escuela de Medicina, Universidad de Talca, Talca, Chile; 18 Departamento de Medicina Traslacional, Facultad de Medicina, Universidad Católica del Maule, Talca, Chile; 19 German Atrial Fibrillation Competence NETwork (AFNET), Freiburg, Germany; 20 Institute of Experimental Cardiovascular Medicine, University Heart Center Freiburg – Bad Krozingen, Medical Center – University of Freiburg and Faculty of Medicine, Freiburg, Germany; 21 Laboratorio Síntesis Orgánica y Actividad Biológica (LSO-Act-Bio), Instituto de Química de Recursos Naturales, Universidad de Talca, Talca, Chile

**Keywords:** atrial fibrillation, ion channels, K_2_P channels, K_V_1.5, Na_V_1.5, Polypharmacology, multi-target inhibitors

## Abstract

Atrial fibrillation (AF) involves electrical remodeling of the atria, with ion channels such as Na_V_1.5, K_V_1.5, and TASK-1 playing crucial roles. This study investigates acetamide-based compounds designed as multi-target inhibitors of these ion channels to address AF. Compound **6f** emerged as the most potent in the series, demonstrating a strong inhibition of TASK-1 (IC_50_ ∼ 0.3 μM), a moderate inhibition of Na_V_1.5 (IC_50_ ∼ 21.2 μM) and a subtle inhibition of K_V_1.5 (IC_50_ ∼ 81.5 μM), alongside unexpected activation of TASK-4 (∼ 40% at 100 μM). Functional assays on human atrial cardiomyocytes from sinus rhythm (SR) and patients with AF revealed that **6f** reduced action potential amplitude in SR (indicating Na_V_1.5 block), while in AF it increased action potential duration (APD), reflecting high affinity for TASK-1. Additionally, **6f** caused hyperpolarization of the resting membrane potential in AF cardiomyocytes, consistent with the observed TASK-4 activation. Mathematical modeling further validated its efficacy in reducing AF burden. Pharmacokinetic analyses suggest favorable absorption and low toxicity. These findings identify **6f** as a promising multi-target therapeutic candidate for AF management.

The regular beating of the heart depends on the generation of action potentials (AP) that trigger coordinated contractions. Each AP is governed by the activity of ion channels in cardiac cells. Alterations in these processes can lead to arrhythmias such as atrial fibrillation (AF), the most common arrhythmia globally, associated with significant cardiovascular morbidity and mortality (1, 2), impacting millions of individuals annually (3, 4).

Electrical remodeling of the atria, a hallmark of AF (5, 6, 7), is characterized by changes in ion channel expression and function, which alter action potential duration (APD) and atrial refractoriness (6). To counter these changes, atrial ion channels are key therapeutic targets.

Voltage-dependent potassium channels K_V_1.5, preferentially expressed in atria but not in ventricles (7, 8), mediate the ultrafast potassium outflow current (*I*_Kur_) and regulate cellular repolarization, shaping the APD (9). K_V_1.5 channel blockers selectively prolong atrial refractoriness without inducing ventricular proarrhythmic effects (8), making K_V_1.5 a promising pharmacological target for the treatment of AF (10, 11, 12, 13). Consequently, several *I*_Kur_ blockers have been developed (8, 14, 15, 16).

Other potassium channels contributing to the atrial AP include the two-pore domain (K_2P_) channel TASK-1 (17), which is also predominantly expressed in the atria (17, 18, 19). In AF, increased TASK-1 expression accelerates repolarization and shortens AP (19, 20, 21). Additionally, the current density of TASK-1 in human atrial cardiomyocytes isolated from patients with AF is three times higher than in patients with sinus rhythm (18). Blocking TASK-1 normalizes AP-duration, making it an attractive antiarrhythmic target (12, 17, 18, 19, 21, 22, 23, 24). Moreover, it has been reported that K_V_1.5 blockers used against AF can also inhibit TASK-1 (25), suggesting that a polypharmacological strategy targeting both K_V_1.5 and TASK-1 could enhance clinical efficacy.

Another channel that plays an essential role in the heart is the sodium channel Na_V_1.5 (25, 26), responsible for the initial depolarizing phase of the AP (27). An increased sodium current (I_Na_) mediated by Na_V_1.5 has been linked to increased atrial excitability and AF (28). Although Na_V_1.5 is present in both atria and ventricles, its differential atrial properties make it an attractive pharmacological target for AF treatment (7, 9, 29). In fact, class I antiarrhythmic drugs, targeting Na_V_1.5 channel, have long been used for rhythm control in clinical practice, highlighting the relevance of Na_V_1.5 inhibition in AF therapy (30).

The development of effective and safe antiarrhythmic drugs remains an unmet need (31), as available options are limited by poor efficacy and adverse effects (12, 19). Moreover, pure *I*_Kur_ channel blockade may not be sufficient to suppress AF (8). Therefore, it has been proposed that multichannel blockers could represent better strategies for treating AF (31). For example, blockers targeting K_V_1.5 and Na_V_1.5 channels can generate a synergistic anti-AF effect, without inducing ventricular proarrhythmic effects (31, 32, 33). Similarly, the inhibition of I_Na_ and multi K^+^-currents synergistically normalizes the shortened AP in AF patients (32).

Local anesthetic (LA), such as lidocaine, ropivacaine, and bupivacaine (Fig. 1), are clinically used antiarrhythmics (34, 35) with multiple mechanisms of action (35). Ropivacaine and bupivacaine block Na_V_1.5, TASK-1 and K_V_1.5 channels, while lidocaine blocks Na_V_1.5 and TASK-1 (36, 37, 38, 39, 40, 41). This study aims to design and synthesize new molecules based on the shared chemical features of LA to inhibit atrial ion channels Na_V_1.5, TASK-1, and K_V_1.5.

**Figure 1:**
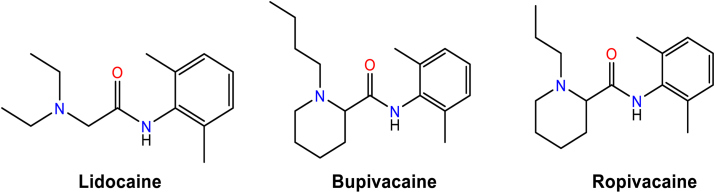
**Structures of LA compounds that block K_V_1.5, TASK-1 and/or Na_V_1.5 channels.** From the *right* to the *left* of the compounds are shown the aromatic ring, an intermediate amide linker, and a hydrophilic domain.

## Results

### *In silico* design: molecular docking

#### Ligand design

**Figure 2:**
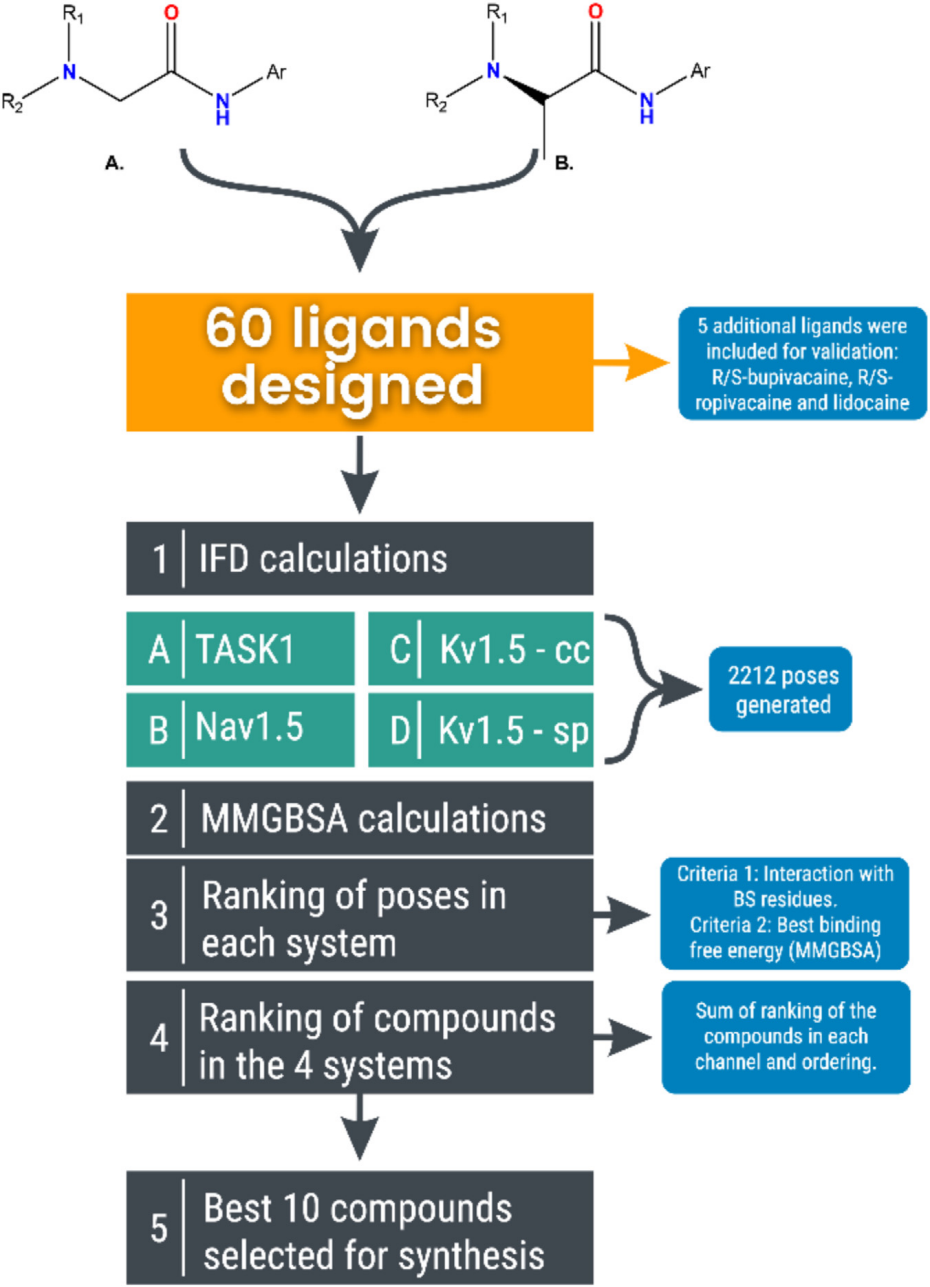
**General scheme of design and selection of compounds for synthesis**. *A*, Derivatives of bromoacetic acid. *B*, (*S*)-2-bromo-propionic acid derivatives. Ar, *aromatic amines*. R1 and R2, *aliphatic amines*.

The pharmacophore of LA includes key features crucial for their activity: a hydrophobic domain or aromatic ring (mono, di, or tri-substituted), an intermediate amide linker, a hydrophilic domain (amine) (Fig. 1), and proton donor and acceptor groups interacting with amino acid residues at the binding site (BS). Based on three criteria: (a) the common LA pharmacophore of bupivacaine, ropivacaine, and lidocaine (b) their binding modes in K_V_1.5, TASK-1, and Na_V_1.5 channels and (c) synthesis viability, we proposed a basic core for the new molecules (Fig. 2, *A* and *B*).

Aromatic amines (Ar) were selected to explore various substituents. Aliphatic amines (R_1_ and R_2_) were chosen for variability in chain extension and cyclic or acyclic structures. Two series of compounds were proposed, 30 derived from bromoacetic acid (Fig. 2*A*) and 30 from (*S*)-2-bromo-propionic acid (Fig. 2*B*), totaling 60 compounds.

#### Computational analysis

Induced-fit docking (IFD) calculations were performed for the 60 candidates with each channel to analyze the most probable binding mode and ligand-receptor interactions, using BS references for LA-type blockers: lidocaine at Na_V_1.5 (42), bupivacaine enantiomers in TASK-1 (43), and bupivacaine/ropivacaine in K_V_1.5 (15). IFD generated up to 10 poses *per* ligand.

For the molecular docking calculations in TASK-1, a single protein lateral fenestration was explored due to the symmetrical nature of both subunits. The exploration of fenestration considered the BS residues reported for bupivacaine. Similarly, in the case of K_V_1.5, the residues reported as key in the binding of the LA bupivacaine and ropivacaine were considered. However, since interactions with LA have been documented in both the central cavity (CC) and side pockets (SP) of K_V_1.5, separate molecular docking calculations were performed for each of these sites. For Na_V_1.5, analysis focused on residues associated with lidocaine binding in domains III and IV.

Approximately 550 poses were obtained *per* system, totaling 2212 poses (Step 1, Fig. 2), with 10 poses *per* ligand for 60 designed compounds and five validation compounds (*R*/*S* bupivacaine, *R*/*S* ropivacaine and lidocaine) across TASK-1, Na_V_1.5, and K_V_1.5 (CC and SP) channels.

Once molecular docking results were acquired, binding free energy calculations were performed using the MM-GBSA method to re-score and analyze the poses obtained for each channel (Step 2, Fig. 2).

#### Selection of the best ligands: Total score and location in the ranking

Two criteria were integrated to rank the compounds and identify those with the highest polypharmacological potential, namely, the highest activity across all three channels. The first criterion encompassed the interaction of the tested compounds with residues within the documented BS of LA in each channel, based on the inclusion of the LA pharmacophore in the molecule design. The second criterion involved binding free energy determination using the MM-GBSA method, assuming free energy could predict the potential affinities of the studied compounds.

In summary, we devised a methodology for ranking the compounds by identifying those with the highest number of interactions and optimal binding free energy values in each channel. This phase referred to as "ranking of poses," is illustrated in Step 3, Figure 2.

Since multiple poses were obtained from docking results, in this step was selected the best pose satisfying the two aforementioned criteria for each compound. Binding free energy values and a maximum number of interactions were normalized and used to arrange the poses, ensuring the most favorable pose received the highest cumulative value. Subsequently, a "ranking of compounds" was executed *per* channel (Table 1).

**Table 1:** Ranking of the 25 compounds with the highest polypharmacological potential on TASK1, K_V_1.5 and Na_V_1.5 channels

Position	Name
1	**6d**
2	**6f**
3	**7d**
4	**6a**
5	**7c**
6	**7a**
7	**6c**
8	**ropivacaine *S***
9	**7b**
10	**bupivacaine *R***
11	**6e**
12	**6b**
13	**6p**
14	**6x**
15	**7i**
16	**7w**
17	**7aa**
18	**7h**
19	**bupivacaine *S***
20	**7x**
21	**7j**
22	**ropivacaine *R***
23	**6z**
24	**7ab**
25	**6o**

For K_V_1.5, it is known that the *R/S* bupivacaine mixture has an IC_50_ of 31 μM, while *S*-ropivacaine has an IC_50_ of 128.9 μM in oocytes (15). This aligns with the K_V_1.5 ranking (Table S1), where *R/S* bupivacaine ranks second and third, and *S*-ropivacaine ranks fifth in the CC.

However, for K_V_1.5 side-pockets (Table S2), *S*-ropivacaine ranks 12th, and *R*/S-bupivacaine ranks 18th and 20th. The effect in K_V_1.5 is complex as the LA can bind in both sites (CC and SP). However, IC_50_ values align with optimal *R/S*-bupivacaine positions of in the CC.

The IC_50_ values for *R*/*S*-bupivacaine, *R*/*S*-ropivacaine, and lidocaine in TASK-1 are 12.1 μM, 17.6 μM, and 53.8 μM, respectively (38). In the TASK-1 ranking, *R/S*-ropivacaine ranked 19th and 21st, *R/S*-bupivacaine 33rd and 62nd, and lidocaine 57th. This could align with the narrow range of IC_50_ values, with a subtle trend favoring ropivacaine and *R*-bupivacaine. It is important to note, however, that many designed compounds outperform these references (Table S3).

For Na_V_1.5 in oocytes, the *R*/*S* buvipacaine mixture has a lower IC_50_ value (4.5 μM (39)) compared to lidocaine (57.9 μM). This aligns with the Na_V_1.5 ranking (Table S4), where *R/S* bupivacaine ranks sixth and ninth, while lidocaine ranks 52nd.

After analyzing the rankings separately, the ranking for each compound in each channel was compared to identify the compounds with the lowest total ranking values (Step 4, Fig. 2). Table 1 lists the top 25 compounds with the highest polypharmacological potential, while Table S5 includes all 65 compounds. The top 10 compounds (excluding references) were selected for synthesis and electrophysiology experiments across the three channels.

### Chemistry

The synthesis involved two steps: (1) Amide bond formation, and (2) Nucleophilic substitution. In the first step (Fig. 3), amides were synthesized using a DIC coupling reagent, achieving 96%–98% yield in 1 h. After purification and drying, bromine in the amide was substituted with a secondary amine in a reaction lasting 24 h. Ultimately, the compounds underwent purification through column chromatography. The compounds were obtained with yields ranging from moderate to good (55%–84%), and displayed high purity levels (Figs. 4 and S5–S61).

**Figure 3:**
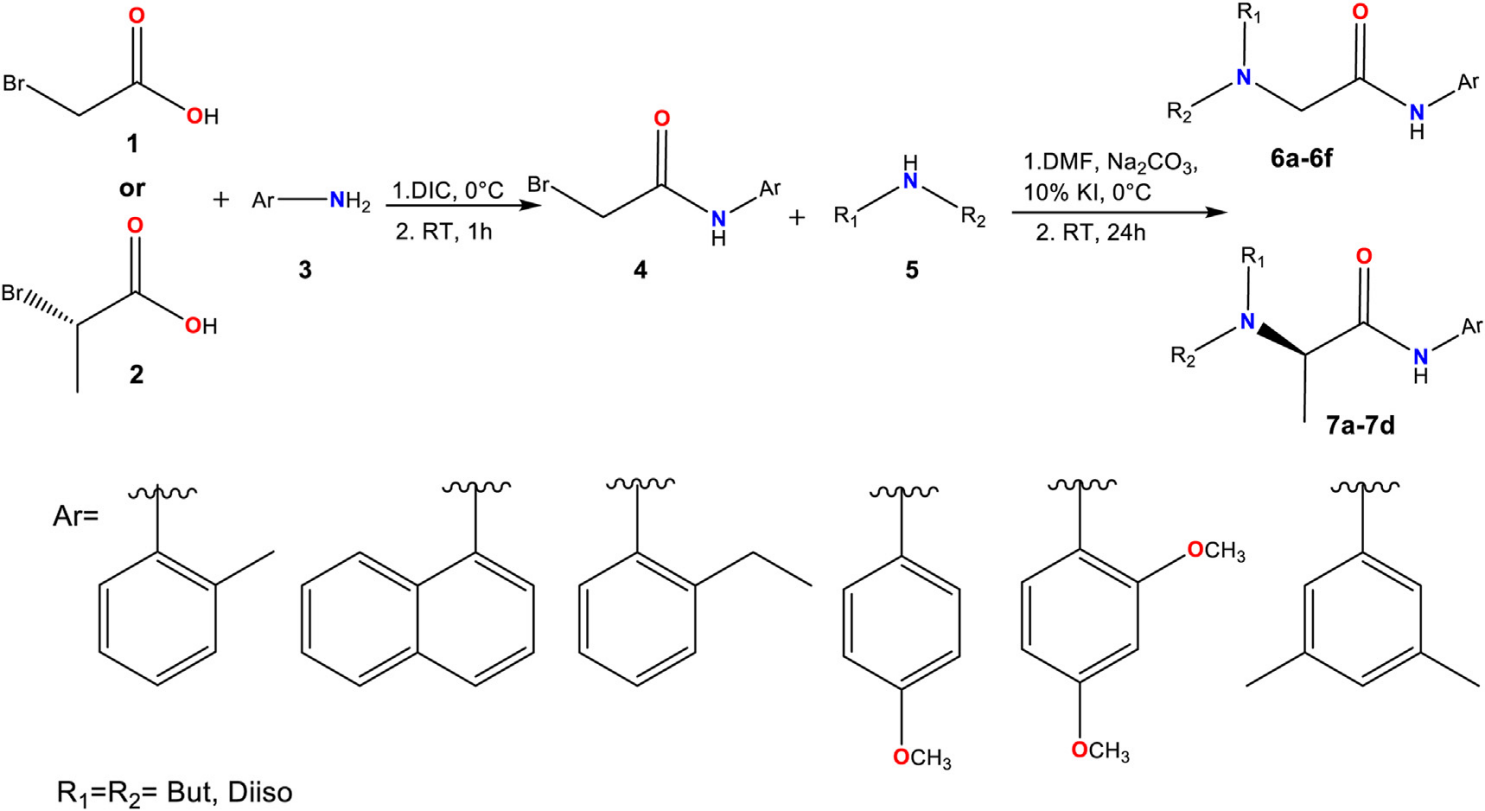
**Synthesis of the set of compounds 6 and 7**.

**Figure 4:**
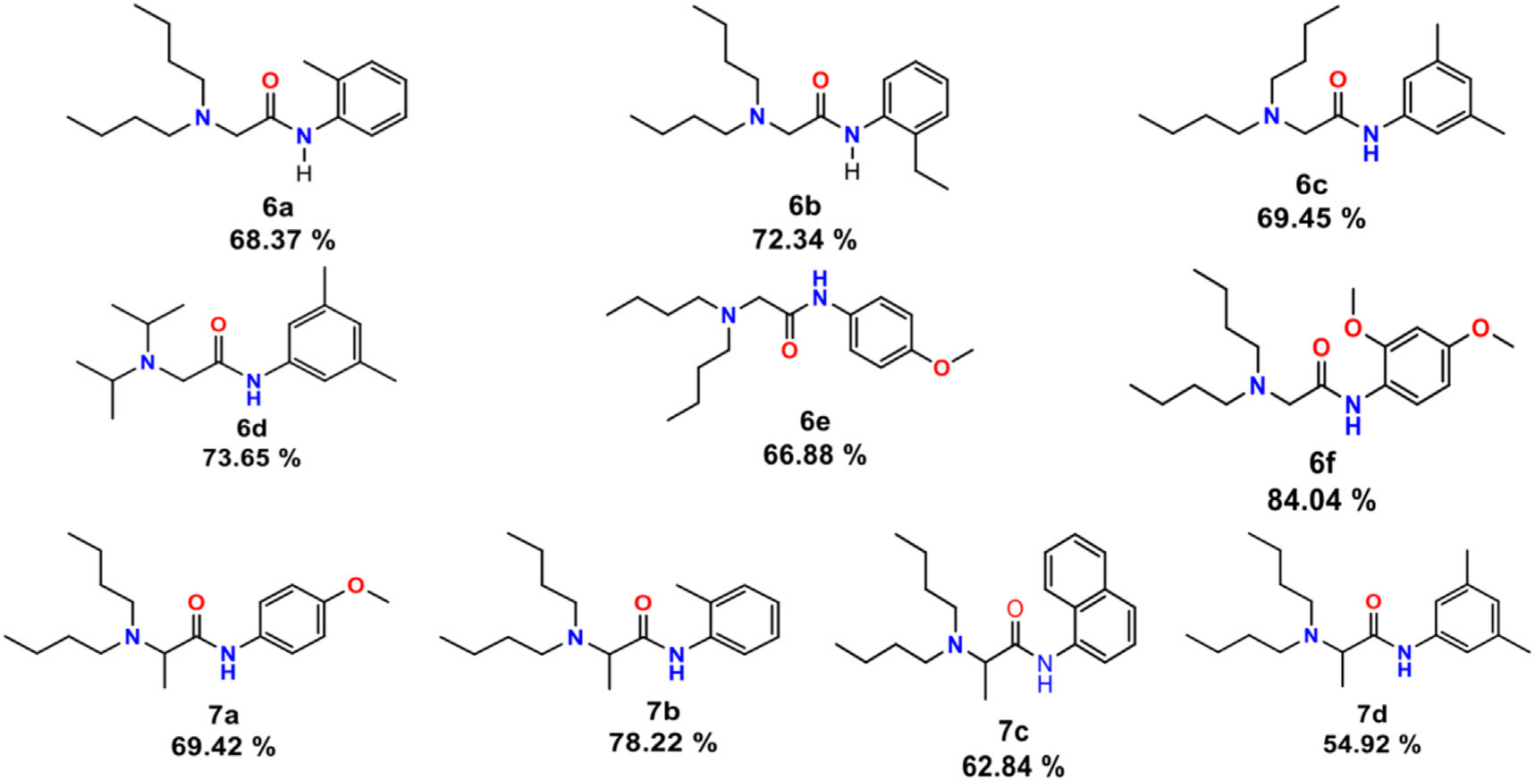
**Structure of compounds 6a-6f and 7a-7d and their yield**.

### Biological assay

#### Preliminary testing

The two-electrode voltage clamp (TEVC) technique was used for electrophysiology tests. Channels were expressed in *Xenopus laevis* oocytes. The initial channel-blocking potential was assessed at 100 μM for the 10 synthesized compounds on TASK-1 and K_V_1.5 channels.

**Figure 5:**
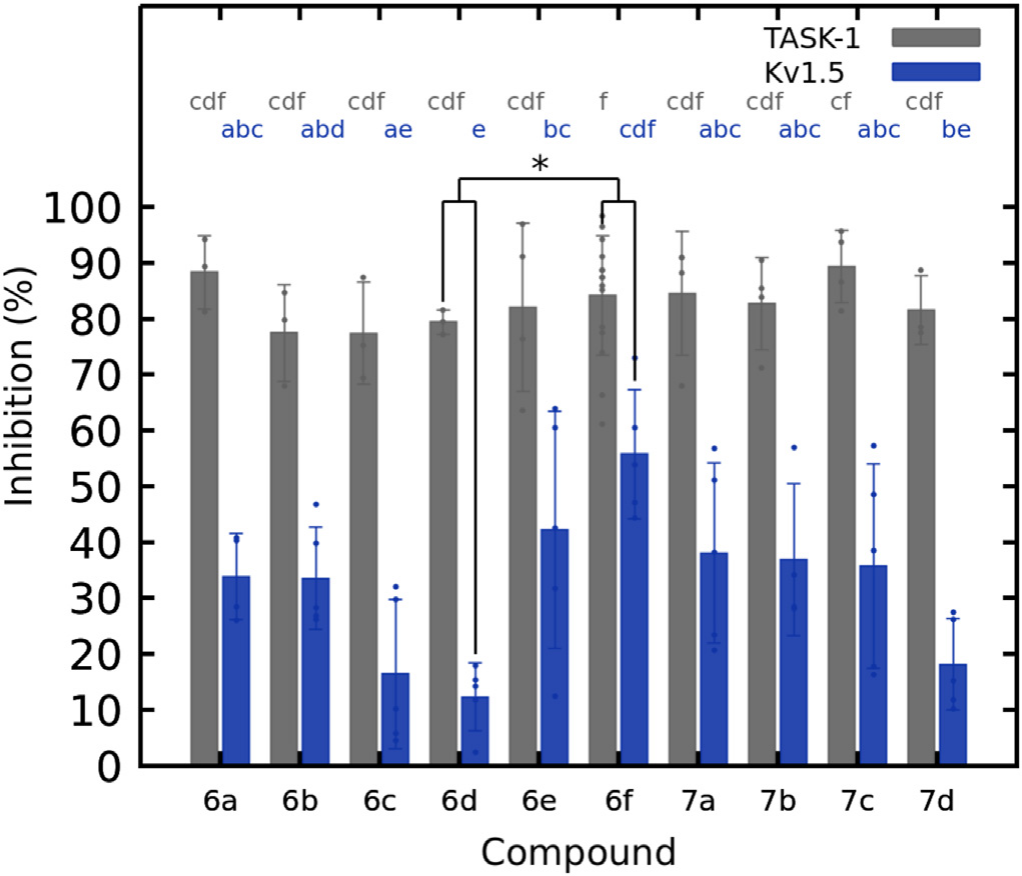
**The inhibitory activity of compounds 6a-6f and 7a-7d were evaluated in TASK-1 and K_V_1.5 channels using the TEVC technique at +40 mV and a concentration of 100 μM**. For TASK-1; n = 4 (**6e, 7a, 7b, 7c**), n = 3 (**6a, 6b, 6c, 6d, 7d**), n = 15 for **6f** and for K_V_1.5; n = 5 (except in **6a** and **7b**, where n = 4). Data are presented as Mean ± SD. Statistical significance is represented by letters indicating the individual effects of the compounds in each channel (*gray letters* for TASK-1 and *blue letters* for K_V_1.5). There are no significant differences between drug blockades in a certain channel when they share the same letter. The combined effect, comparing compound blockades in both channels with another independent compound acting in both channels as well, is indicated with an *asterisk*.

To identify the most effective compounds inhibiting TASK-1 and K_V_1.5 channels, inhibition results were compared (Fig. 5). K_V_1.5 recordings showed varying inhibition, significantly lower than in TASK-1 (*p*-value, <2e-16. Table S6). Compounds **6f**, **6e**, **7a**, and **7b** demonstrated the highest activity in K_V_1.5, with blocking percentages of 55.75 ± 11.54%, 42.22 ± 21.23%, 38.04 ± 16.10% and 36.86 ± 13.63%, respectively. Conversely, **7c**, **6a**, **6b**, **6c**, **7d**, and **6d** showed lower inhibition ranging from 35.68 ± 18.28% (**7c**) to 12.31 ± 6.01% (**6d**). For TASK-1, all compounds blocked over 77% with **7c**, **6a**, and **6f** showing the highest inhibition at 89.34 ± 6.56%, 88.32 ± 6.57% and 84.09 ± 10.69%, respectively. Upon comprehensive comparison of the results, it becomes evident that compounds **6e**, **6f**, **7a**, **7b**, and **7c** displayed superior dual-channel activities.

In terms of statistical significance, there are no differences in the block in TASK-1 for the 10 different compounds (gray letters in Fig. 5). Regarding K_V_1.5, **6d** has no statistically significant differences compared to **6c** and **7d**, but it does with the other seven compounds because its blocking activity is the lowest (blue letters in Fig. 5). When comparing the combined effect of each drug in both channels, **6d** exhibits statistically significant differences with **6f** block (*p*-value 0.0417, Table S6).

**Figure 6:**
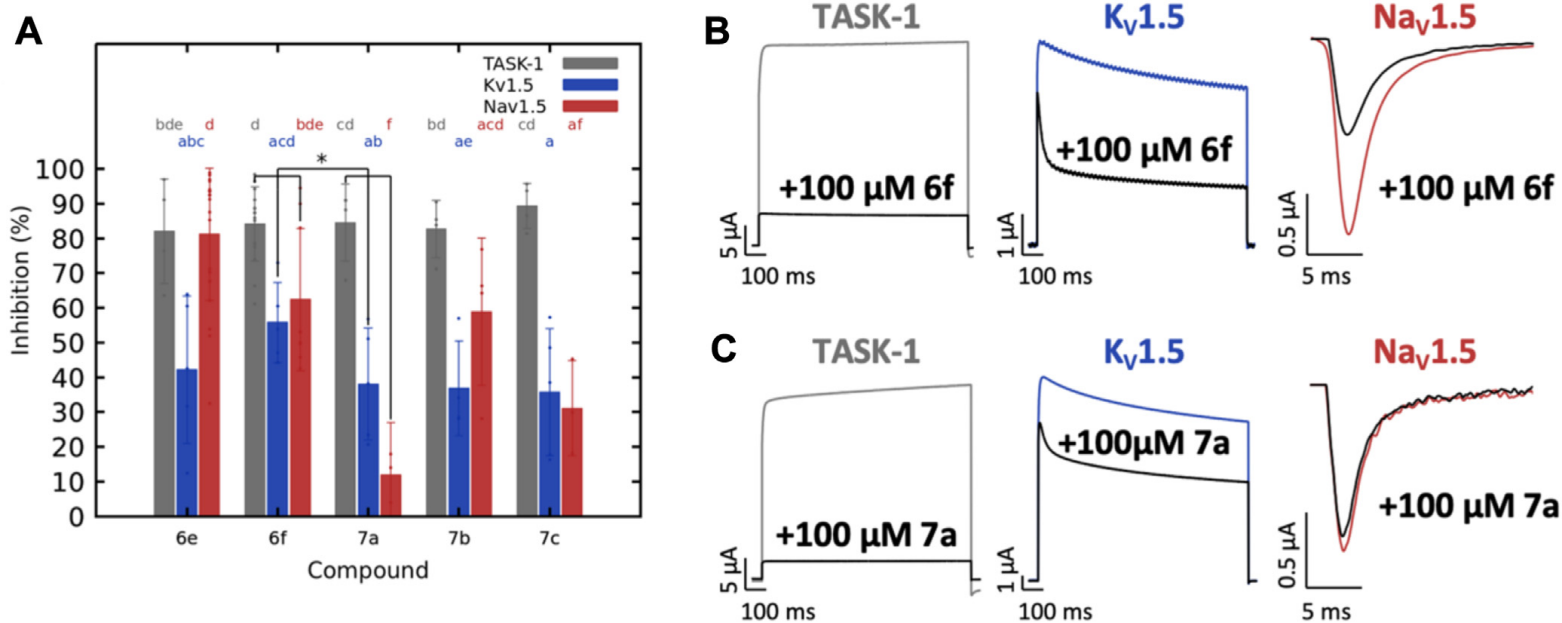
**Inhibitory effects of compounds 6e, 6f, 7a, 7b and 7c on TASK-1, K_V_1.5 and Na_V_1.5 channels**. *A*, analyses of channel inhibition by 100 μM **6e, 6f, 7a, 7b** and **7c**. For TASK-1 and K_V_1.5, currents were analyzed at +40 mV and for Na_V_1.5 at −30 mV. Data are presented as Mean ± SD. Statistical significance is represented by letters indicating the individual effects of the compounds in each channel (*gray letters* for TASK-1, *blue letters* for K_V_1.5, and *red letters* for Na_V_1.5). There are no significant differences for drug block in a certain channel when they share the same letter. The combined effect, comparing compound blockades in the three channels with another independent compound acting in the three channels as well, is indicated with an *asterisk*. Na_V_1.5; **6e** n = 20, **6f** n = 9, **7a** n = 3, **7b** n = 4, **7c** n = 3; for TASK-1 and K_V_1.5, see Figure 5. Representative current traces before and after application of 100 μM of the compound **6f** (*B*) and compound **7a** (*C*).

The five most active compounds on TASK-1 and K_V_1.5 were evaluated on Na_V_1.5 (Fig. 6*A*). At 100 μM, compounds **6e**, **6f**, and **7b** showed the highest inhibitions at 81.19 ± 19.00%, 62.39 ± 20.55%, and 58.91 ± 21.24%, respectively. Compounds **7c** and **7a** had the lowest effects at 31.10 ± 13.67% and 11.93 ± 14.99%, respectively, with **7a** approaching insignificance. Structurally, **6e** and **6f** share an acetamide nucleus and butyl chains, differing by methoxyl groups attached to the aromatic ring: **6e** has one (4-position), while **6f** has two (2- and 4-positions). Compound **7 b**, with a propanamide nucleus and a methyl group (2-position), retains butyl chains (Fig. 4). Compound **6f** emerges as the most homogeneous in terms of simultaneous blocking activity across all three channels. Additionally, in the ranking of the 25 compounds with the highest polypharmacological potential on TASK-1, K_V_1.5, and Na_V_1.5 channels (Table 1), **6f** ranks second. Consequently, compound **6f** was selected for further studies.

In terms of statistical significance, compounds in the Na_V_1.5 channel exhibit varying levels of inhibition but not very different from TASK-1 inhibition (*p*-value, 0.00845. Table S6) as with K_V_1.5 inhibition (*p*-value < 0.001. Table S6). Analyzing the block in Na_V_1.5 for the five different compounds (red letters in Fig. 6), **7a** has no statistically significant differences compared to **7c**, but it does with the other three compounds because its blocking activity is the lowest. When comparing the combined effect of each drug in the three channels (Na_V_1.5, K_V_1.5 and TASK-1), **7a** exhibits statistically significant differences with **6f** block (*p*-value 0.01115, Table S6). These differences are particularly notable in K_V_1.5 and Na_V_1.5 blockade, as shown in the representative current traces before and after application of 100 μM of both compounds (Fig. 6, *B* and *C*).

Regarding the voltage-dependence of channel inhibitions by the selected compound (**6f)**, no significant voltage-dependence was observed for the block of TASK-1 and K_V_1.5 (Fig. S2). However, in Na_V_1.5, small but significant variations were observed in block at different voltages and the conductance-voltage relationships before and after **6f** application (Fig. S3, *A*–*C*) resembling the behavior of other Na^+^ channels blockers such as bupivacaine (39) and propofol (44). On the other hand, we did not observe a significant frequency-dependent inhibition of the Na_V_1.5 channel by **6f** (Fig. S3, *D*–*F*).

#### Affinity of 6f for TASK-1, K_V_1.5, and Na_V_1.5

**Figure 7:**
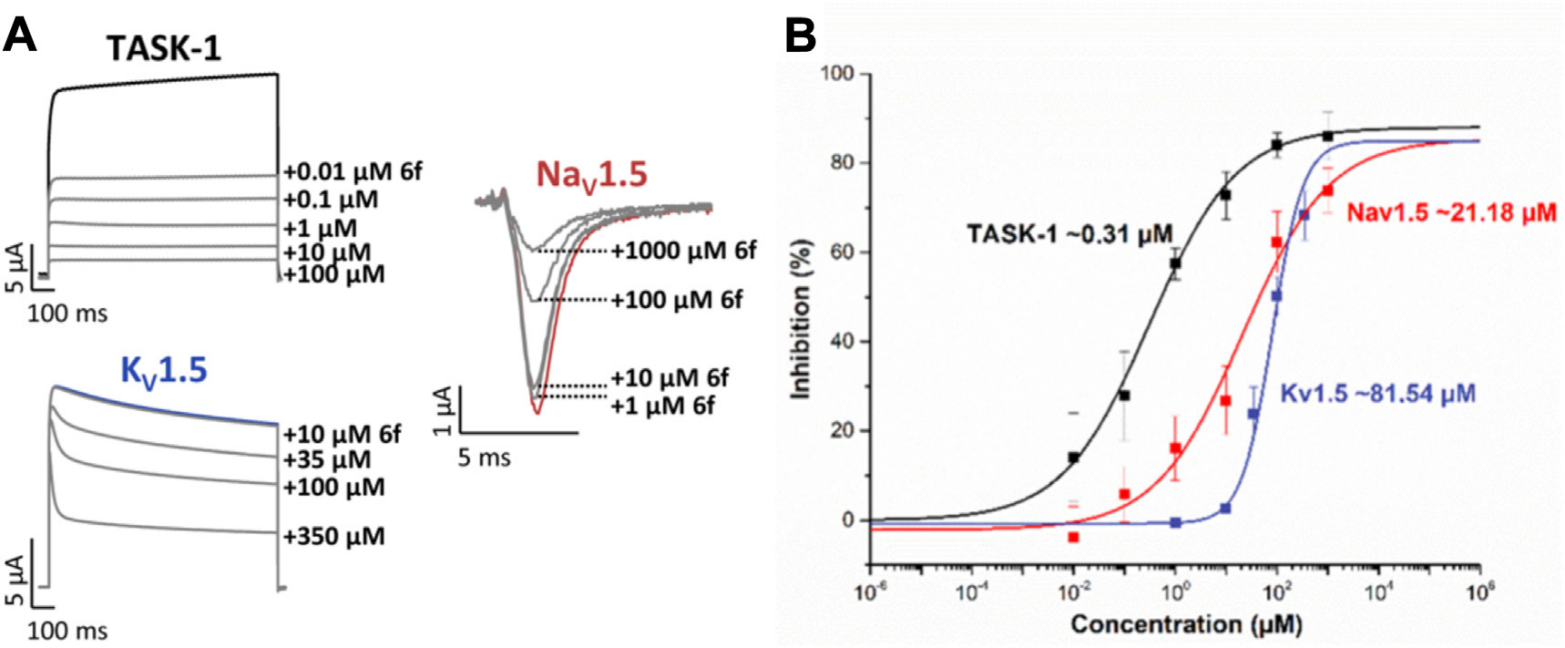
**IC**_**50**_ **of compound 6f in TASK-1, K**_**V**_**1.5 and Na**_**V**_**1.5 channels**. *A*, representative current traces before and after application of increasing concentrations of compound **6f** for TASK-1, K_V_1.5 and Na_V_1.5. *B*, dose-response curves were used to calculate the half-maximal inhibition (IC_50_ values) of compound **6f** for TASK-1, K_V_1.5 and Na_V_1.5 channels. Block was analyzed at +40 mV. For Na_V_1.5, the block was determined at −30 mV. For TASK-1 and Na_V_1.5, a concentration range from 0.01 μM to 1000 μM was used; for K_V_1.5, it was 1 μM to 1000 μM. n = 6 to 15.

To determine IC_50_ values for compound **6f**, dilution series were prepared. For K_V_1.5, concentrations were: 1.0 μM, 10 μM, 35 μM, 100 μM, 350 μM, and 1000 μM. For TASK-1 and Na_V_1.5, concentrations were: 0.01 μM, 0.1 μM, 1.0 μM, 10 μM, 100 μM, and 1000 μM. Experiments were repeated a minimum of 3 times (n = 6). Figure 7 shows IC_50_ curves for all three channels, with values in μM.

Table 2 compares IC_50_ values of **6f** with lidocaine, bupivacaine, and ropivacaine. Compound **6f** shows superior activity in TASK-1 compared to these LA. For K_V_1.5, **6f** IC_50_ is lower than ropivacaine but higher than bupivacaine; no lidocaine data is available for comparison. In Na_V_1.5, **6f** surpasses lidocaine and ropivacaine. IC_50_ values for LA in TASK-1 and ropivacaine in Na_V_1.5 were calculated in HEK-293 cells. While **6f** values were measured in *X. laevis* oocytes. IC_50_ values may differ between systems, potentially being lower in HEK-293 cells (38, 45).

**Table 2:** Reported IC_50_ values for LA *versus* Compound **6f**

Ionic channel	Lidocaine	Ropivacaine	Bupivacaine	Compound 6f
TASK-1	53.8 μM^a^ (38)	17.6 μM^a^^,^^b^ (38)	12.1 μM^a^^,^^b^ (38)	0.31 μM^c^
K_V_1.5	NR	128.9 μM^c^^,^^d^ (15)	31 μM^c^^,^^b^ (15)	81.5 μM^c^
Na_V_1.5	57.9 μM^c^^,^^e^	322.2 μM^a^^,^^b^^,^^f^ (40)	4.5 μM^c^^,^^b^ (39)	21.2 μM^c^

NR: Not reported.

a Measurements in HEK-293 cells.

b Racemic (R/S) compound.

c Measurements in *X. laevis* oocytes.

d S enantiomer.

e Data obtained in the current study

f IC_50_ measured in Na_V_1.5 open state, as was measured using our pulse protocol.

To test the effect of **6f** on atrial ion channels expressed in HEK-293 cells, the compound was assayed at 10 μM and 100 μM. It was observed that **6f** inhibits TASK-1 by 70% at both concentrations. Therefore, the saturation would occur at concentrations equal to or lower than 10 μM, which should be further investigated with an IC_50_ curve. On the other hand, K_V_1.5 and Na_V_1.5 exhibit significant blockade by 6f only at 100 μM in HEK cells (Fig. S1).

These results validate the proposed polypharmacological ranking. Compound **6f**, the top-performing candidate, showed the highest activity across all three channels and ranked second overall (Table 1).

For K_V_1.5, **6f** ranked fourth in CC dockings (Table S1) and 10th in side-pocket dockings (Table S2). Compounds **6e**, **7b**, and **7c,** with similar K_V_1.5 activity (Fig. 6) ranked above 10 in CC dockings but second, third, and sixth in side-pocket dockings. Compound **7a** led CC dockings and ranked ninth in Kv1.5 SP dockings (Tables S1 and S2).

For TASK-1, **6f** ranked 10th (Table S3). Leading this ranking are compounds such as **7a**, **7b**, **6a**, **6c**, and **6d**, which had similar TASK-1 blocking percentages (Fig. 5).

In Na_V_1.5, **6f** ranked first (Table S4), with **6e** also in the top 10 and with a similar—perhaps even higher—blocking percentage in the Na_V_1.5 channel (Fig. 6).

#### Selectivity

**Figure 8:**
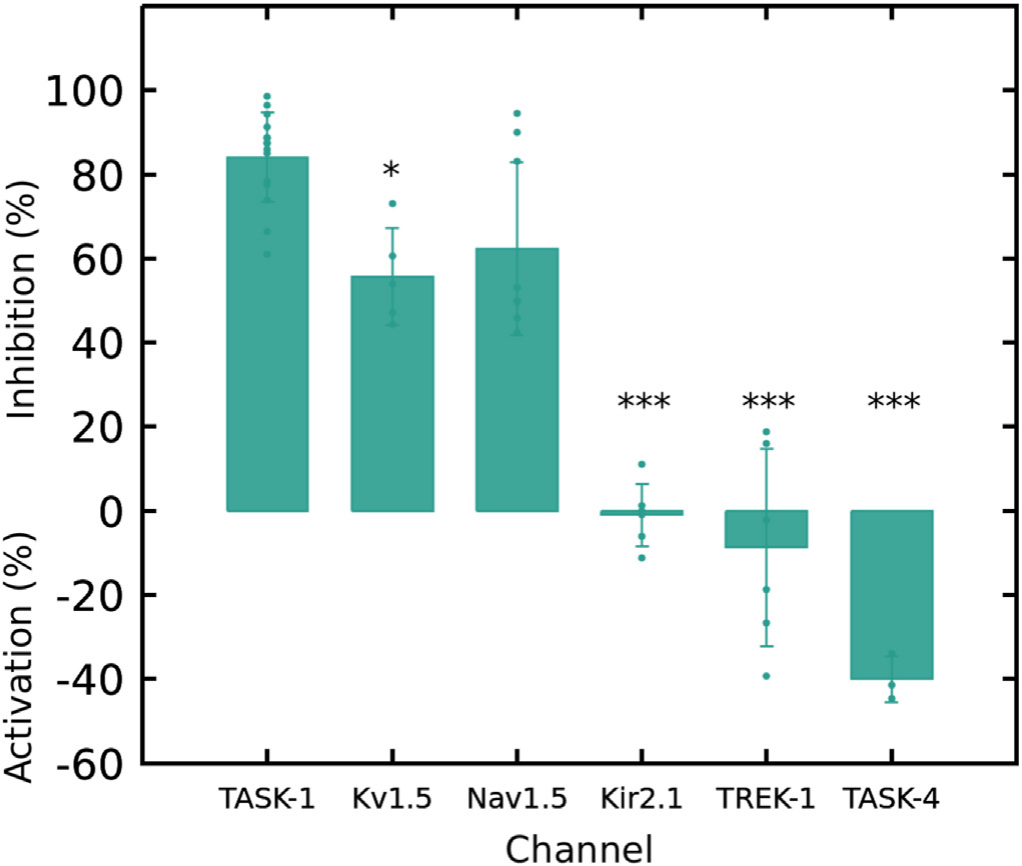
**The activity of compound 6f on different channels was analyzed at100 μM by TEVC in *X. laevis* oocytes.** All data is presented as mean ± SD. The experiments were replicated as follows; TASK-1 n = 4, K_V_1.5 n = 5, Na_V_1.5 n = 9, K_ir_2.1 n = 3, TREK-1 n = 4 and TASK-4 n = 3.

After confirming compound **6f** simultaneous blockade of TASK-1, K_V_1.5, and Na_V_1.5, other cardiac channels (K_ir_2.1, TREK-1, TASK-4) were tested for selectivity at 100 μM (Fig. 8).

The K_ir_2.1 channel, present in cardiac myocytes (46), generates repolarization current in ventricular AP and is downregulated in cardiac failure (47) but upregulated in AF (48). Compound **6f** had no significant effect on this channel (−1.01% ± 7.48%).

Another channel related to ventricular repolarization, the TREK-1 channel, was evaluated. Its role in the heart is controversial (49), but is expressed in both atria and ventricles (50, 51), with higher expression in the ventricles (52). Inhibition of TREK-1 has been linked to arrhythmogenic effects (49, 53). Antiarrhythmic drugs such as dronedarone (54), ranolazine (55), lidocaine (56), and carvedilol (57) block TREK-1 currents, though the clinical relevance of this block remains unclear (49). Moreover, the downregulation of TREK-1 occurs in the AF model due to cardiac remodeling (52, 58). The compound **6f** did not inhibit the channel; but showed a non-significant activating effect (−8.71% ± 23.54%).

Finally, the TASK-4 channel, a cardiac K_2P_ channel and a pharmacological target for atrial and ventricular arrhythmias (59), was evaluated. TASK-4 is primarily expressed in the atria (18), with reduced expression in heart failure and AF patients (19), suggesting channel activation as a potential antiarrhythmic strategy (59). Compound **6f** (100 μM) moderately activated TASK-4 (40.0% ± 5.44%, n = 3), similar to previous studies with antiarrhythmics like vernakalant (59, 60).

To summarize, **6f** predominantly blocked TASK-1, K_V_1.5, and Na_V_1.5 channels, showing a higher affinity for these channels, except for TASK-4, where it showed activation.

### Patch-clamp recordings on isolated human atrial cardiomyocytes

Compound **6f**, the most effective blocker of the TASK-1, K_V_1.5, and Na_V_1.5 channels in *X. laevis* oocytes was further evaluated in patch-clamp experiments on single atrial cardiomyocytes isolated from tissue samples of patients undergoing cardiac surgery for Coronary Artery Bypass Grafting (CABG), Aortic Valve Replacement (AVR) or heart transplantation (Table S7).

**Figure 9:**
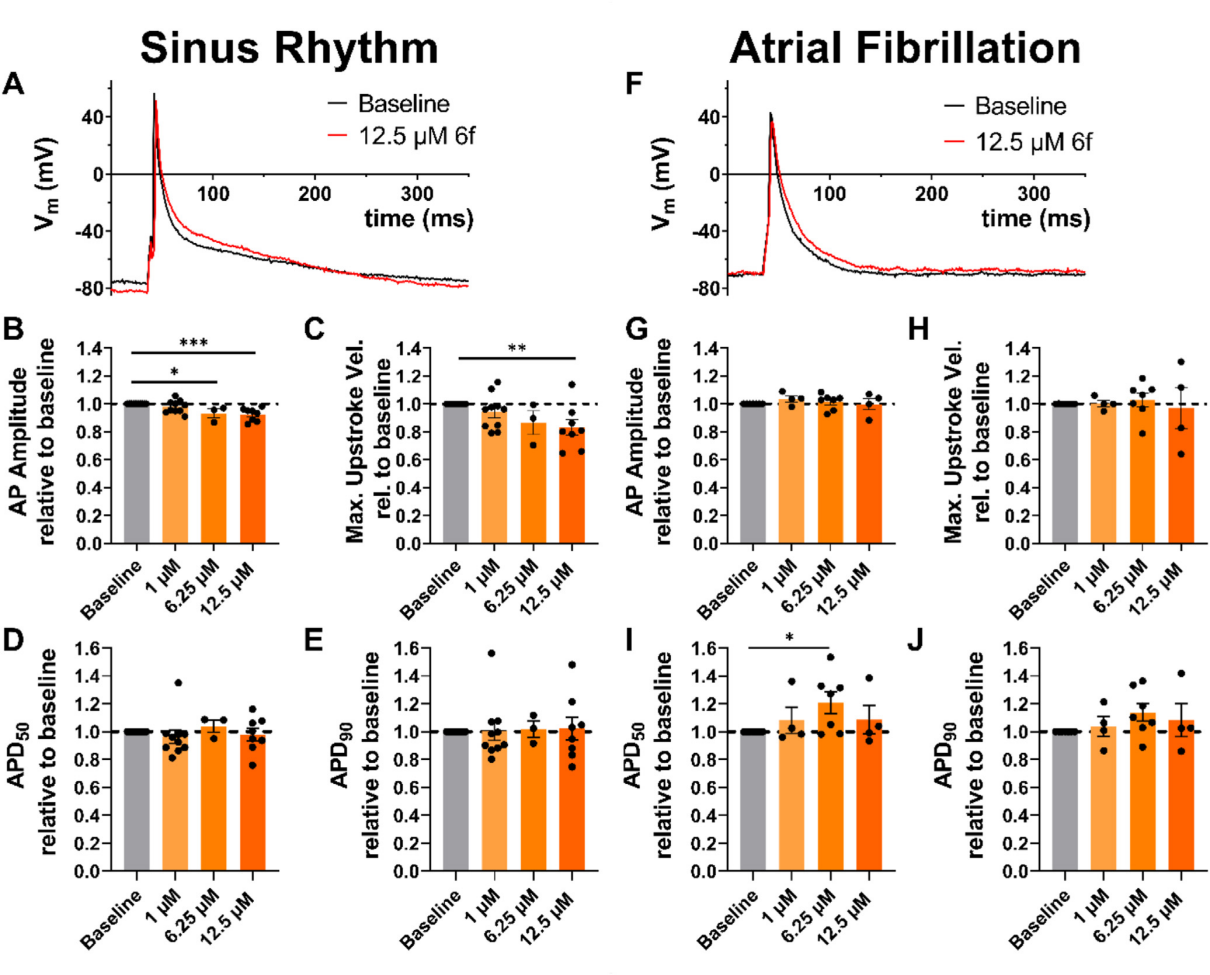
**Comparative analysis of patch-clamp recordings of single atrial cardiomyocytes isolated from tissue samples of patients in sinus rhythm and with atrial fibrillation**. *A–E*, *Atrial action potential (AP) recordings from sinus rhythm (SR) patient samples (n = 13 cells/N = 5 patients). A,* representative AP traces from patch-clamp recordings performed on isolated human atrial cardiomyocytes under basal conditions (*Baseline, black*) and 8 to 10 min after application of 12.5 μM compound **6f** (*red*). *B–E*, recorded changes in AP indices (AP amplitude, maximum upstroke velocity, APD_50_ and APD_90_) relative to baseline after application of compound **6f** at concentrations of 1 μM, 6.25 μM and 12.5 μM. *F–J, AP recordings from atrial fibrillation (AF) patient samples (n = 7 cells/N = 5 patients). F*, representative AP traces under basal conditions (*Baseline, black*) and 8 to 10 min after application of 12.5 μM compound **6f** (*red*). *G–J*, recorded changes in AP indices (AP amplitude, maximum upstroke velocity, APD_50_ and APD_90_) relative to baseline after application of compound **6f** at concentrations of 1 μM, 6.25 μM and 12.5 μM. Data are given as mean ± standard error of the mean. Individual columns were compared to baseline using a mixed effects model with Dunnett’s post-hoc-test. ∗indicates a *p*-value of <0.05, ∗∗ a *p* value of <0.01 and ∗∗∗ a *p*-value of <0.001.

In cardiomyocytes obtained from patients with sinus rhythm (SR), we observed a concentration-dependent decrease in the action potential amplitude (APA) and in the maximal upstroke velocity (dV/dt_max_) compared to baseline that reached statistical significance at a concentration of 12.5 μM (*p* < 0.05) (Figs. 9, *A*–*C* and S4, *A* and *B*), which is consistent with the Na_V_1.5 block that we have observed by **6f** in TEVC recordings (Fig. 7, (61)). In contrast, only minor effects on the APD_50_ and APD_90_ were recorded (Figs. 9, *D* and *E* and S4, *D* and *E*), with no clear trend towards a concentration-dependent increase or decrease.

In cells from patients with AF, no changes in APA or maximal upstroke velocity were observed compared to baseline (Figs. 9, *F*–*H* and S4, *F* and *G*). However, a differential effect on APD was noted compared to SR patients. Previous studies have shown that TASK-1 upregulation in atrial cardiomyocytes of AF-patients has been linked to AP shortening (18, 19). Consistent with our voltage-clamp experiments showing **6f** high affinity for TASK-1, both APD_50_ and APD_90_ showed a concentration-dependent prolongation trend in the AF cohort (Figs. 9, *I* and *J* and S4, *I* and *J*), though not statistically significant at 1 μM and 12.5 μM. The increase in ADP_50_ with 6.25 μM **6f** was significant.

Additionally, in SR cardiomyocytes, a slight increase in the resting membrane potential (RMP) was observed at 6.25 μM of **6f**. This effect was not present at 2.5 μM or 12.5 μM (Fig. S4*C*). However, in AF patients, a significant concentration-dependent shift in the RMP to more negative values was observed (Fig. S4*H*), potentially due to TASK-4 activation by **6f** (Fig. 8).

The effects of **6f** in atrial cardiomyocytes partially resemble those of vernakalant, an atrial-selective antiarrhythmic that reduces APA by blocking Na^+^ channels (62). Additionally, vernakalant activates TASK-4 channels (60) and blocks K_V_1.5 channels (63), but does not significantly reduce TASK-1 currents (64). The overall effect of vernakalant on action potential duration is small in human right atrial tissue (65).

**Figure 10:**
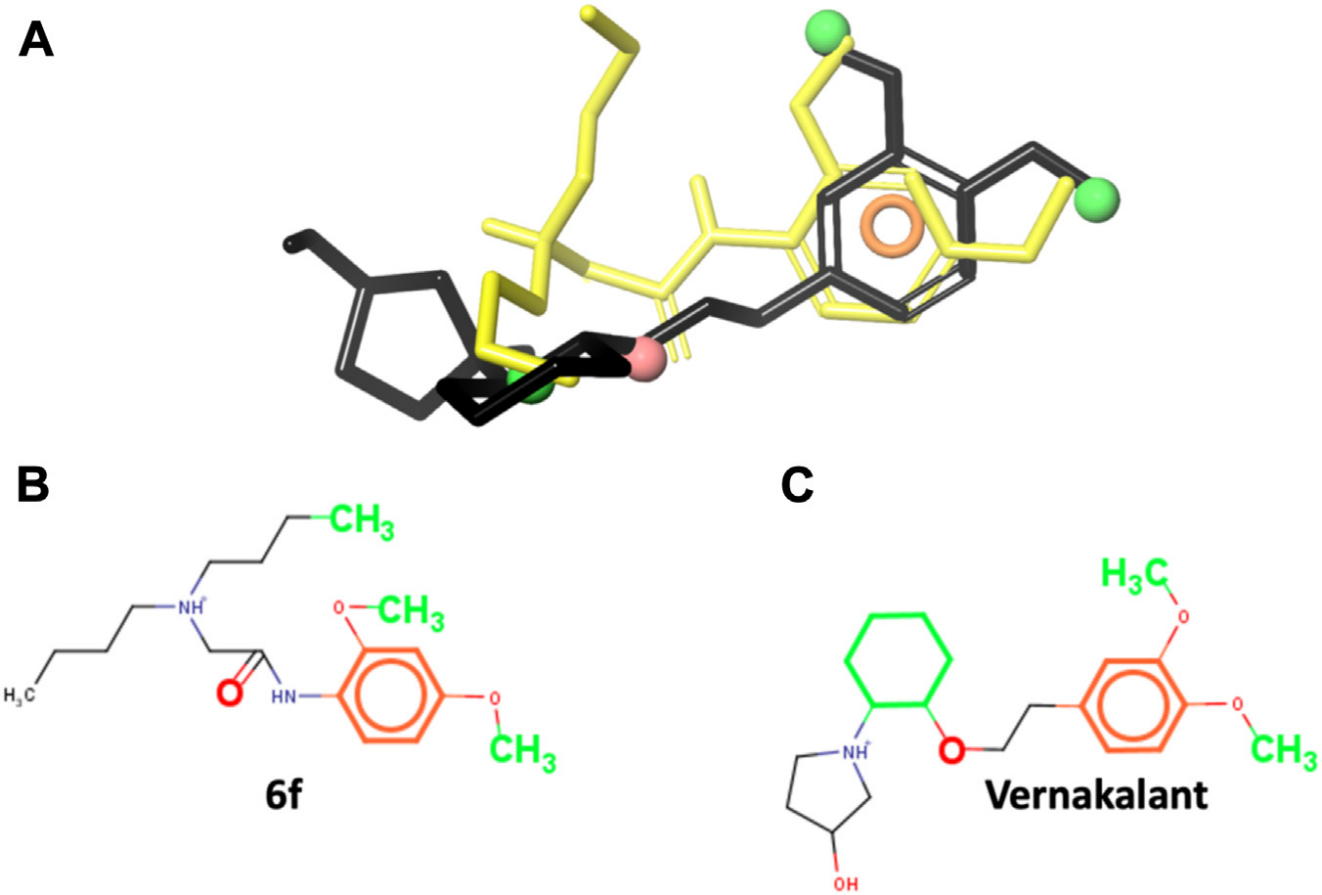
**Common pharmacophore to 6f and vernakalant**. *A*, Compounds **6f** (*yellow*) and vernakalant (*black*) are shown in stick representation. *Green* and *red spheres* stand for hydrophobic and hydrogen acceptor groups, respectively. The *Orange halo* represents an aromatic ring. *B* and *C*, Pharmacophoric features represented in **6f** and vernakalant, respectively, using the same colors as in (*A*). Notice that the NH atoms of the amide linker (-CONH-) are not present in vernakalant.

These similarities and differences relate to the shared chemical structural features of **6f** and vernakalant (Fig. 10). Both exhibit a common pharmacophore with hydrophobic, hydrogen acceptor groups, and an aromatic ring. These features are also shared by the local anesthetics bupivacaine, ropivacaine, and lidocaine. However, vernakalant lacks the hydrogen donor group (amide linker NH atoms), present in **6f** and local anesthetics (Fig. 1).

### *In-silico* trials for the pharmacological management of AF by compound 6f

**Figure 11:**
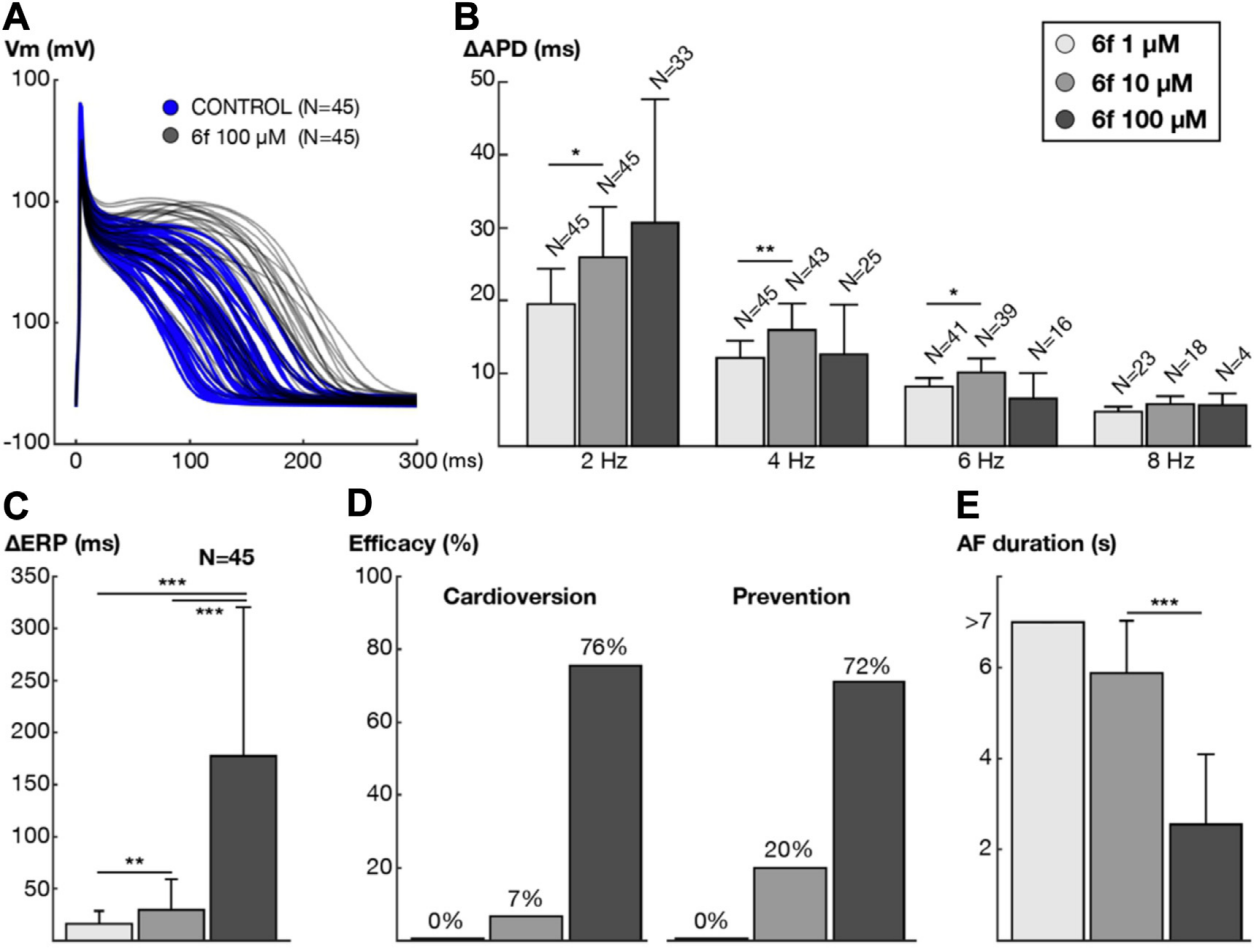
**Impact of three concentrations (1, 10 and 100 μM) of compound 6f on single cell and whole-atria properties**. *A*, AP traces of N = 45 atrial cardiomyocyte models at 2 Hz in control conditions and after the application of compound **6f** at 100 μM. *B*, rate-dependent variation of the APD (ΔAPD). “N” indicates the number of atrial cardiomyocyte models able to propagate the stimulus according to the concentration applied and the pacing rate. *C*, concentrations-dependent increase in the effective refractory period (ΔERP) compared to control conditions in the N = 45 atrial cardiomyocyte models. *D*, AF cardioversion and prevention efficacy (*i.e.*, percentage of AF episodes cardioverted or prevented) in the population of 45 virtual-atria models. *E*, AF duration in the 45 virtual-atria models considering that AF episodes are analyzed for 7 s of activity.

Multi-scale human modeling and simulation were employed to translate and augment the experimental results observed in human atrial cardiomyocytes (Fig. 9) to the patient level. For this, the efficacy of compound **6f** for both acute AF cardioversion and long-term AF prevention was subsequently assessed in a digital twin population of 45 virtual human atria. Figure 11 illustrates the impact of three concentrations (1, 10, and 100 μM) of compound **6f** on the APD (panels A and B), effective refractory period (panel C), efficacy of acute cardioversion, efficacy of long-term AF prevention (panel D), and AF duration (panel E).

Consistent with experimental results in cardiomyocytes from patients with AF, compound **6f** prolonged APD at the cellular level (142.2 ± 30.7 *versus* 181.6 ± 38.8 for control *versus* **6f** at 100 μM at 2 Hz; N = 45; Fig. 11*A*). Subsequently, the *in silico* atrial cardiomyocytes were paced on a one-dimensional cable to assess propagation properties and refractoriness. At moderate frequencies (*i.e.*, 2 Hz), the virtual administration of compound **6f** induced a concentration-dependent prolongation of the APD. At higher pacing frequencies, fewer cardiomyocyte models propagated the stimulus after applying **6f** at 100 μM, due to longer post-repolarization refractoriness (87% [39/45] *vs.* 35% [15/45]) of cardiomyocyte models propagating the stimulus at 6 Hz after 10 μM *vs.* 100 μM of compound **6f**; Fig. 11*B*). At these rates, pronounced I_Na_ inhibition prevailed over the blockade of potassium currents (*I*_Kur_ and *I*_K2P_), causing greater APD prolongation at 10 μM than 100 μM (Fig. 11*B*; from 4 Hz to 6 Hz).

The concentration-dependent prolongation of post-repolarization refractoriness is depicted in Figure 11*C*. Application of 1 μM, 10 μM, and 100 μM of **6f** increased the refractory period by 16.3 ± 12.3 ms, 29.3 ± 29.5 ms, and 177.6 ± 143.0 ms, respectively, compared to control. This elevation in refractoriness led to a proportional enhancement in the effectiveness of compound **6f** for rhythm control of AF (Fig. 11, *C* and *D*). Consequently, its virtual administration to a population of 45 whole-atria models resulted in a concentration-dependent increase in AF cardioversion and prevention efficacy (Fig. 11*D*). This effect was also evident in the overall reduction in AF duration following application of compound **6f** at 100 μM (Fig. 11).

**Figure 12:**
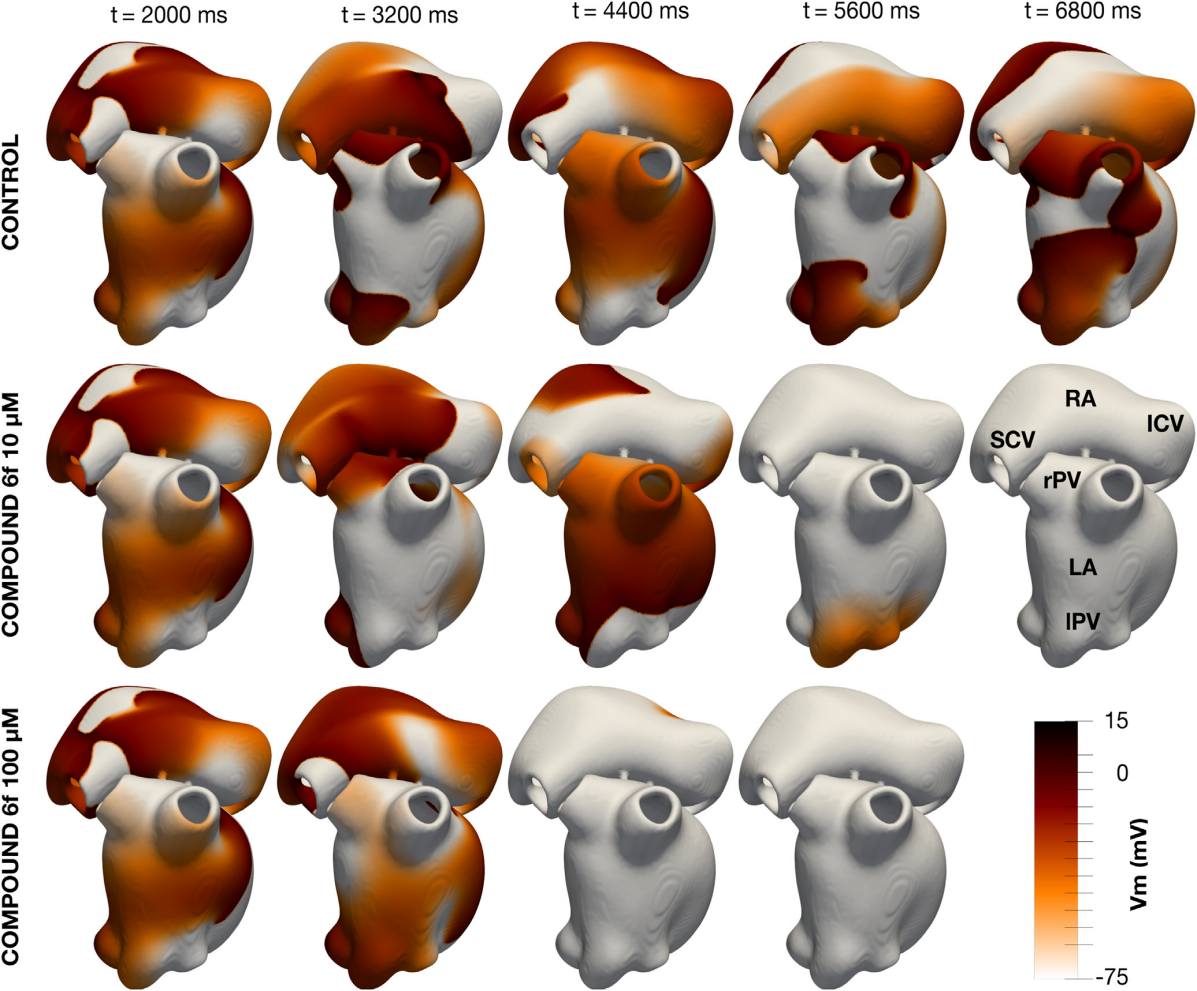
**Consecutives snapshots of the transmembrane voltage (V_m_) for a representative AF episode in control conditions and after the virtual administration of 10 and 100 μM of compound 6f.** RA-LA: *Right* and *left* atrium; SCV-IVC: Superior and inferior cava vein; rPV-lPV: *Right* and *left* pulmonary veins.

Figure 12 presents a representative AF episode under control conditions and after 10 and 100 μM **6f** administration, showing reduced AF burden with increasing concentrations. The drug was administered 2 s after AF initiation, representing acute AF cardioversion.

### Predictions of pharmacokinetics

To evaluate the suitability of the synthesized compounds as drugs, their physicochemical and pharmacokinetic properties were analyzed using the SwissADME platform (66). The results are presented in Table 3, alongside LA properties for comparison. These analyses provide theoretical prediction of the molecules' viability according to Lipinski's rule of five (67) and pharmacokinetic parameters.

**Table 3:** Pharmacokinetic parameters of the evaluated compounds vs LA

Compound	Physicochemicals			Pharmacokinetics							Lipinski rule violations
	DH^a^	AH^b^	LogPo/w^c^	GIA^d^	P-gp^e^	CYP1A2^f^	CYP2C19^f^	CYP2C9^f^	CYP2D6^f^	CYP3A4^f^	
**6a**	1	2	3.53	High	No	Yes	No	No	Yes	Yes	0
**6b**	1	2	3.81	High	No	Yes	No	No	Yes	Yes	0
**6c**	1	2	3.92	High	No	Yes	No	No	Yes	No	0
**6d**	1	2	3.15	High	No	No	No	No	Yes	No	0
**6e**	1	3	3.24	High	No	Yes	No	No	Yes	No	0
**6f**	1	4	3.36	High	No	Yes	No	No	Yes	No	0
**7a**	1	3	3.62	High	Yes	Yes	No	No	Yes	No	0
**7b**	1	2	3.92	High	Yes	Yes	No	No	Yes	No	0
**7c**	1	2	4.54	High	Yes	Yes	Yes	Yes	Yes	Yes	0
**7d**	1	2	4.30	High	Yes	No	No	No	Yes	Yes	0
lidocaine	1	2	2.50	High	No	No	No	No	Yes	No	0
ropivacaine	1	2	3.05	High	No	No	No	No	Yes	No	0
bupivacaine	1	2	3.40	High	No	No	No	No	Yes	No	0

a DH: N° hydrogen donor bonds (≤5).

b AH: N° hydrogen acceptor bonds (≤10).

c Log Po/W: Octanol/water partition coefficient (≤5).

d GIA: Gastrointestinal Absorption.

e P-gp: Permeability glycoprotein substrate. If Yes, it is substrate for P-gp.

f CYP: Isoenzymes belonging to the cytochrome P450. If No, it is not an inhibitor of a given CYP.

None of the synthesized compounds or the LA violate Lipinski's rule: poor absorption or permeation with more than 5 hydrogen bond donors, 10 hydrogen acceptors, molecular weight over 500 Da, or octanol/water partition coefficient above 5 (67). All compounds showed potential for gastrointestinal absorption (GIA), suggesting suitability for oral drug development.

Their behavior as substrates for the permeability glycoprotein (P-gp), crucial for xenobiotics efflux across biological membranes (68), and interactions with cytochrome P450 (CYP) isoenzymes were also assessed. This information enables us to determine the likelihood of these molecules to interact through CYP inhibition and identifies which specific isoforms are affected (66) because being a substrate for P-gp and/or not inhibiting CYP isoforms is key in biotransformation and elimination of the drug (69). In this context, all compounds exhibit characteristics of being P-gp substrates and/or can undergo processing by CYPs. This information holds significant relevance, as CYP and P-gp can collaboratively process small molecules, contributing to tissue protection—a vital aspect concerning drug bioavailability and toxicity.

This analysis suggests that the synthesized compounds could potentially evolve into effective drugs with favorable bioavailability and minimal toxicity.

### Cytotoxicity assays

Assays in cardiomyocytes and *X. laevis* oocytes alone cannot fully determine a compound’s safety, as they do not cover all effects across cell types and tissues. Comprehensive *in vitro* and *in vivo* assays are essential to evaluate safety and efficacy in clinical contexts.

A key method to assess cytotoxicity is hemolysis, which evaluates erythrocyte rupture and hemoglobin release. Since free hemoglobin in the plasma can potentially cause damage to various vital organs, this poses a risk of toxicity and affects the routes of drug administration, including intravenous and other methods (65). Therefore, hemoglobin release was quantified for the most active compounds in K_V_1.5 and TASK-1, selected for further analysis in Na_V_1.5 (Fig. 6), to assess membrane disruption potential.

**Figure 13:**
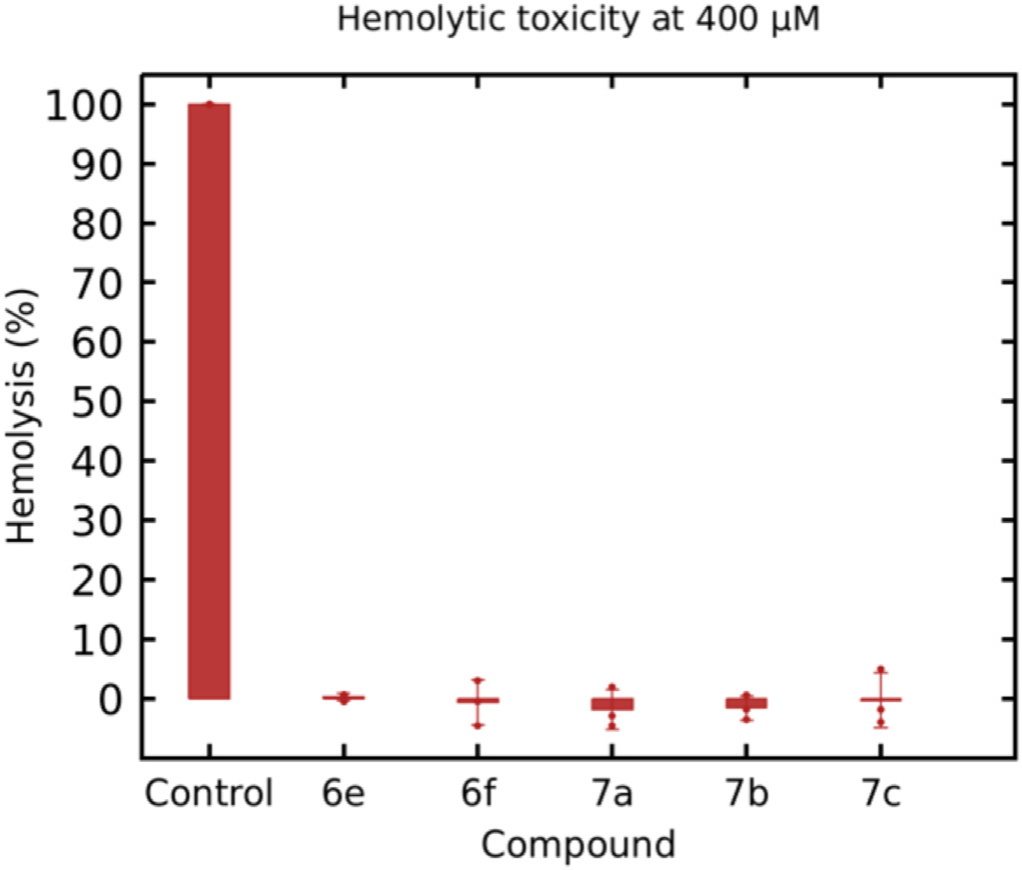
**Hemolytic activity of compounds 6e, 6f, 7a, 7b and 7c at 400 μM in human erythrocytes measured at 504 nm**. All data are presented as mean ± SD. The experiments were in triplicate n = 3.

Erythrocytes treated with tween-20 and phosphate buffer were used as 100% and 0% hemolysis values, respectively. Red blood cells (RBCs) incubated with compounds **6e, 6f, 7a, 7b,** and **7c** (0.2–400 μM) for 2 hours showed no hemolytic effect, suggesting no detectable membrane disruption. Results at 400 μM are shown in Figure 13.

**Figure 14:**
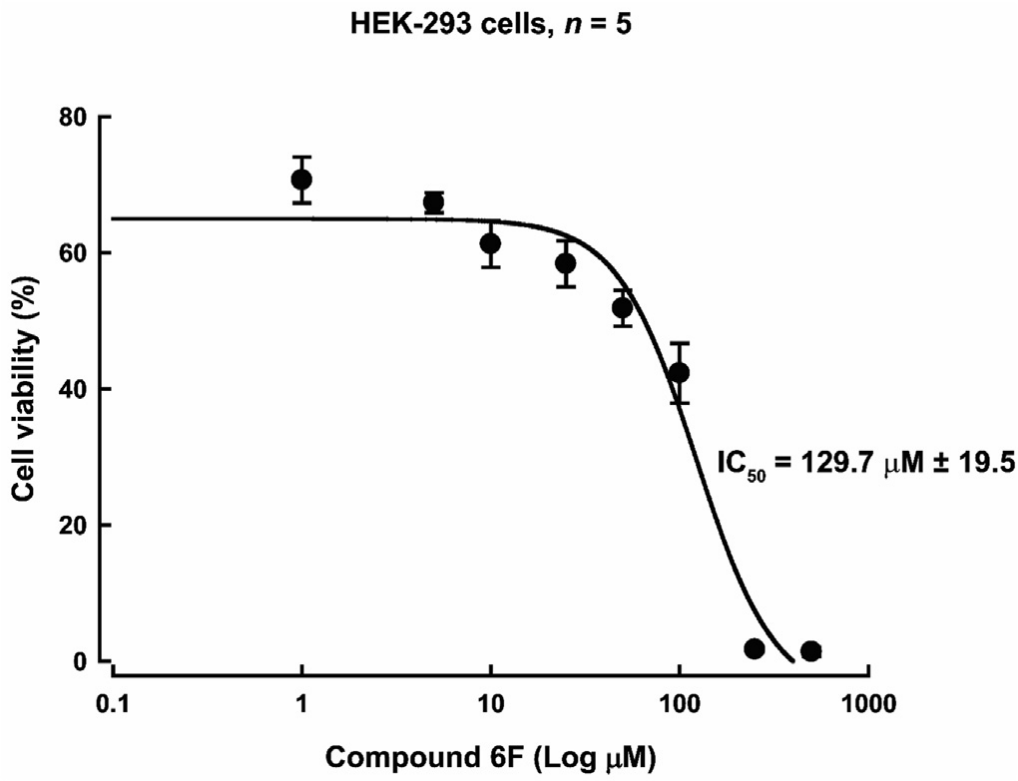
**Dose-response curve of compound 6f on HEK-293 cells. HEK-293 cells were treated for 72 h with increasing concentrations of compound 6f**. Cell viability was assessed using the MTT assay, and the data were normalized to the positive control (cells cultured in medium supplemented with 10% FBS, set as 100% viability). The dose-response curve was fitted using a nonlinear regression model with a four-parameter logistic equation using SigmaPlot version 12.0. The data are represented as the mean ± standard deviation, from five independent experiments.

Another method to assess cytotoxicity is the MTT assay [3-(4,5-dimethylthiazol-2-yl)-2,5-diphenyltetrazolium bromide], which assesses the metabolic activity of HEK-293 cells through formazan formation (70). Compound **6f** cytotoxicity was also evaluated using MTT assay (Fig. 14) and the results showed a dose-dependent reduction in viability on HEK cells, with significant cytotoxic effects observed at concentrations higher than the half-maximal inhibitory concentration (IC_50_ = 129.7 μM).

## Discussion

In this study, 60 molecules were rationally designed based on the pharmacophore for LA, known for their antiarrhythmic use and simultaneous blockade of Na_V_1.5, K_V_1.5, and TASK-1 channels. Molecular docking calculations were conducted, assuming binding sites similar to those reported for LA: bupivacaine in TASK-1 (43), bupivacaine and ropivacaine in K_V_1.5 (15), and lidocaine in Na_V_1.5 (42, 71, 72). Using docking and MM-GBSA results, a ranking identified the most promising candidates. Notably, several designed compounds exhibited *in silico* outcomes superior to those of reference compounds.

Having identified the optimal compounds, synthetic pathways were optimized through the assessment of various strategies. The chosen compounds, namely **6a-f** and **7a-d**, were synthesized in two steps: an acylation employing the coupling reagent DIC, followed by a substitution phase. These synthesized compounds were subsequently purified and characterized, yielding moderate to good yields.

Initial biological tests applying compounds at 100 μM on TASK-1 and K_V_1.5 potassium channels *via* two-electrode voltage clamp showed robust current inhibition, particularly in TASK-1. Among the compounds tested, **6e**, **6f**, **7a**, **7b**, and **7c** showed the best inhibitory capacities on both channels. Furthermore, on Na_V_1.5, compounds **6e**, **6f**, and **7b** exhibited the most significant blocking percentages.

Compound **6f** exhibited the most potent blocking activity across TASK-1, K_V_1.5, and Na_V_1.5 channels at micromolar levels, with IC_50_ values of 0.31, 81.5, and 21.18, respectively, often surpassing LA in affinity. This highlights **6f**′s potential as a multi-target agent, although it clearly showed high affinity for TASK-1 channels (IC_50_ in the submicromolar range) and low affinity for K_V_1.5 and Na_V_1.5 channels. Evaluations on other cardiac channels such as K_ir_2.1 and TREK-1 revealed either negligible or minimal activity, while on TASK-4, **6f** exhibited ∼40% activation at 100 μM.

In patch-clamp recordings on isolated human atrial cardiomyocytes, we detected a concentration-dependent decrease in APA and maximal upstroke velocity in SR patients, consistent with Na_V_1.5 block by **6f**. In samples of patients with AF, 6.25 μM of **6f** significantly increases ADP_50_, aligning with voltage-clamp results showing its high TASK-1 affinity. Additionally, in this cohort, a significant concentration-dependent reduction in RMP towards more negative values was observed, consistent with TASK-4 activation by **6f**. Considering these results, we conclude that **6f** is a polyvalent drug with multiple targets for the treatment of atrial fibrillation (73), although not the three targets initially considered in the design. We expected that a rational design protocol, such as the one proposed here, which focuses on the binding sites of Na_V_1.5, K_V_1.5, and TASK-1 channels, would yield high-affinity blockers for these three channels. However, this approach partially failed, as we observed a clear effect on TASK-1 channels in AF, but not on the other two proposed channels. Surprisingly, we identified another target of **6f**, TASK-4, with **6f** showing effects on two targets in AF cardiomyocytes: TASK-1 and TASK-4.

Patch-clamp recordings on isolated human atrial cardiomyocytes neither confirm nor exclude the possibility that compound **6f** could yield better *in vivo* outcomes compared to current drugs. To address this, a study similar to the one conducted by Paasche *et al.* (61) would be required. That study compared the effects of 1 μM, 10 μM, and 100 μM flecainide and dapagliflozin on atrial-induced pluripotent stem cells using patch-clamp techniques. A comparable study could be performed with 6f and vernakalant, given the similarities in their activity and pharmacophoric features (Fig. 10). However, based on comparative studies such as that of Paasche *et al.*, it can be concluded that compound **6f** exhibits moderate effects in isolated cardiomyocytes, which may not necessarily translate to *in vivo* efficacy.

Simulations of whole-atria showed potentially favorable effects of compound **6f** in AF, including concentration-dependent APD and refractory period prolongation and cardioversion capacity, highlighting its potential for AF management and prevention. Specifically, a cardioversion efficacy of 76% was observed for compound **6f** at the maximum concentration tested (100 μM). This efficacy is superior to or comparable with that obtained in other simulation studies (74, 75) for currently available drugs, such as vernakalant (42–57% efficacy), flecainide (48–70%), or amiodarone (57–80%). Moreover, some of these drugs are associated with several non-cardiac side effects (*e.g.*, amiodarone) or are contraindicated in the context of structural heart disease (*e.g.*, flecainide). Therefore, compound **6f** could represent a safer solution for the pharmacological management of AF. However, it is important to note that 100 μM is typically considered a supratherapeutic concentration. In the referenced study (74), the maximum tested concentrations for vernakalant, flecainide, and amiodarone were 30 μM, 2 μM, and 3 μM, respectively. Future simulation studies could assess compound **6f** at concentrations comparable to vernakalant (30 μM), as its efficacy at 10 μM appears limited for atrial fibrillation (AF) cardioversion.

Physicochemical and pharmacokinetic evaluations of the 10 synthesized compounds revealed no Lipinski's rule violations, including for **6f**, which is metabolized by at least two CYP enzymes. Hemolysis assays confirmed that the most active compounds in K_V_1.5 and TASK-1, further analyzed in Na_V_1.5, do not disrupt membranes even at supratherapeutic concentrations of 400 μM. Additionally, an MTT assay showed significant cytotoxic effects of compound **6f** only at concentrations higher than 129 μM. This evidence supports the synthesized compounds, particularly **6f**, as potential AF drug candidates.

## Experimental procedures

### Computational studies

In summary, we identified drugs with the highest polypharmacological potential by performing induced-fit docking (IFD) of 60 compounds in TASK-1, Na_V_1.5, and K_V_1.5 channels. The grid was centered at fenestrations in TASK-1, the CC of Na_V_1.5, and the CC and SP of K_V_1.5. Binding free energy was calculated for each IFD pose, and compounds were ranked to select the top 10 for synthesis and electrophysiology experiments.

#### Design and selection of the compounds

Based on the LA pharmacophore (71, 76, 77, 78), we designed two series of 60 compounds: 30 derived from bromoacetic acid (Figs. 2*A*) and 30 from (*S*)-2-bromo-propionic acid (Fig. 2*B*). Ligands were drawn using the Maestro workspace and refined using "LigPrep" (Schrödinger, LLC, New York, NY, 2023). The Epik (79) module was employed to establish the protonation form of the amino group. Ligands were energetically minimized using the conjugate gradient method (80) implemented in the Macromodel module.

Protein structures were prepared with Protein Preparation Wizard (81) from the Schrödinger suite for docking. Protonation states were predicted at pH 7.0 with PROPKA (82). Charges and parameters were assigned according to the OPLS-2005 force field.

For TASK-1, we utilized the crystal structure 6RV3, chains A, B (83), and for Na_V_1.5, we used the crystal structure 6LQA (84). As for the K_V_1.5 channel, we employed the homology model proposed by Marzian *et al.*, 2013, which was based on the crystal structures of the open-state chimera K_V_1.2: K_V_2.1 (K_V_Chim) (PDB template: 2R9R) (16). This homology model has been successfully used in several research studies (15, 25, 85). The missing residues in TASK-1 (149–151 from chain A and 150–151 from chain B) were modeled using the "crosslink protein" tool. Additional molecules were removed, retaining only the protein and selectivity filter ions. In K_V_1.5, two ions and two water molecules in the selectivity filter were retained, while Na_V_1.5 was used without any additional molecules.

To perform molecular docking, the induced-fit docking (IFD) protocol in Glide (Schrödinger suite 11.8) (86) was used with the standard precision scoring function, generating up to 10 poses *per* calculation. Grid centers were defined at the geometric center of each LA binding site and adjusted based on side-chain movements during docking refinement. For TASK-1, a single lateral fenestration was explored, as both subunits are symmetrical, based on binding site (BS) residues reported for bupivacaine (43). For K_V_1.5, critical residues for bupivacaine and ropivacaine binding (15) were considered, performing independent dockings for the central cavity (CC) and side pockets (SP). Regarding Na_V_1.5, the analysis focused on the residues reported for lidocaine binding in domains III and IV (42). The BS residues for each channel are shown in Table 4.

**Table 4:** Binding site residues of each channel individualized by chains in TASK-1 (chains A and B) and K_V_1.5 (chains A to D)

Channel	Binding site residues
TASK-1 (43)	A:Q126, A:L171, A:F194, A:T198, A:T199, A:V234, A:I235, A:G236, A:F238, A:L239, A:N240, B:T93, B:M111, B:A114, B:I118
Na_V_1.5 (42)	L1462, I1466, F1760, Y1767
K_V_1.5 (CC) (15)	A:T480, A:I508, A:V512, A:V516, B:T480, B:I508, B:V512, B:V516, C:T480, C:I508, C:V512, C:V516, D:T480, D:I508, D:V512, D:V516
K_V_1.5 (SP) (15)	A:L436, A:F439, A:F440, A:I443, A:I502, A:L510, B:L436, B:F439, B:F440, B:I443, B:I502, B:L510

Approximately 550 docking poses were obtained *per* system, totaling 2212 poses (∼10 per ligand), using 60 designed compounds and 5 validation compounds (R/S bupivacaine, R/S ropivacaine, and lidocaine) for TASK-1, Na_V_1.5, and K_V_1.5 channels. The enantiomers of bupivacaine and ropivacaine were included due to differences in inhibitory activity (15, 36, 37, 38, 40, 41, 87).

Following docking with the IFD method, binding free energy calculations were performed using the MM-GBSA method to re-score and analyze poses. MM-GBSA estimates binding free energy by combining molecular mechanics and implicit solvation, offering intermediate precision and computational efficiency between empirical scoring methods of ligand-protein affinity and perturbation methods of free energy calculation (88).

#### Ranking of poses

To select the best compounds based on two criteria—I. MM-GBSA energy and II. Interaction with key binding residues—we proposed a scoring strategy. After obtaining the binding free energy results, it was determined whether each pose was within 5 Å of BS residues for LA compounds (bupivacaine for TASK-1, bupivacaine/ropivacaine for K_V_1.5, lidocaine for Na_V_1.5). The Schrödinger python API measured distances between protein residues and ligands. Both criteria were normalized: binding free energy received values closer to 1 for more negative energies, and higher interaction counts with BS residues were similarly normalized. A score (NORMT) was calculated as the average of normalized energy (DGBIND_NORM) and interaction (INT_NORM) values. Poses were ranked by "INT_NORM" and then "NORMT" (Tables S1–S4), prioritizing interactions with BS residues over MM-GBSA energy. For each compound, the top pose with the best combined score ("INT_NORM" and "NORMT" values) was selected. For this resulting order, a new variable called "RANK" was assigned, with higher-ranked compounds closer to 1.

#### Ranking of compounds

Once each compound's ranking in each channel was obtained, the "RANK" values were summed to calculate the "GLOBAL RANK," identifying compounds with the best rankings across all three channels. Compounds were then ordered by "GLOBAL RANK", with the lowest values ranked highest, and the top 10 common compounds were selected.

A schematic of the ranking process is shown in Figure 2. The process, implemented in the Python library "polypharm" (89) (available on GitHub: https://github.com/ucm-lbqc/polypharm), is adaptable for other proteins of interest.

### Chemistry

The reagents and solvents used in this study were purchased from Sigma-Aldrich and Merck. The reactions were monitored using thin layer chromatography (TLC) on Al TLC chromatofoils of silica GF254, which were revealed in a SPECTROLINE MODEL CM-10 camera at two wavelengths, 254 and 366 nm. Revealing solutions based on ninhydrin (for primary amines), bromocresol green (for carboxylic acids), sublimated iodine (for unsaturations), among others, were used.

Characterization of the compounds was carried out using different techniques. Infrared (IR) spectra (KBr pellets, 500–4000 cm^-1^) were obtained using a NEXUS 670 FT-IR spectrophotometer (Thermo Nicolet, Madison, WI, USA). Nuclear magnetic resonance (NMR) spectra were recorded on a Bruker DPX 400 spectrometer (400 MHz for ^1^H and 100 MHz for ^13^C). Samples were dissolved in CDCl_3_, and spectra were calibrated using tetramethylsilane (TMS) signals. Displacement (δ) and coupling constant (J) values are reported in parts per million (ppm) and Hertz (Hz), respectively. The signals were represented as follows: singlet (s), doublet (d), triplet (t), and multiplet (m). Melting points (m.p.) (uncorrected) were measured using an IA9100 m.p. electrothermal apparatus (Stone). High-resolution mass spectrometry (HRMS) spectra were obtained on a Bruker Compact QqTOF spectrometer. The analysis was performed by direct injection using a 500 μl Hamilton syringe with a flow rate of 2 μl/min using the InfusionONE Syringe Pump NE-300. For ionization, an ESI source was used, with a voltage of 4500V, a gas temperature of 180 °C, flow at 4 L/min, and a nebulizer at 0.4 bar.

High-Performance Liquid Chromatography (HPLC) analysis was carried out on a YL9100 instrument with a YL9110 quaternary pump, a YL9150 autosampler, a YL9101 degassing pump, and a photodiode array (PDA) detector. Chromatogram processing was performed using Clarity software version 6.0 of 2012. A LiChroCart 125-4 LiChrospher 100 RP-18 reverse phase C-18 column (5 μM) was used. The mobile phase consisted of two solutions: the first (solution A) was a solution of 0.1% trifluoroacetic acid (TFA) in acetonitrile (CH_3_CN), and solution B was an aqueous mixture of 0.01% TFA. The flow rate was 1 ml/min, and the chromatograms were acquired at a wavelength of 254 nm (90).

#### Synthesis of derivatives of bromoacetic acid (6a-f) and (*S*)-2-bromopropionic acid (7a-d)

The amidation reactions were performed using the coupling reagent *N,N′*-diisopropylcarbodiimide (DIC) with bromoacetic acid (**1**) or (*S*)-2-bromopropionic acid (**2**) (Fig. 3). In a round-bottomed flask, a solution of bromoacetic acid (**1**) or (*S*)-2-bromopropionic acid (**2**) (1 mmol, 1 equiv.) in dichloromethane (DCM) was added at 0 °C. Then, DIC (170.33 μl, 1.1 equiv.) was added and stirred for 2 min for **1** or 10 min for **2**. Afterward, the aromatic amine (**3**) (1 mmol, 1 equiv.) was added, and the reaction was monitored using TLC. Once the reaction was complete, purification was carried out through column chromatography, and the pure amide product (**4**) was obtained (90, 91).

In the second phase, 10 mol% potassium iodide (KI) and sodium carbonate (Na_2_CO_3_) (1 mmol, 1 equiv.) were dissolved in 3 ml DMF at 0 °C and added to the amide product. Subsequently, the aliphatic amine (**5**) (1.3 mmol, 1.3 equiv. for **1** or 2.3 mmol, 2.3 equiv. for **2**) dissolved in DCM was added and left stirring for 24 h at room temperature. The reaction was monitored by TLC, and the final product (**6** or **7**) was obtained. Finally, extraction with ethyl acetate (50 ml) was performed, followed by washing with water (20 ml), a saturated solution of sodium bicarbonate (20 ml), and a saturated solution of sodium chloride (20 ml). The mixture was then dried over anhydrous Na_2_SO_4_. The compound was purified by column chromatography using an ethyl acetate:petroleum ether mixture with a variable gradient.

##### 2-(dibutylamine)-*N-*(*o*-tolyl)acetamide (6a)

Yellow liquid; Yield: 68.37%; ^1^H-NMR (400 MHz, CDCl_3_) δ 9.41 (s, 1H), 8.15 (d, *J* = 8.1 Hz, 1H), 7.29 – 7.15 (m, 2H), 7.04 (t, *J* = 7.4 Hz, 1H), 3.20 (s, 2H), 2.57 (t, *J* = 8.1 Hz, 4H), 2.29 (s, 3H), 1.50 (m, 4H), 1.35 (m, 4H), 0.93 (t, *J* = 7.3 Hz, 6H); ^13^C- NMR (100 MHz, CDCl_3_) δ: 169.95 (C), 136.06 (C), 130.33 (CH), 127.00 (CH), 124.22 (CH), 121.02 (CH), 59.56 (CH_2_), 55.42 (CH_2_), 29.78 (CH_2_), 20.58 (CH_2_), 17.79 (CH_3_), 13.99 (CH_3_); DEPT-135 δ 130.33 (CH), 126.92 (CH), 124.22 (CH), 121.02 (CH), 59,56 (CH_2_), 52.91 (CH_2_), 29.78 (CH_2_), 20.58 (CH_2_), 17.79 (CH_3_), 13.99 (CH_3_); IR (KBr): υ: 3302.13, 2958.80, 2862.36, 2819.93, 1697.36, 1589.34, 1523.76, 1454.33, 752.24 cm^-1^; HRMS (ESI, m/z): Calcd for C_17_H_28_N_2_O [M + H]^+^ 277.2280 found 277.2278; HPLC Purity = 96.7%; ((Solution A: (TFA 0.01%/acetonitrile) y (Solution B: TFA 0.01%)), 1 ml/min, tR = 3.367 min).

##### 2-(dibutylamine)-*N*-(2-ethylphenyl)acetamide (6b)

Yellow liquid; Yield: 72.34%; ^1^H-NMR (400 MHz, CDCl_3_) δ 9.46 (s, 1H), 8.14 (d, *J* = 8.0 Hz, 1H), 7.32 – 7.18 (m, 2H), 7.11 (t, *J* = 7.4 Hz, 1H), 3.22 (s, 2H), 2.65 (m, 2H), 2.62 – 2.55 (m, 4H), 1.56 – 1.47 (m, 4H), 1.40 – 1.32 (m, 4H), 1.28 (t, *J* = 7.6 Hz, 3H), 0.94 (t, *J* = 7.3 Hz, 6H); ^13^C-NMR (100 MHz, CDCl_3_) δ 170.09 (C), 135.27 (C), 133.31 (C), 128.56 (CH), 126.80 (CH), 124.73 (CH), 121.45 (CH), 59.34 (CH_2_), 55.39 (CH_2_), 29.72 (CH_2_), 24.60 (CH_2_), 20.60 (CH_2_), 14.24 (CH_3_), 14.00 (CH_3_); DEPT-135 δ 128.56 (CH), 126.80 (CH), 124.59 (CH), 121.73 (CH), 59.54 (CH_2_), 55.39 (CH_2_), 29.72 (CH_2_), 24.44 (CH_2_), 20.60 (CH_2_), 14.24 (CH_3_), 14,00 (CH_3_); IR (KBr): υ: 3305.99, 2870.08, 2360.87, 1693.50, 1585.49, 1519.91, 1454.33, 752.24 cm^-1^; HRMS (ESI, m/z): Calcd for C_18_H_30_N_2_O [M + H]^+^ 291.2436 found 291.2430; HPLC Purity = 99.3%; ((Solution A: (TFA 0.01%/acetonitrilo) y (Solution B: TFA 0.01%)), 1 ml/min, tR = 3.427 min).

##### 2-(dibutylamine)-*N*-(3,5-dimethylphenyl)acetamide (6c)

Yellow liquid; Yield: 69.45%; ^1^H-NMR (400 MHz, CDCl_3_) δ 9.30 (s, 1H), 7.03 (s, 2H), 6.77 (s, 1H), 3.15 (s, 2H), 2.61 – 2.52 (m, 4H), 2.33 (s, 6H), 1.53 – 1.45 (m, 4H), 1.43 – 1.31 (m, 4H), 0.95 (t, *J* = 7.3 Hz, 6H); ^13^C-NMR (100 MHz, CDCl_3_) δ: 169.98 (C), 138.73 (C), 137.66 (C), 125.74 (CH), 116.92 (CH), 59.41 (CH_2_), 55.36 (CH_2_), 29.58 (CH_2_), 21.39 (CH_3_), 20.62 (CH_2_), 14.01 (CH_3_). DEPT-135 δ 125.74 (CH), 116.92 (CH), 59.41 (CH_2_), 55.36 (CH_2_), 29.58 (CH_2_), 21.39 (CH_3_), 20.62 (CH_2_), 14.01 (CH_3_); IR (KBr): υ: 3294.42, 2958.80, 2862.36, 1693.50, 1612.49, 1535.34, 837.11 cm^-1^; HRMS (ESI, m/z): Calcd for C_18_H_30_N_2_O [M + H]^+^ 291.2436 found 291.2427; HPLC Purity = 99.5% ((Solution A: (TFA 0.01%/acetonitrile) y (Solution B: TFA 0.01%))1 ml/min, tR = 3.493 min).

##### 2-(diisopropylamine)-*N*-(3,5-dimetylphenyl)acetamide (6d)

Yellow solid; Yield: 73.65%; m.*p* = 58 to 59 °C; ^1^H- NMR (400 MHz, CDCl_3_) δ 9.43 (s, 1H), 7.23 (s, 2H), 6.78 (s, 1H), 3.18 (s, 2H), 3.13 to 3.17 (m, 2H), 2.34 (s, 6H), 1.11 (d, *J* = 6.5 Hz, 12H); ^13^C-NMR (100 MHz, CDCl_3_) δ: 171.41 (C), 138.77 (C), 137.58 (C), 125.74 (CH), 116.89 (CH), 50.31 (CH_2_, CH), 21.36 (CH_3_), 20.64 (CH_3_); DEPT-135 δ 125.74 (CH), 116.89 (CH), 50.32 (CH_2_), 50.28 (CH), 21.36 (CH_3_), 20.64 (CH_3_); IR (KBr): υ: 3259.70, 2966.52, 2866.22, 2360.87, 1670.35, 1608.63, 1527.62, 775.38 cm^-1^; HRMS (ESI, m/z): Calcd for C_16_H_26_N_2_O [M + H]^+^ 263.2123 found 263.2120; HPLC Purity = 96.6% ((Solution A: (TFA 0.01%/acetonitrile) y (Solution B: TFA 0.01%)), 1 ml/min, tR = 3.740 min).

##### 2-(dibutylamine)-*N*-(4-methoxiphenyl)acetamide (6e)

White solid; Yield: 66.88%; m.*p* = 50 to 51 °C; ^1^H-NMR (400 MHz, CDCl_3_) 9.27 (s, 1H), 7.48 (d, *J* = 8 Hz,2H), 6.88 (d, *J* = 12 Hz, 2H), 3.79 (s, 3H), 3.14 (s, 2H), 2.65 – 2.43 (m, 4H), 1.53 – 1.41 (m, 4H), 1.38 – 1.24 (m, 4H), 0.93 (t, *J* = 7.3 Hz, 6H); ^13^C- NMR (100 MHz, CDCl_3_) δ: 169.74 (C), 156.19 (C), 131.11 (C), 120.79 (CH), 114.22 (CH), 59.27 (CH_2_), 55.50 (CH_3_), 55.28 (CH_2_), 29.54 (CH_2_), 20.61 (CH_2_), 14.01 (CH_3_); IR (KBr): υ: 3278.99, 2997.38, 2954.95, 2862.36 2831.50, 1666.50, 1593.20, 1527.62, 779.24 cm^−1^; HRMS (ESI, m/z): Calcd for C_17_H_28_N_2_O_2_ [M + H]^+^ 293.2229 found 293.2225; HPLC Purity = 98.2% ((Solution A: (TFA 0.01%/acetonitrile) y (Solution B: TFA 0.01%)), 1 ml/min, tR = 3.393 min).

##### 2-(dibutylamine)-*N*-(2,4-dimethoxiphenyl)acetamide (6f)

Yellow liquid; Yield: 84.04%; ^1^H-NMR (400 MHz, CDCl_3_ δ 9.65 (s, 1H), 8.26 (d, *J* = 9.4 Hz, 1H), 6.42 to 6.45 (m, 2H), 3.81 (s, 3H), 3.76 (s, 3H), 3.11 (s, 2H), 2.49 (t, *J* = 7.2 Hz, 4H), 1.52 – 1.40 (m, 4H), 1.34 – 1.23 (m, 4H), 0.88 (t, *J* = 7.3 Hz, 6H); ^13^C- NMR (100 MHz, CDCl_3_) δ: 169.73 (C), 156.29 (C), 149.62 (C), 121.31 (C), 120.26 (CH), 103.68 (CH), 98.67 (CH), 59.82 (CH_2_), 55.56 (CH_3_), 55.49 (CH_3_), 55.15 (CH_2_), 29.67 (CH_2_), 20.39 (CH_2_), 14.05 (CH_3_); IR (KBr): υ: 3313.71, 2870.08, 1685.79, 1600.92, 1527.62, 729.09 cm^−1^; HRMS (ESI, m/z): Calcd for C_18_H_30_N_2_O_3_ [M + H]^+^ 323.2335 found 323.2337; HPLC Purity = 98.4% ((Solution A: (TFA 0.01%/acetonitrilo) y (Solution B: TFA 0.01%)), 1 ml/min, tR = 3.427 min).

##### 2-(dibutylamine)-*N*-(4-methoxiphenyl)propanamide (7a)

Yellow liquid; Yield: 69.42%; ^1^H- NMR (400 MHz, CDCl_3_) δ 9.44 (s, 1H), 7.44 (d, J = 8 Hz, 2H), 6.83 (d, *J* = 8 Hz, 2H), 3.79 (s, 3H), 3.46 (q, *J* = 7,0 Hz, 1H), 2.56 – 2.34 (m, 4H), 1.48 – 1.40 (m, 4H), 1.42 – 1.27 (m, 4H), 1.26 (d, *J* = 4.0 Hz, 3H), 0.93 (t, *J* = 7.3 Hz, 6H); ^13^C- NMR (100 MHz, CDCl_3_) δ: 172.57 (C), 155.95 (C), 131.52 (C), 120.48 (C), 114.20 (C), 60.08 (CH), 55.50 (CH_3_), 50.55 (CH_2_), 30.57 (CH_2_), 20.66 (CH_2_), 14.07 (CH_3_), 8.33 (CH_3_); IR (KBr): υ: 3313.71, 2800 to 3000, 1689.64, 1593.20, 1516.05, 829.39 cm^−1^; HRMS (ESI, m/z): Calcd for C_18_H_30_N_2_O_2_ [M + H]^+^ 307.2386 found 307.2379; HPLC Purity = 99.0% ((Solution A: (TFA 0.01%/acetonitrile) y (Solution B: TFA 0.01%)), 1 ml/min, tR = 3.273 min).

##### 2-(dibutylamine)-*N*-(o-tolyl)propanamide (7b)

Yellow liquid; Yield: 78.22%; ^1^H- NMR (400 MHz, CDCl_3_) δ 9.52 (s, 1H), 8.15 (d, *J* = 7.9 Hz, 1H), 7.22 – 7.11 (m, 2H), 6.99 (t, *J* = 7.4 Hz, 1H), 3.48 (q, *J* = 6.6 Hz, 1H), 2.58 – 2.34 (m, 4H), 2.24 (s, 3H), 1.55 – 1.28 (m, 8H), 1.26 (d, *J* = 6.9 Hz, 3H), 0.89 (t, *J* = 8.0 Hz, 6H); ^13^C- NMR (100 MHz, CDCl_3_) δ: 172.83 (C), 136.45 (C), 130.29 (CH), 126.87 (CH), 126.81 (C), 123.88 (CH), 120.71 (CH), 60.58 (CH), 50.72 (CH_2_), 30.81 (CH_2_), 20.63 (CH_3_), 17.88 (CH_3_), 17.49 (CH_2_), 14.00 (CH_3_); DEPT-135; 130.30 (CH), 126.88 (CH), 123.88 (CH), 120.71 (CH), 60.59 (CH), 50.73 (CH_2_), 30.82 (CH_2_), 20.63 (CH_3_), 17.89 (CH_3_), 17.49 (CH_2_), 14.01 (CH_3_); IR (KBr): υ: 3313.71, 2958.80, 2862.36, 1697.36, 1519.91, 1454.33, 756.10 cm^-1^; HRMS (ESI, m/z): Calcd for C_18_H_30_N_2_O [M + H]^+^ 291.2436 found 291.2425; HPLC Purity = 96.4% ((Solution A: (TFA 0.01%/acetonitrile) y (Solution B: TFA 0.01%)), 1 ml/min, tR = 3.333 min).

##### 2-(dibutylamine)-*N*-(naphthalen-1-yl)propanamide (7c)

Yellow-orange liquid; Yield: 62.84%; ^1^H- NMR (400 MHz, CDCl_3_) δ 10.26 (s, 1H), 8.26 (d, *J* = 7.5 Hz, 1H), 7.87 – 7.82 (m, 2H), 7.61 (d, *J* = 8.2 Hz, 1H), 7.53 – 7.43 (m, 3H), 3.63 (q, *J* = 6.8 Hz, 1H), 2.65 – 2.45 (m, 4H), 1,50 – 1,60 (m, 3H), 1,42 –1,33 (m, 8H), 0.90 (t, *J* = 8 Hz, 6H); ^13^C-NMR (100 MHz, CDCl_3_) δ: 173.07 (C), 134.09 (C), 132.93 (C), 128.88 (CH), 126.09 (CH), 125.95 (CH), 125.89 (CH), 125.77 (CH), 124.33 (CH), 120.11 (CH), 117.74 (CH), 60.76 (CH), 50.85 (CH_2_), 30.74 (CH_2_), 20.69 (CH_2_), 14.22 (CH_3_), 14.01 (CH_3_); DEPT-135; 128.89 (CH), 126.10 (CH), 125.96 (CH), 125.78 (CH), 124.34 (CH), 120.11 (CH), 60.77 (CH), 50.86 (CH_2_), 30.74 (CH_2_), 20.70 (CH_2_), 14.23 (CH_3_), 14.02 (CH_3_); IR (KBr): υ: 3300, 2958, 2870.08, 1697.36, 1527.62, 1492.90, 794.67 cm^-1^; HRMS (ESI, m/z): Calcd for C_21_H_30_N_2_O [M + H]^+^ 327.2436 found 327.2434; HPLC Purity = 100.0% ((Solution A: (TFA 0.01%/acetonitrile) y (Solution B: TFA 0.01%)), 1 ml/min, tR = 3.373 min).

##### 2-(dibutylamine)-*N*-(3,5-dimetilphenyl)propanamide (7d)

Yellow liquid; Yield: 54.92%; ^1^H- NMR (400 MHz, CDCl_3_) δ 9.48 (s, 1H), 7.23 (s, 2H), 6.76 (s, 1H), 3.56 – 3.41 (m, 1H), 2.58 – 2.37 (m, 4H), 2.33 (s, 6H), 1.54 – 1.47 (m, 4H), 1.44 – 1.32 (m, 4H), 1.28 (d, *J* = 7.0 Hz, 3H), 0.96 (t, *J* = 7.3 Hz, 6H); ^13^C- NMR (100 MHz, CDCl_3_) δ: 172.87 (C), 138.71 (C), 137.99 (C), 125.44 (CH), 116.70 (CH), 60.20 (CH), 50.61 (CH_2_), 30.57 (CH_2_), 21.40 (CH_3_), 20.66 (CH_2_), 14.06 (CH_3_), 8.31 (CH_3_); DEPT-135; 125.44, (CH) 116.70 (CH), 60.20 (CH), 50.61 (CH_2_), 30.57 (CH_2_), 21.40 (CH_3_), 20.66 (CH_2_), 14.06 (CH_3_), 8.31 (CH_3_); IR (KBr): υ: 3305.99, 2958.80, 2858.51, 1697.36, 1608.63, 1531.48, 837.11 cm^−1^; HRMS (ESI, m/z): Calcd for C_19_H_32_N_2_O [M + H]^+^ 305.2593 found 305.2594; HPLC Purity = 98.7% ((Solution A: (TFA 0.01%/acetonitrile) y (Solution B: TFA 0.01%)), 1 ml/min, tR = 3.663 min).

### Two-electrode voltage clamp (TEVC) recordings in *X. laevis* oocytes

#### Oocyte preparation and channel cloning

To obtain *X. laevis* oocytes, the frogs were anesthetized with 2 g/L of tricaine methanesulfonate, and the ovarian lobes were mechanically separated with forceps. The oocytes were incubated for 120 min in an OR2 solution containing in mM: NaCl 82.5, KCl 2, MgCl_2_ 1, HEPES 5 (pH 7.5) supplemented with 2 mg/ml of collagenase II (Sigma) to remove residual connective tissue and obtain individual oocytes. Subsequently, the oocytes were washed with a ND96 solution containing in mM: NaCl 96, KCl 2, CaCl_2_ 1.8, MgCl_2_ 1, HEPES 5 (pH 7.5, adjusted with NaOH) and stored at 18 °C in ND96 solution supplemented with 1 ml of gentamicin (50 mg/L), sodium pyruvate (275 mg/L) and (90 mg/L) theophylline (inhibits further oocyte maturation) before and after RNA injection.

In order to overexpress the channel in the oocyte, each of the channels to be measured was subcloned (complementary DNA (cDNA); human TASK-1 (KCNK3, NM_002246), h KV1.5 (KCNA5, NM_002234), h NaV1.5 (hH1, M77235), TASK-4/TALK-2 (KCNK17, NM_AF358910), TREK-1 (KCNK2, AF004711) and Kir2.1 (KCNJ2, NM_017296) into pSGEM, pBF1 or pSP64 expression vector, and the cDNA was linearized with NheI or MluI. Complementary RNA (cRNA) was synthesized with the mMESSAGE mMACHINE-Kit (Ambion). The quality of the cRNA was tested on an agarose gel by electrophoresis. cRNA was quantified using a UV-Vis spectrophotometer (NanoDrop 2000). Subsequently, the channel was transfected in the oocytes in stages V and VI, for which 50 nl (TASK-1, K_V_1.5 or TREK-1), 10 ng (Na_V_1.5 or TASK-4) o 2.5 ng (K_ir_2.1) per oocyte of cRNA was injected with a NanojectII microinjector (Drummond Scientific). The oocytes were stored in 24-well plates for 24 to 48 h at 18 °C. After this time, the overexpression of each channel was monitored through electrophysiology.

#### Two-electrode voltage clamp recordings

Two-electrode voltage clamp measurements were performed 24 or 48 h after cRNA injection at room temperature (20–22 °C) in ND96 recording solution with a TurboTEC-10CD (npi) amplifier and a Digidata 1550B (for TASK-1 and K_V_1.5) and 1440A (for Na_V_1.5) A/D converter from Axon Instruments. Micropipettes were fabricated from GB 150 TF-8P borosilicate glass capillaries (Science Products) elongated in a DMZ-Universal Puller (Zeitz, Germany). Recording pipettes, had a resistance of 0.3 to 0.8 MΩ (for K_V_1.5 and TASK-1) or 0.2 to 1.0 MΩ (for Na_V_1.5), when filled with a 3M KCl solution. ND96 was used as bath solution. Data acquisition was performed using Clampex 10 software (Axon Instruments), and data were analyzed with ClampFit 10 (Axon Instruments) and Origin 7 (Origin Lab Corporation).

For compound inhibition testing, from a holding potential of −80 mV a first test pulse to 0 mV of 1 s duration was followed by a repolarization step at −80 mV for 1 s, directly followed by another 1 s test pulse at +40 mV. The sweep time interval was 10 s for K_V_1.5 and TASK-1 channels. For the Na_V_1.5 channel, the block was analyzed with voltage steps from a holding potential of −80 mV. A first depolarizing pulse to −30 mV of 50 ms duration was applied, followed by a 5 ms step to −80 mV. The sweep time interval was 5 s. The compounds were applied at different concentrations to determine the half-maximal inhibitory concentration (IC_50_), using a fit to the Hill equation.

To test for a putative voltage-dependence of the block by the compound **6f** in K_V_1.5, a 1.5 s voltage step protocol from −80 to +80 mV with +20 mV increments was used. The holding potential was −80 mV and the sweep time interval was 10 s. Currents were analyzed at the end of the voltage pulse. For TASK-1, voltage-dependence of block was probed using a high-potassium solution (KD96) containing in mM: KCl 96, NaCl 2, CaCl_2_ 1.8, MgCl_2_ 1, HEPES 5, (pH 7.5, adjusted with KOH). From a holding potential of 0 mV, voltage was stepped from −80 mV to +80 mV for 1.5 s in +20 mV steps every 10 s. To determine the current-voltage relationship of the Na_V_1.5 channel, the voltage was stepped for 50 ms from a holding potential of −80 mV to −70 mV to +80 mV in +10 mV increments every 5 s. The peak currents were subsequently plotted against the respective applied voltages. The conductance-voltage relationship was calculated by correcting the recorded IV data for the driving force. The data was fitted using the Boltzmann equation. To analyze the frequency-dependence of block, sweep time intervals were changed according to the desired pulse frequency of 1 Hz, 2 Hz or 3 Hz.

#### Preparation of compounds and determination of IC_50_

All compounds were prepared and stored in aliquots dissolved in DMSO on the day of the experiment. The solutions were prepared using ND96 solution as the solvent, according to the required concentration. The mean half-maximum inhibitory concentration (IC_50_) was determined from Hill plots using six concentrations, n = 6 (for TASK-1, K_V_1.5, and Na_V_1.5), and are expressed as mean ± SEM coming from the different replicate measurements (n = 6–15 replicates).

### Patch-clamp recordings in HEK-293 cells

The human embryonic kidney 293 (HEK-293) cell line was obtained from the American Type Culture Collection. HEK-293 cells were cultured in Dulbecco’s modified Eagle’s medium (DMEM-F12, Invitrogen Life Technologies) supplemented with 5% (v/v) fetal bovine serum (FBS) (Thermo Fisher Scientific) and 1% antibiotics (penicillin 100 U/ml y streptomycin 100 μg/ml) (Gibco, Thermo Fisher). The cells were maintained at 37 °C and 5% CO_2_ atmosphere.

#### Transfections

For the electrophysiological experiments, HEK-293 cells were transiently transfected with cDNA encoding human Na_V_1.5 (plasmid 145,374, Addgene), K_V_1.5 (NM_002234), and TASK-1 (NM_002246). Co-transfections of plasmids containing cDNAs of interest and a reporter vector encoding the cDNA for green fluorescent protein (GFP) (1–2 μg of DNA plasmid) were achieved with a 3:1 ratio (channel plasmid: GFP plasmid) using Xfect polymer (Clontech). The cells were incubated for 4 h in transfection medium OptiMEM (Invitrogen). After incubation, the medium was replaced with a fresh culture medium. TASK-1 construct was a kind gift from Dr Steve Goldstein (University of California). Na_V_1.5 construct was acquired from Addgene.

#### Patch-clamp

Whole-cell currents were recorded from HEK-293 cells transiently transfected with the channel-encoding plasmid, at room temperature 24 to 48 h post-transfection using a PC-501A patch-clamp amplifier (Warner Instruments) and borosilicate pipettes as previously described (92). The glass microelectrodes were prepared using horizontal puller (P97 model, Sutter Instruments, Novato, CA, USA) and had a resistance of 1.5–2.5 MΩ. Voltage protocols and data acquisition were controlled using the software WinWCP (Strathclyde University) connected to an acquisition card (DigiData 1440, Molecular Devices, San Jose, CA, USA). For Na_V_1.5, the cells were continuously perfused with bath solution containing (in mM): 140 NaCl, 5 KCl, 10 HEPES, 1 MgCl_2_, 1.8 CaCl_2_, and 10 glucose, adjusted to pH 7.4 using NaOH. For K_V_1.5 and TASK-1, the solution contained (in mM) 135 NaCl, 5 KCl, 1 MgCl_2_, 1 CaCl_2_, 10 HEPES, and 10 Sucrose, adjusted to pH 7.4 with NaOH, and pH 8.0 to TASK-1. For the Na_V_1.5 experiments, the pipettes were filled with an internal solution of (in mM) 130 CsF, 20 CsCl, 10 HEPES, and 5 EGTA, adjusted to pH 7.4 with CsOH. The intracellular pipette solution to K_V_1.5 and TASK-1 contained (in mM): 145 KCl, 5 EGTA, 2 MgCl_2_, and 10 HEPES, adjusted to pH 7.4 with KOH. All recordings were done at room temperature (22–24 °C).

#### Protocols and drug preparation

A stock of compound **6f** was prepared in dimethyl sulfoxide (DMSO) (ChemCruz) and stored. Working concentrations of compound **6f** were then prepared daily, in an external bath solution to a final concentration of 10 μM and 100 μM for patch-clamp recordings and the final DMSO concentration did not exceed 0.1%. For TASK-1 only, the drug was specifically dissolved in the bath solution at pH 8.0.

For Nav1.5, the effect of **6f** at both concentrations was tested using a repetitive single test voltage pulse at −10 mV (during 20 ms) from a holding potential of −80 mV, with intervals of 5s between pulses. For Kv1.5 and TASK-1, ramp voltage protocols were applied with pulse intervals of 20 s, and the currents were analyzed at +40 mV.

In particular, the Kv1.5 ramp protocol consists of a voltage increase to +40 mV during 400 ms from a holding potential of −80 mV. This is followed by a ramp in which the voltage decreases until it reaches the holding potential of −80 mV. Finally, a +40 mV pulse is applied for 100 ms. For TASK-1, the voltage protocol begins at −40 mV. The voltage is then increased in a stepwise manner, with increments of +20 mV, reaching a maximum potential of +60 mV. Each voltage step lasts for 80 ms. After reaching the peak voltage, a descending ramp is applied to return to the holding potential of −80 mV. Finally, a pulse of −40 mV is applied immediately after the ramp.

After recording currents with stable amplitudes, the cells were treated with **6f** and applied from the bath solution. Currents were continuously recorded during repetitive pulses until a stable effect was achieved. This protocol allows the recording of currents before (*Icontrol*) and after drug application (*Idrug*) to assess the extent of drug activity. The inhibition percentage was calculated as follows: *In*ℎ*ibition* (%) = 100 ∗ (*Icontrol* − *Idrug*)/*Icontrol*.

### Statistical analysis for TEVC and patch-clamp recordings in HEK-293 cells

To analyze statistically the TEVC results, the system (protein) and the drugs were selected as factors. The independent and combined effects of the factors on the response variable (inhibition percentage) were evaluated using generalized linear models (GLMs) and Heteroscedastic tests. Statistical analysis of Figures 5, 6, 8 and S1, was performed using R software version 4.3.2 (93). It was verified if the data followed a normal distribution (94), then it was verified if variances were homogeneous (95), and according to this result, GLM with Gamma or Gaussian distribution using identity as the link function (Figs. 5, 6, and S1) (96) was used. To evaluate the effect of the factors on the data behavior was performed ANOVA (97) in every GLM, and according to the results, multiple comparisons were performed using a *post hoc* analysis with the Tukey test. For Figure 8, Welch's Heteroscedastic F test (97) was employed, and multiple comparisons were performed using the Bonferroni test (98, 99). The significance level (alpha) for all tests was 0.05, and the significance codes are shown as follows: ∗∗∗*p* < 0.001, ∗∗*p* < 0.01, ∗*p* < 0.05.

### Cardiomyocyte assays

#### Ethics statement

A total of 10 patients with sinus rhythm (SR) (N = 5) or atrial fibrillation (AF) (N = 5) undergoing open heart surgery for coronary artery bypass grafting, heart valve replacement or heart transplantation were included in the study (Table S7). A written informed consent was given by all patients. The study protocol involving human tissue samples was approved by the responsible Ethics Committee of the Medical Faculty of Heidelberg University (Germany; S-017/2013) and was conducted in accordance with the 1964 Declaration of Helsinki.

#### Cardiomyocyte isolation

The right atrial human tissue samples were transported in chilled Ca^2+^-free solution (100 mM NaCl, 10 mM KCl, 1.2 mM KH_2_PO_4_, 5 mM MgSO_4_, 50 mM taurine, 5 mM 3-(*N*-morpholino)propanesulfonic acid (MOPS), 30 mM 2,3-butanedione monoxime and 20 mM glucose, pH 7.0 with NaOH). After dissection into small chunks, the samples were rinsed for 5 minutes with calcium-free Tyrode's solution, which was oxygenated with 100% O_2_ at 37 °C. The samples were then digested with collagenase type I (288 U/ml; Worthington) and protease type XXIV (5 mg/ml; Sigma-Aldrich, Steinheim, Germany) for 10 to 15 min, followed by an increase in calcium concentration to 0.2 mM. After an additional 35 min of agitation in protease-free solution, rod-shaped single cardiomyocytes were harvested. The suspension was centrifuged, and the cells were resuspended in storage (“Kraftbrühe”, KB) medium (20 mM KCl, 10 mM KH_2_PO_4_, 25 mM glucose, 40 mM D-Mannitol, 70 mM K glutamate, 10 mM β-hydroxybutyrate, 20 mM taurine, 10 mM ethylene glycol tetraacetic acid (EGTA), and 1% albumin) until usage in patch-clamp experiments.

#### Patch-clamp electrophysiology

Patch-clamp glass pipettes were fabricated from borosilicate glass (1B120F-4; World Precision Instruments). After back-filling with patch-clamp internal solution (134 mM K gluconate, 6 mM NaCl, 1.2 mM MgCl_2_, 1 mM Mg-ATP, 10 mM HEPES, pH 7.2 with KOH), tip resistances ranged from 5 to 10 MΩ. All experiments were carried out at room temperature under constant superfusion with patch-clamp extracellular solution containing 137 mM NaCl, 5.4 mM KCl, 1 mM MgCl_2_, 2 mM CaCl_2_, 10 mM HEPES, 10 mM glucose (pH 7.3 with NaOH). AP-recordings were performed in current clamp mode: The holding current was adjusted such that the cell’s resting membrane potential was approximately −70 mV at baseline. APs were induced by short (5 ms) depolarizing current pulses at a rate of 0.5 Hz. Data was not adjusted for potential differences caused by liquid junctions, and no leak subtraction was performed.

#### Statistical analysis

Data acquisition and analysis were performed using the pCLAMP10 software (Axon Instruments) and statistical analysis was done using Prism 8 (GraphPad Software). Data is presented as the mean ± standard error of the mean (SEM). Statistical comparisons were made using Dunnett’s post hoc test. A *p* value of < 0.05 was considered significant.

### *In silico* trials for the pharmacological management of AF with compound 6f

The efficacy of compound **6f** for both acute AF cardioversion and long-term AF prevention was assessed in a population of 45 virtual-atria human models with variability in ionic current channel densities. The *in silico* cohort presented structurally-healthy atria (*i.e.*, absence of tissue abnormalities such as fibrosis) and normal chamber dimensions (right and left atrial volume of 93 ml and 109 ml, respectively), to replicate early stages of AF where pharmacological cardioversion is more often indicated (100). Each of the 45 virtual atria had a different ionic current profile, since we have previously demonstrated that variability in ionic current density is the main determinant of response to drug treatment (74, 75).

#### Population of atrial cardiomyocyte models

To capture human variability in ionic currents, a population of atrial cardiomyocyte models was generated. The population was constructed with a modified version of the CRN model (101) as in Wiedmann *et al.*, 2020 (102), to include the K_2P_ channel family. The family comprises the Tandem of P domains as a weak inward rectifying K^+^ channel (TWIK)-related acid-sensitive K^+^ channel (TASK-1, K_2P_3.1; Wiedmann *et al.*, 2020). To account for variability, the ionic current densities of the modified CRN model were varied up to ± 50% (103). This variation included the ultrarapid and rapid delayed-rectifier K^+^ current (G_Kur_, G_Kr_), transient outward (G_to_) and inward rectifier K^+^ current (G_K1_), L-type Ca^2+^ current (G_CaL_), fast Na^+^ current (G_Na_), Na^+^/K^+^ pump (G_NaK_), and the K_2P_ current (G_K2P_) densities. A hundred myocyte models, generated with Latin Hypercube sampling, were calibrated against experimental data obtained from patients in sinus rhythm and AF (104). A calibrated sample of 45 atrial cardiomyocyte models was used to populate a whole-atria model.

#### Populations of virtual-atria models

Whole-atria simulations were performed using a mean bi-atrial model derived from averaging the atrial anatomy of 47 human subjects (105). A population of 45 virtual-atria models was generated by assigning the single-cell properties of each atrial cardiomyocyte model to the left atrial tissue and scaling them by reported regional differences in ion channel expression to the right atrium, crista terminalis, pectinate muscles, left atrial appendage, and atrioventricular rings (106, 107). Regional heterogeneities in conduction velocity and anisotropy ratio were likewise considered, setting the longitudinal velocity in the bulk tissue to 80 cm/s.

#### AF inducibility

AF was induced in the virtual-atria models by imposing spiral wave re-entries as the initial conditions of the simulation. AF dynamics were then analyzed for 7 s of activity (108), since we showed in a similar virtual population (75) that all AF episodes sustaining over 7 s would also sustain for 30 s (*i.e.*, duration used clinically for AF diagnosis). The three-dimensional monodomain equation of the transmembrane voltage was solved using the MonoAlg3D software (109).

#### *In silico* drug trials

Sustained (>7 s) AF episodes were subjected to the virtual administration of three concentrations (*i.e.*, 1, 10, and 100 μM) of compound **6f**. Efficacy for acute AF cardioversion was investigated by applying each concentration 2 s after AF induction, and the episode was recorded for another 5 s. AF was considered successfully cardioverted by the drug if the atria were free of arrhythmic activity no later than 7 s after AF induction (108).

Long-term AF prevention was modeled by repeating the AF initiation protocol after drug application (106). Drug action was simulated using simple pore-block models based on the 50% inhibitory concentration and Hill coefficient profiles. The ionic current block of each concentration according to the inhibitory curves presented in Figure 7 is summarized in Table 5.

**Table 5:** Corresponding ionic current block based on the inhibition curves for *I*_K2P_ (TASK-1), *I*_Kur_ (K_V_1.5) and I_Na_ (Na_V_1.5)

Drug	Concentration	Ionic current block (%)		
		*I*_Kur_	*I*_Na_	*I*_K2P_
Compound **6f**	1 μM	0	16	58
	10 μM	2.5	30	75
	100 μM	50	65	85

### Determination of ADME properties

The SwissADME website, a freely available online tool (66), was utilized to evaluate the ADME properties (absorption, distribution, metabolism, and excretion) of the synthesized compounds and LA. This involved the calculation of gastrointestinal absorption, pharmacokinetic descriptors, and physicochemical descriptors, including the assessment of compliance with the Lipinski's Rule of Five (67, 110, 111).

### Cytotoxicity assays

To evaluate cytotoxicity, the hemolysis of the compounds with the best simultaneous blocking activity on the channels (**6e, 6f, 7a, 7b** and **7c**) was considered, modifying the protocol of Chen 2018 (112). Thus, non-heparinized human blood was collected. Erythrocytes were washed three times in 1× PBS (phosphate-buffered saline: NaCl, 8 g/L; KCl, 0.2 g/L; Na_2_HPO_4_, 1.44 g/L; and KH_2_PO_4_, 0.24 g/L; pH 7.4) and then used to prepare a 3% red blood cell (RBC) suspension. The assay was performed in a 96-well polypropylene microplate.

The compounds were prepared from 200 mM stock solutions in DMSO. Subsequently, 1:2 serial dilutions were made in 1× PBS, with final concentrations from 400 to 0.2 μM to a final volume of 200 μl per well. The microplate was incubated for 2 h at 37 °C and centrifuged at 3400 rpm for 5 min. Finally, 100 μl of the supernatant was transferred and measured in a 96-well plate. Hemoglobin release was monitored by photometric analysis of the supernatant at 540 nm using an Epoch Microplate Spectrophotometer (BioTek Instruments, Inc. Winooski, Vermont 05404-0998).

A 3% RBC suspension treated with Tween 20 was used to obtain total lysis as a positive control. Other authors who performed hemolysis assays have also considered this and other detergents, such as Triton X-100 and Sodium Dodecyl Sulfate (SDS) as positive controls (113, 114, 115, 116). The negative control was a suspension of red blood cells with PBS. Each experiment was performed in triplicate at all concentrations used. The percentage of hemolysis was calculated with the following (Equation 1), where Ad: absorbance of the dilution, A0: absorbance of the blank (RBC suspension with PBS), and At: total absorbance (positive control).

(1) $$\%\;\text{hemolysis}=\frac{Ad-A0}{At-A0}x\;100$$

Another method used to assess cytotoxicity was the MTT assay on HEK293 cells. Initially, the cells were seeded at 3000 cells per well in a 96-well plate. After 24 h, they were exposed to increasing compound concentrations (1, 5, 10, 25, 50, 100, 250 and 500 μM) for 72 h. The cells cultured in a medium supplemented with 10% FBS were the positive control for cell viability. Each condition was evaluated triplicate in independent experiments. The ROCHE Cell Proliferation Kit I, MTT (Cat. no. 11465007001), was used, following the manufacturer's indications. Briefly, 10 μl of the labeling reagent was added, and the plates were incubated at 37 °C for 4 h. Subsequently, 100 μl of solubilization solution was added to each well, and the plates were incubated overnight in the dark. Finally, optical density was measured at 570 nm using a microplate reader (Thermo Fisher Scientific). The optical density measurements were used to calculate cell viability, normalizing the data to the positive control. Results were expressed as a percentage of cell viability relative to this control. The IC_50_ value was determined by fitting a dose-response curve using a nonlinear regression model with a four-parameter logistic equation. The experimental data analysis was performed using SigmaPlot version 12.0 (Systat Software Inc).

## Data availability

Data supporting the original contributions presented in the study are included in the article and supplementary materials.

## Supporting information

This article contains supporting information.

## Conflict of interest

The authors declare that they have no conflicts of interest with the contents of this article.

## Supporting information

Supplemmentary Material
