# Supplementary material for: Rational design, synthesis, and evaluation of novel polypharmacological compounds targeting NaV1.5, KV1.5, and K2P channels for atrial fibrillation
Source: J Biol Chem. 2025 Mar 5;301(4):108387. doi: 10.1016/j.jbc.2025.108387 (PMC12131194; doi:10.1016/j.jbc.2025.108387)
Supplement: Supplemmentary Material [file mmc1.docx]

Rational Design, Synthesis, and Evaluation of Novel Polypharmacological Compounds Targeting Na_V_1.5, K_V_1.5, and K_2_P Channels for Atrial Fibrillation

Lorena Camargo-Ayala^a,†^, Mauricio Bedoya^b,c,†^, Albert Dasí^d†^, Merten Prüser^e,f,g†^, Sven Schütte^h^, Luis Prent-Peñaloza^i^, Francisco Adasme-Carreño^b,c^, Aytug K Kiper^h,j^, Susanne Rinné^h^, Paola Andrea Camargo-Ayala^k^, Paula A Peña-Martínez^l,m^, Alfonso Bueno-Orovio^d^, Diego Varela^n,o^, Felix Wiedmann^e,f,g^, José C. E. Márquez-Montesinos^p,q^, Yuliet Mazola^p,q^, Whitney Venturini^r^, Rafael Zúñiga^q^, Leandro Zúñiga^q^, Constanze Schmidt^e,f,g*^, Blanca Rodriguez^d*^, Ursula Ravens^s,t*^, Niels Decher^h^*, Margarita Gutiérrez^u*^, and Wendy González^n,p*^

^a^ Doctorado en Ciencias Mención I + D de Productos Bioactivos, Instituto de Química de Recursos Naturales, Laboratorio de Síntesis Orgánica, Universidad de Talca, Casilla 747, Talca 3460000, Chile.

^b^ Centro de Investigación de Estudios Avanzados del Maule (CIEAM), Vicerrectoría de Investigación y Postgrado, Universidad Católica del Maule, Talca 3480112, Chile.

^c^ Laboratorio de Bioinformática y Química Computacional (LBQC), Departamento de Medicina Traslacional, Facultad de Medicina, Universidad Católica del Maule, Talca 3480112, Chile.

^d^ Department of Computer Science, British Heart Foundation Centre of Research Excellence, University of Oxford, Oxford, UK.

^e^ Department of Cardiology, University of Heidelberg, Heidelberg, Germany.

^f^ DZHK (German Center for Cardiovascular Research), partner site Heidelberg /Mannheim, University of Heidelberg, Heidelberg, Germany.

^g^ HCR, Heidelberg Center for Heart Rhythm Disorders, University of Heidelberg, Heidelberg, Germany.

^h^ Institute for Physiology and Pathophysiology, Philipps-University Marburg, Marburg, Germany.

^i^ Departamento de Ciencias Químicas, Facultad de Ciencias Exactas, Universidad Andrés Bello, Quillota 980, Viña del Mar, Chile.

^j^ Institute of Physiology, University Medicine Greifswald, 17475, Greifswald, Germany.

^k^ Doctorado en Ciencias Biomédicas, Laboratorio de Patología Molecular, Departamento de Ciencias Básicas Biomédicas, Facultad de Ciencias de la Salud, Universidad de Talca, Talca 3460000, Chile.

^l^ Doctorado en Ciencias Agrarias, Facultad de Ciencias Agrarias, Universidad de Talca, Talca, Chile.

^m^ Laboratorio de Química Enológica, Facultad de Ciencias Agrarias, Universidad de Talca, Talca, Chile

^n^ Millennium Nucleus of Ion Channels-Associated Diseases (MiNICAD), Santiago, Chile.

^o^ Program of Physiology and Biophysics, Institute of Biomedical Sciences, Faculty of Medicine, Universidad de Chile, Santiago, Chile.

^p^ Centro de Bioinformática, Simulación y Modelado (CBSM), Universidad de Talca, Casilla 747, Talca 3460000, Chile.

^q^ Centro de Nanomedicina, Diagnóstico y Desarrollo de Fármacos (ND3), Laboratorio de Fisiología Molecular, Escuela de Medicina, Universidad de Talca, Casilla, Talca 3460000, Chile.

^r^ Departamento de Medicina Traslacional, Facultad de Medicina, Universidad Católica del Maule.

^s^ German Atrial Fibrillation Competence NETwork (AFNET), Germany.

^t^ Institute of Experimental Cardiovascular Medicine, University Heart Center Freiburg – Bad Krozingen, Medical Center – University of Freiburg and Faculty of Medicine, Freiburg, Germany.

^u^ Laboratorio Síntesis Orgánica y Actividad Biológica (LSO-Act-Bio), Instituto de Química de Recursos Naturales, Universidad de Talca, Casilla 747, Talca 3460000, Chile.

*Corresponding author:

Constanze Schmidt, [constanze.schmidt@med.uni-heidelberg.de](mailto:constanze.schmidt@med.uni-heidelberg.de)

Blanca Rodriguez, [blanca.rodriguez@cs.ox.ac.uk](mailto:blanca.rodriguez@cs.ox.ac.uk)

Ursula Ravens, [ursula.ravens@uniklinik-freiburg.de](mailto:ursula.ravens@uniklinik-freiburg.de)

Niels Decher, [decher@staff.uni-marburg.de](mailto:decher@staff.uni-marburg.de)

Margarita Gutiérrez, [mgutierrez@utalca.cl](mailto:mgutierrez@utalca.cl)

Wendy González, [wgonzalez@utalca.cl](mailto:wgonzalez@utalca.cl)


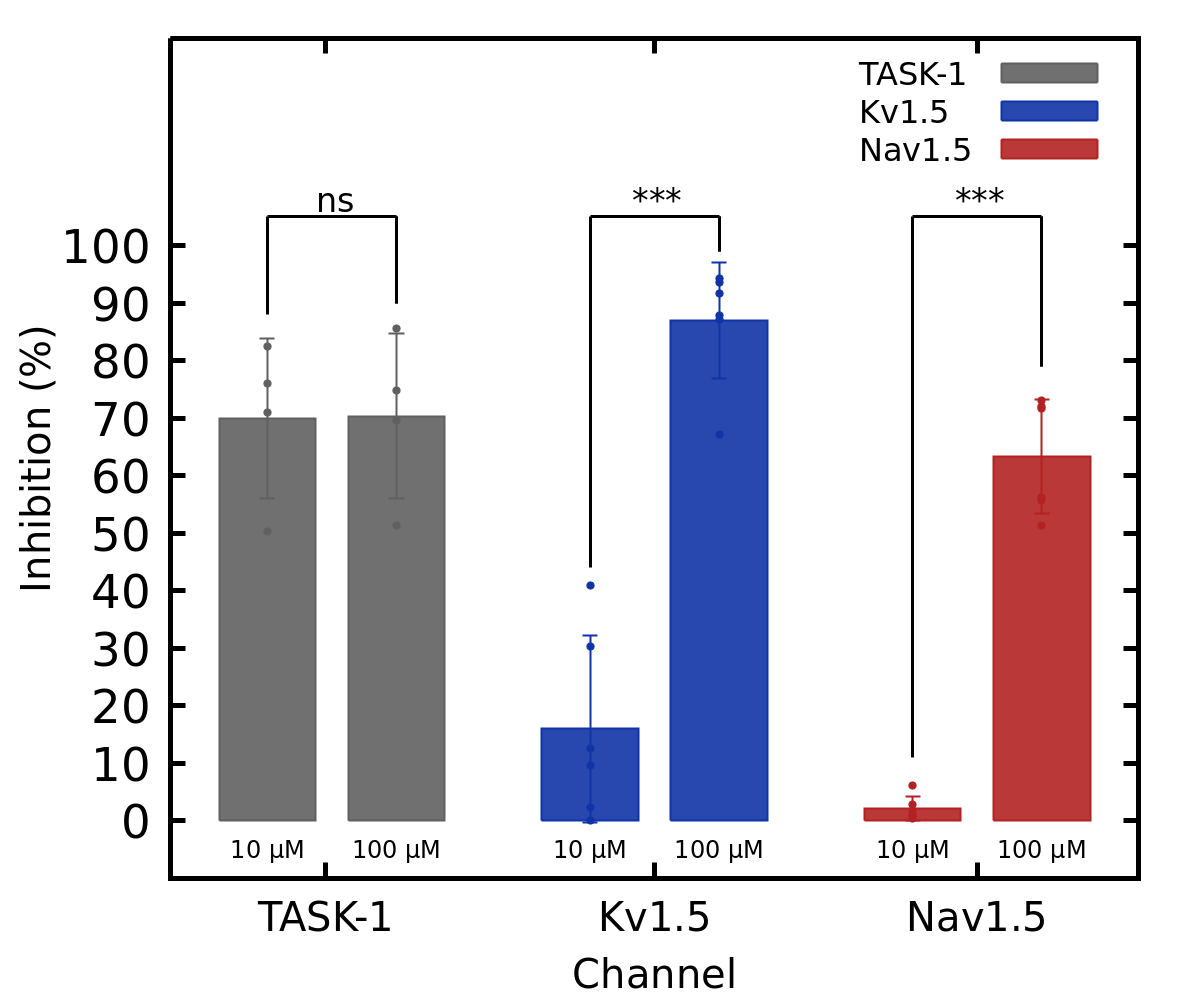


Figure S1. The inhibitory activity of compound 6f for human TASK-1, human Kv1.5, and human Nav1.5 channels transiently expressing in HEK293 cells. Current recordings before and after application of 6f, at concentrations of 10 µM and 100 µM, were done using patch-clamp whole-cell configuration. For TASK-1 (n=4) and Kv1.5 (n=6) inhibition was evaluated at +40 mV and, at -10 mV for Nav1.5 (n=6). Data are represented as mean ± SD (n: number of cells used per experimental condition). Statistical significance was defined as follows: n.s. (p > 0.05), * (p < 0.05), ** (p < 0.01) and *** (p < 0.001).


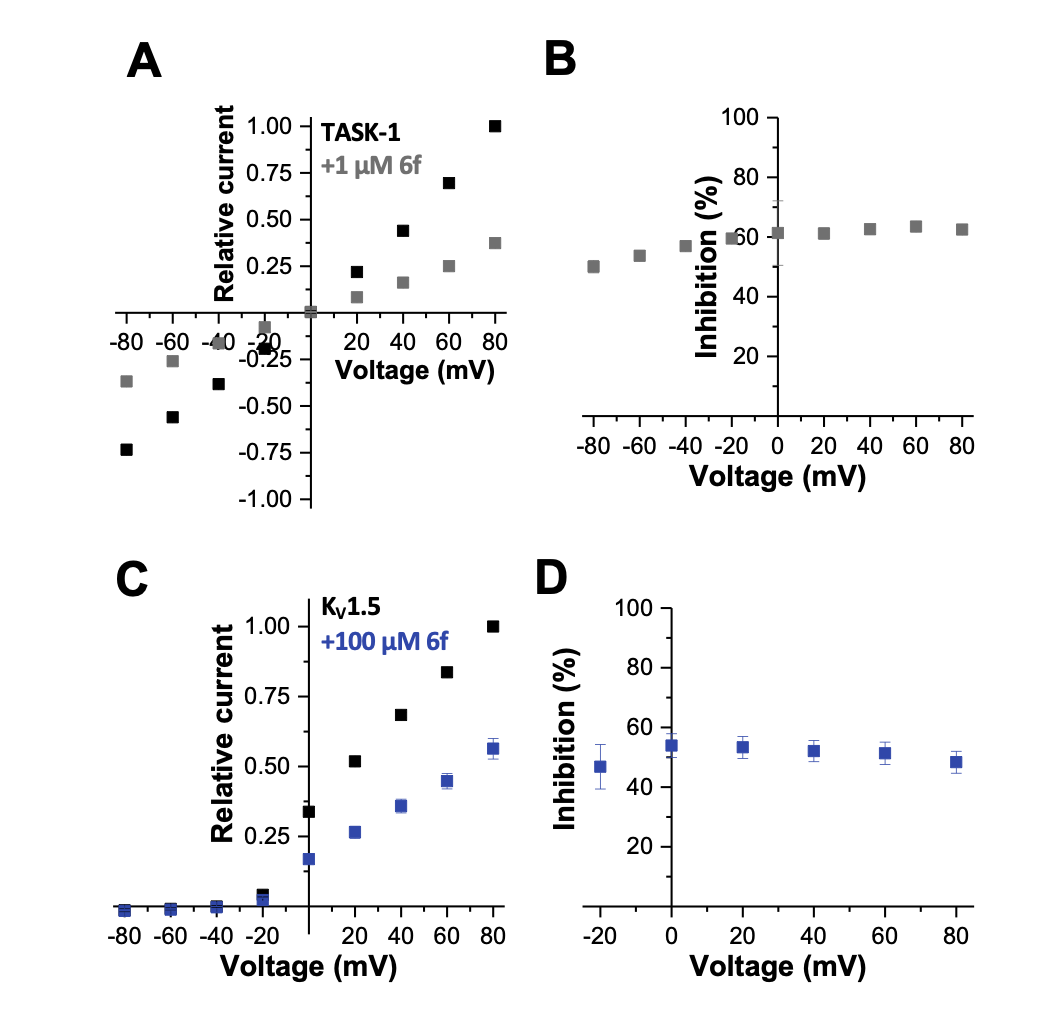


**Figure S2.** (A) Current-voltage-relationship of TASK-1 before and after application of 1 µM **6f** (gray), measured in KD96 solution. (B) Voltage-dependence of block (%) of TASK-1 currents by 100 µM **6f**. n = 6. (C) Current-voltage-relationship of the K_V_1.5 channel before and after application of 100 µM **6f** (blue). (D) Voltage-dependence of block (%) of K_V_1.5 currents by 100 µM **6f**. n = 9. For TASK-1 and K_V_1.5, significance of voltage-dependent changes in block was probed against the data at 0 mV.


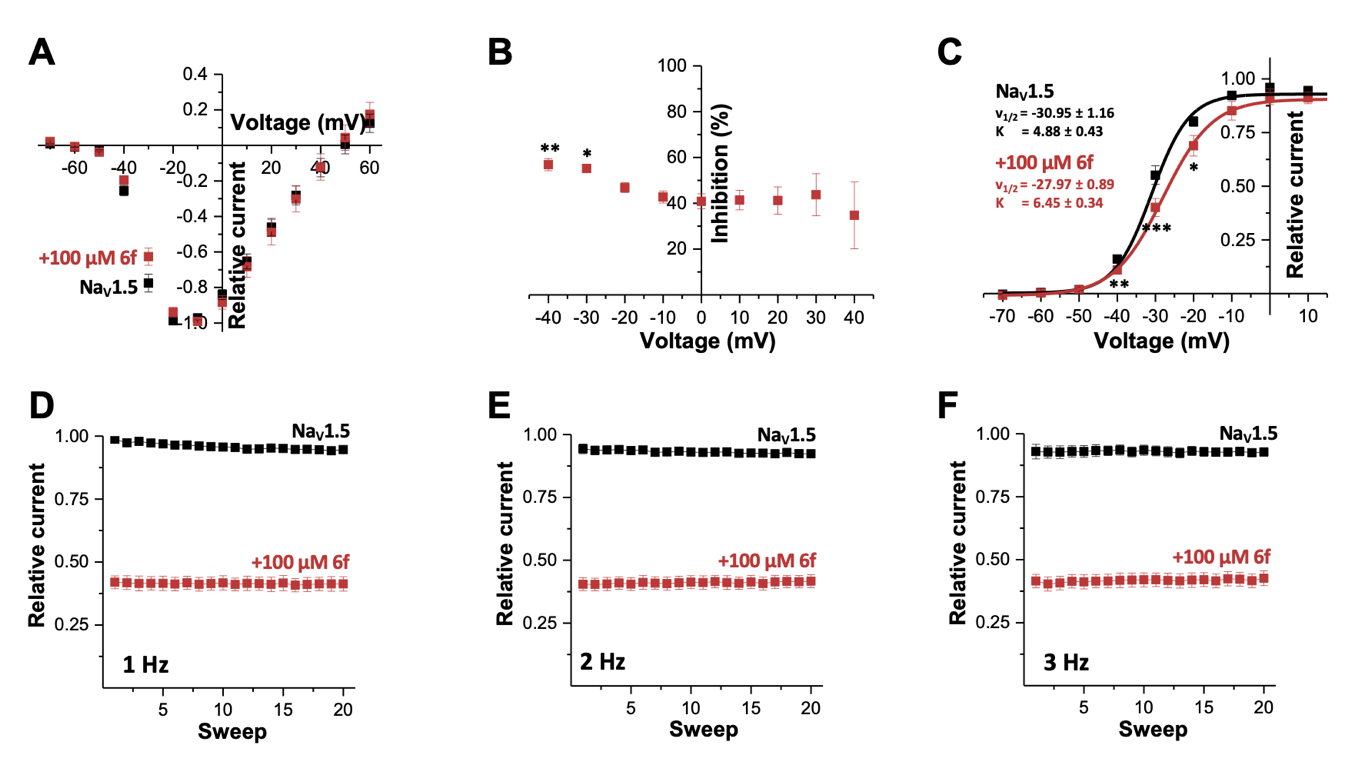


**Figure S3.** (A) Relative current-voltage relationship of the Na_V_1.5 channel before (black) and after (red) application of 100 µM **6f**. (B) Analysis of the percentage of block of Na_V_1.5 currents by 100 µM **6f** in dependence of the applied voltage. Significance of voltage-dependent changes in block was probed against the data at 0 mV. (C) Conductance-voltage relationships (GVs) of the Na_V_1.5 channel before (black) and after (red) application of 100 µM **6f**. The GVs were determined by correcting the peak currents by their respective driving force. (D-F) Frequency-dependent analyses of Na_V_1.5 currents (1, 2 and 3 Hz) before and after application of 100 µM **6f**. n = 8.


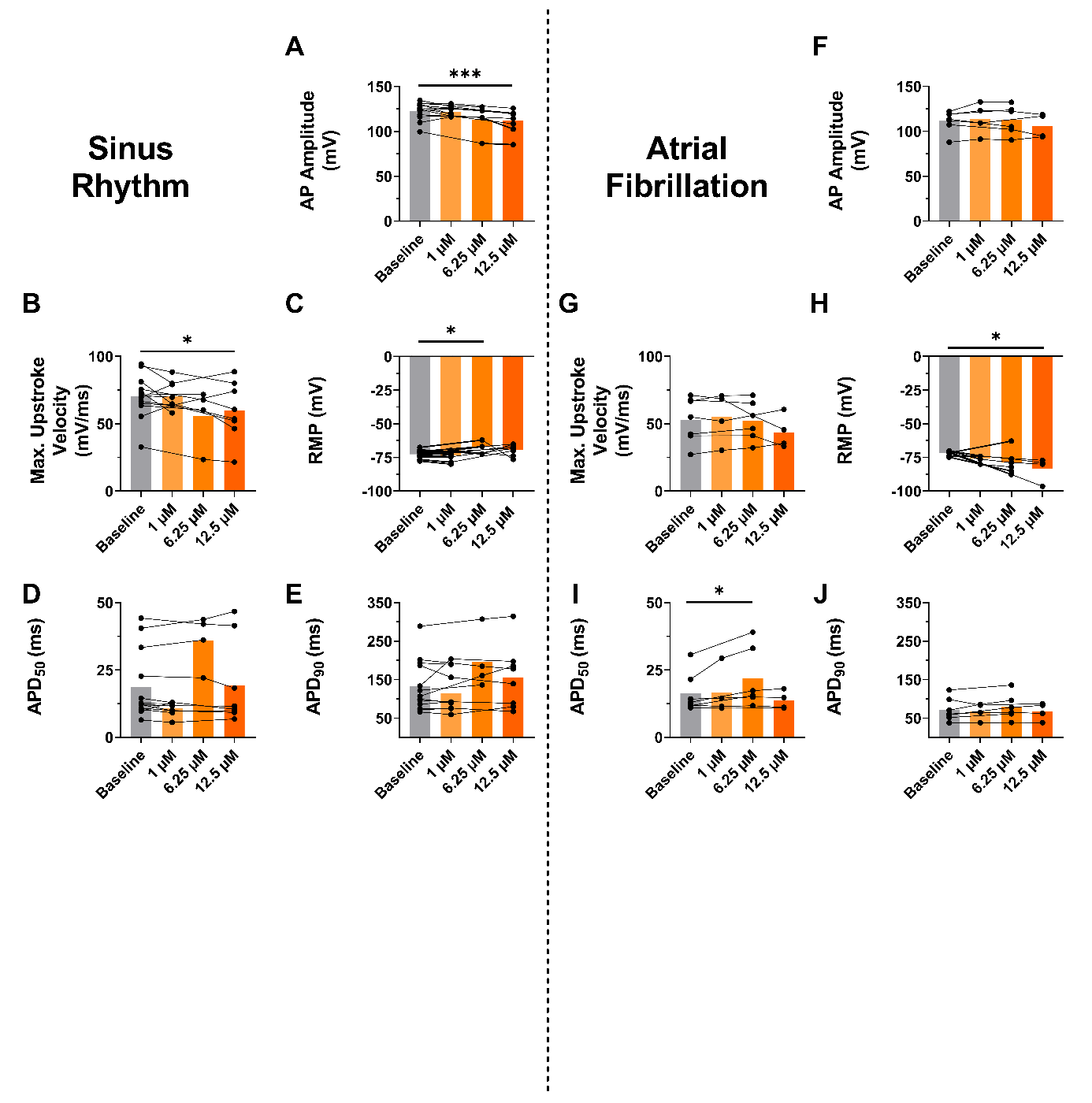


**Figure S4**: (A – E) Atrial AP recordings from SR patient samples (n=13 cells/N=5 patients). Recorded absolute values for AP amplitude, maximum upstroke velocity, resting membrane potential, APD50 and APD90 at baseline and after application of compound 6f at concentrations of 1 µM, 6.25 µM and 12.5 µM. (F – J) AP recordings from AF patient samples (n=7 cells/N=5 patients). Recorded absolute values for AP amplitude, maximum upstroke velocity, resting membrane potential, APD50 and APD90 at baseline and after application of compound 6f at concentrations of 1 µM, 6.25 µM and 12.5 µM. Recordings from the same cell are connected by black lines. Individual columns were compared to baseline using a mixed effects model with Dunnett’s post-hoc-test. *indicates a p-value of <0.05 and *** a p-value of <0.001.


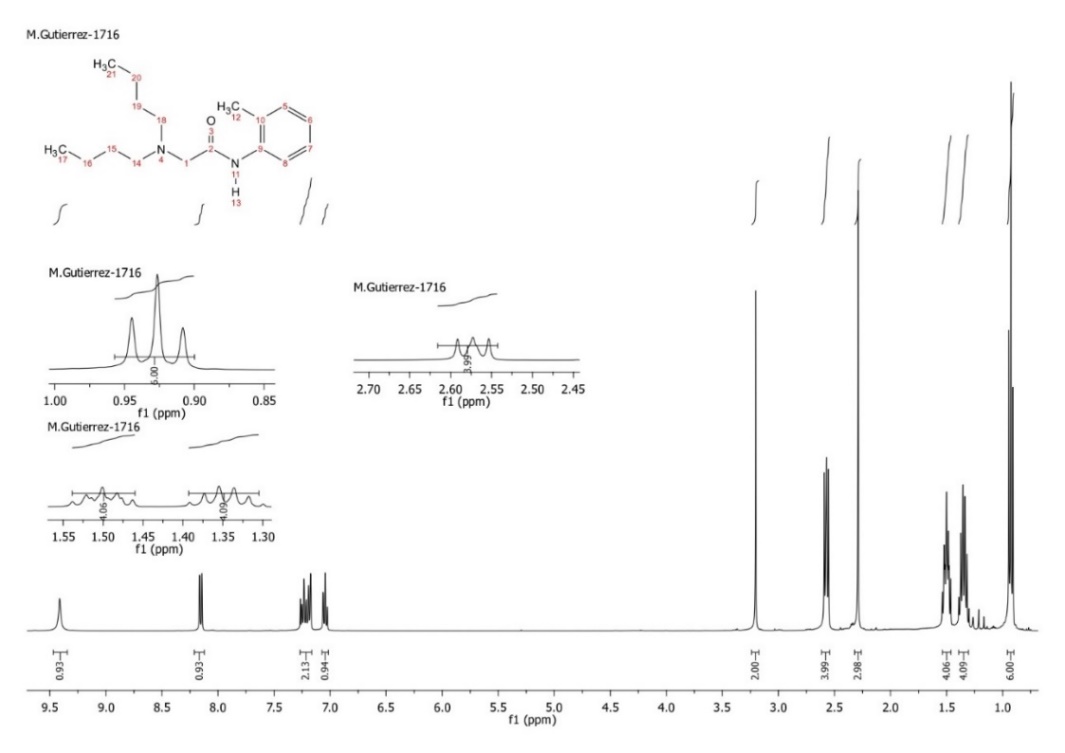


Figure S5. ^1^H-RMN (400 MHz, CDCl_3_) of 2-(dibutylamino)-*N*-(o-tolyl)acetamide (6a)


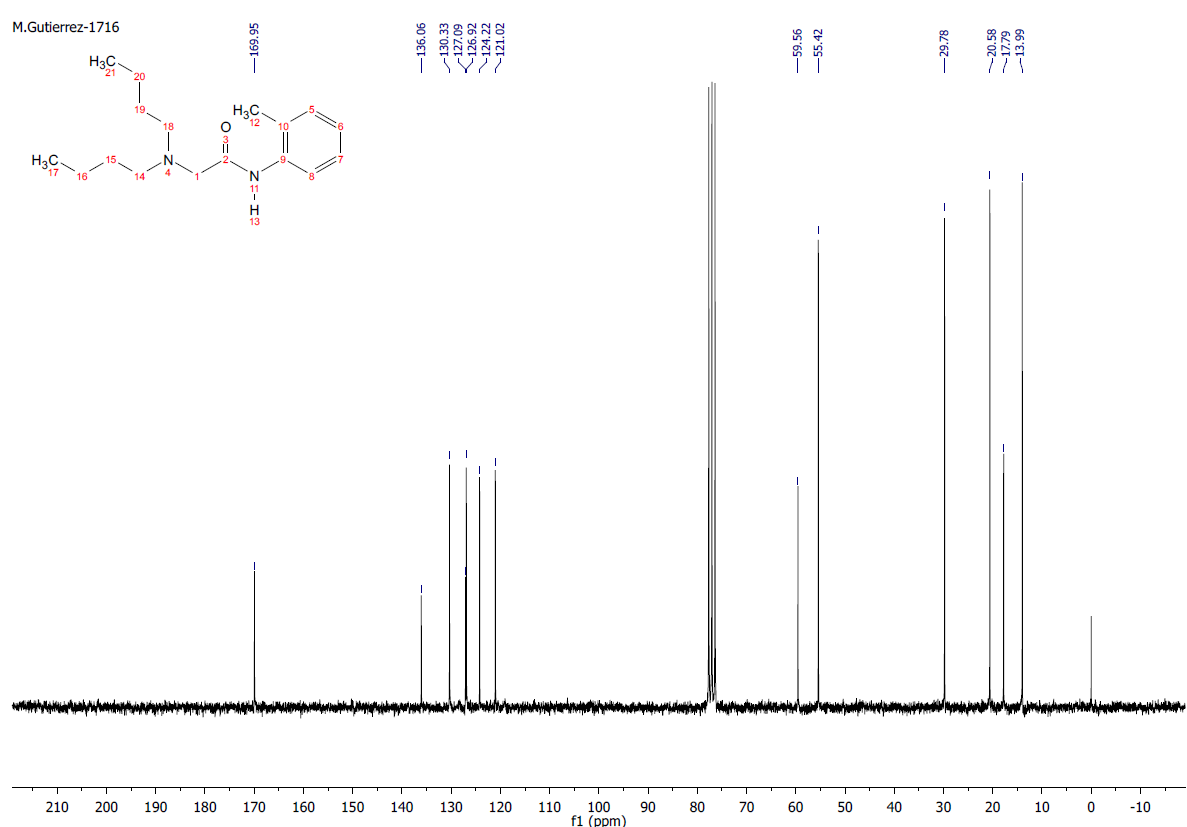


Figure S6. ^13^C-RMN (100 MHz, CDCl_3_) of 2-(dibutylamino)-*N*-(o-tolyl)acetamide (6a)


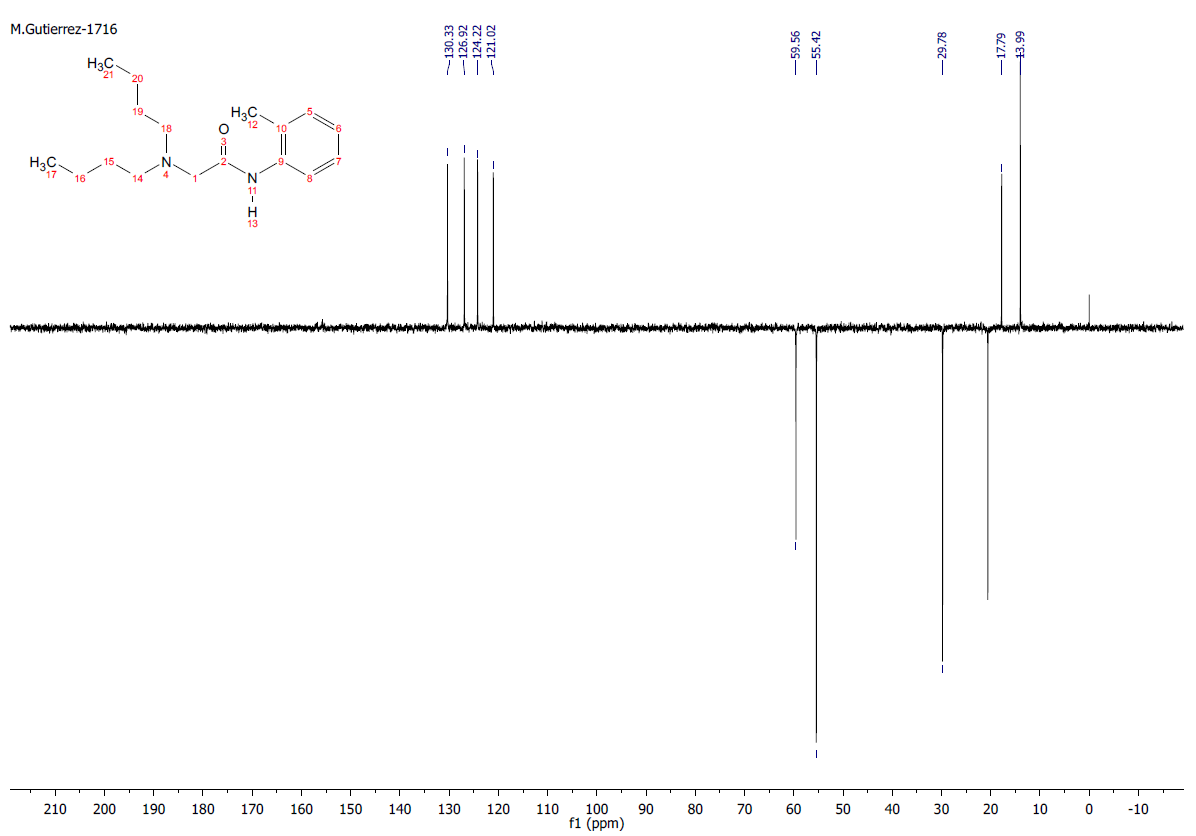


Figure S7. DEPT-135 (CDCl_3_) of 2-(dibutylamino)-*N*-(o-tolyl)acetamide (6a)


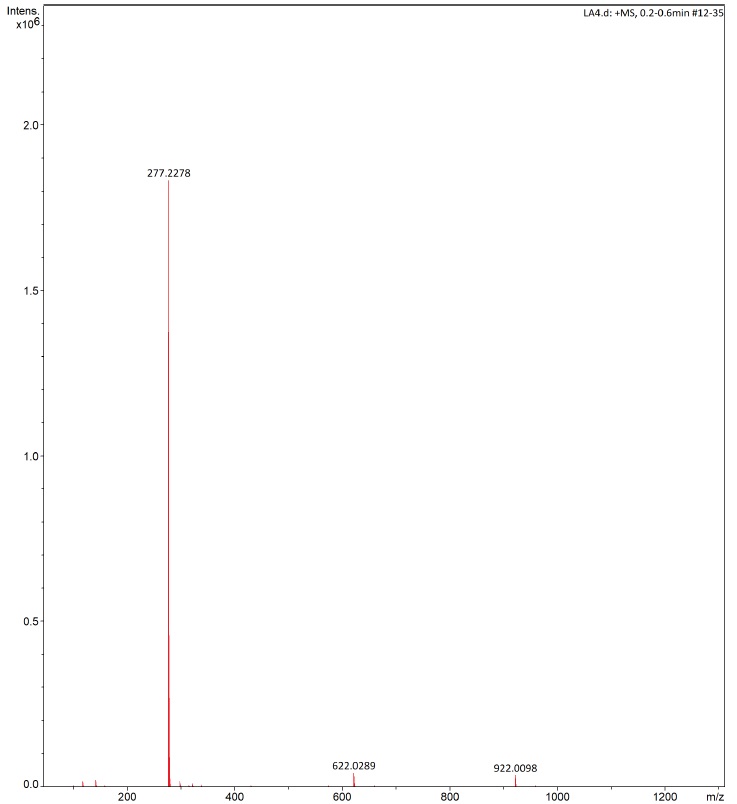


Figure S8. HRMS (ESI, m/z) of 2-(dibutylamino)-*N*-(o-tolyl)acetamide (6a)

**
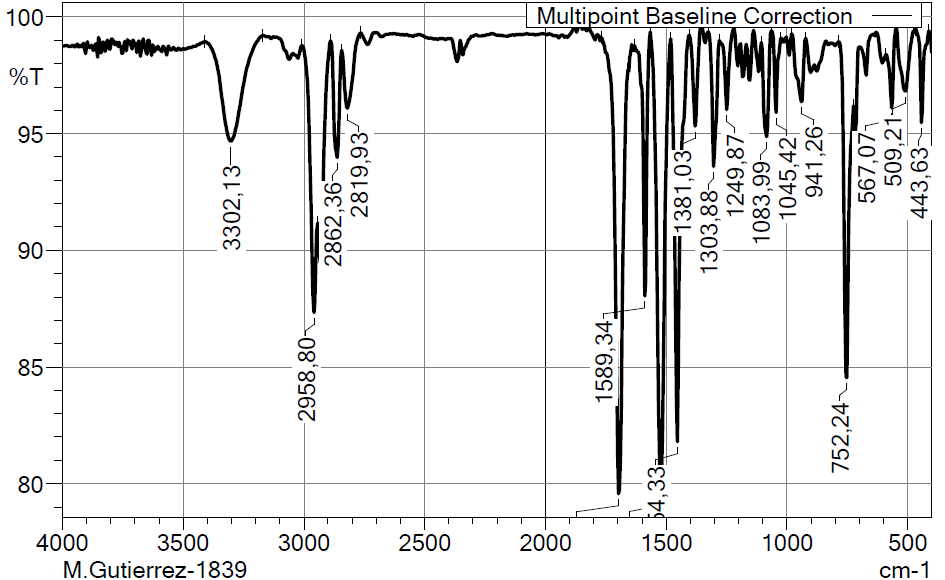
**

Figure S9. IR of 2-(dibutylamino)-*N*-(o-tolyl)acetamide (6a)


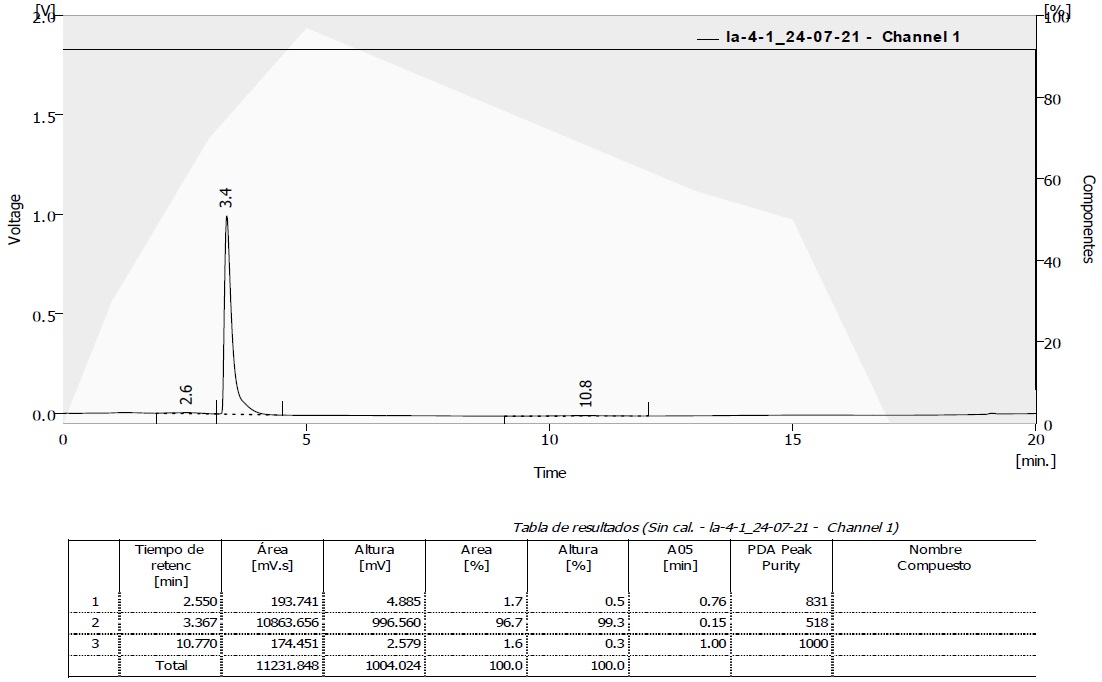


Figure S10. HPLC of 2-(dibutylamino)-*N*-(o-tolyl)acetamide (6a)


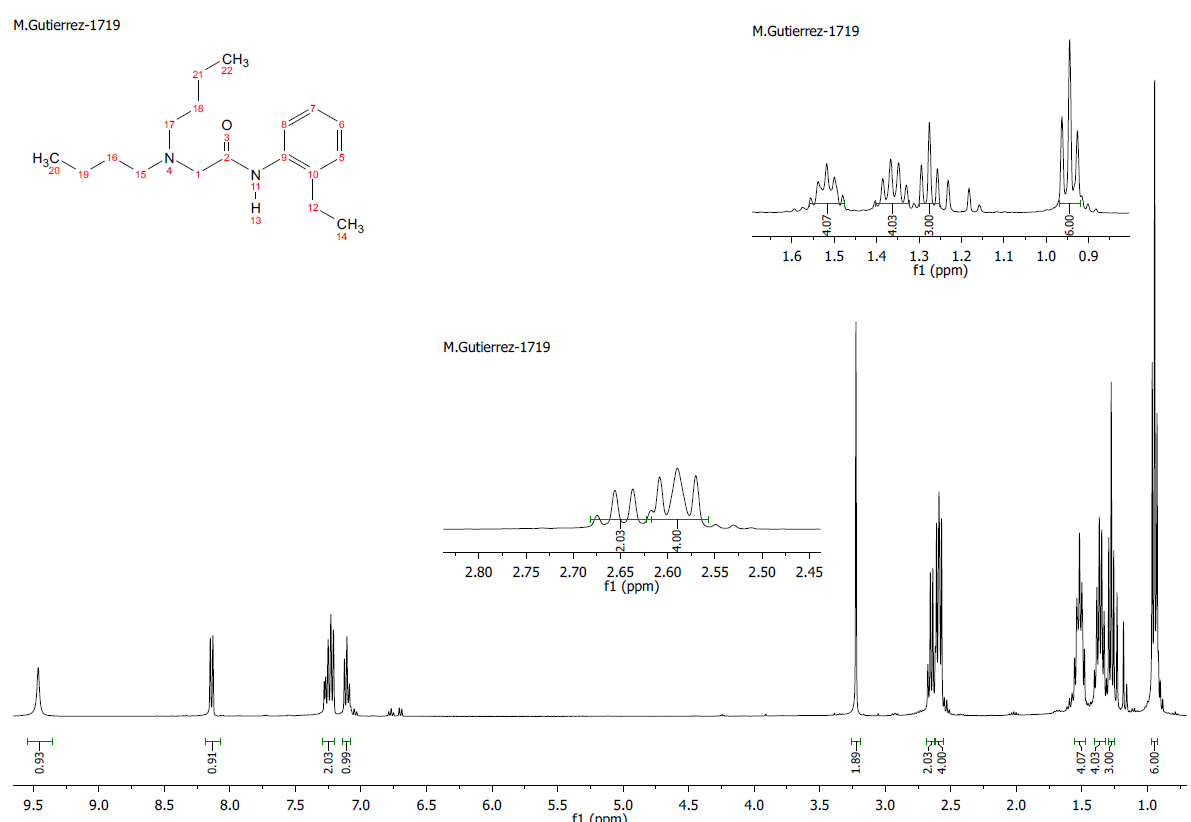


Figure S11. ^1^H-RMN (400 MHz, CDCl_3_) of 2-(dibutylamino)-*N*-(2-ethylphenyl)acetamide (6b)


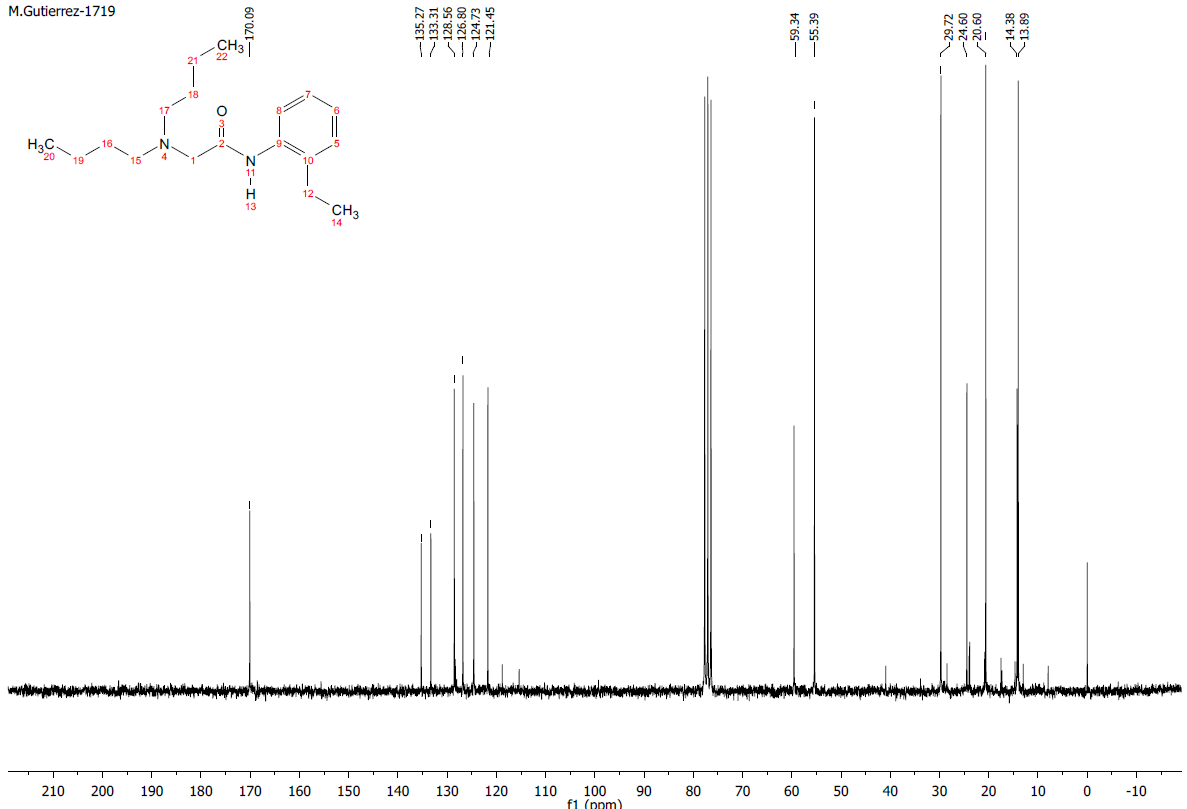


Figure S12. ^13^C-RMN (100 MHz, CDCl_3_) of 2-(dibutylamino)-*N*-(2-ethylphenyl)acetamide (6b)


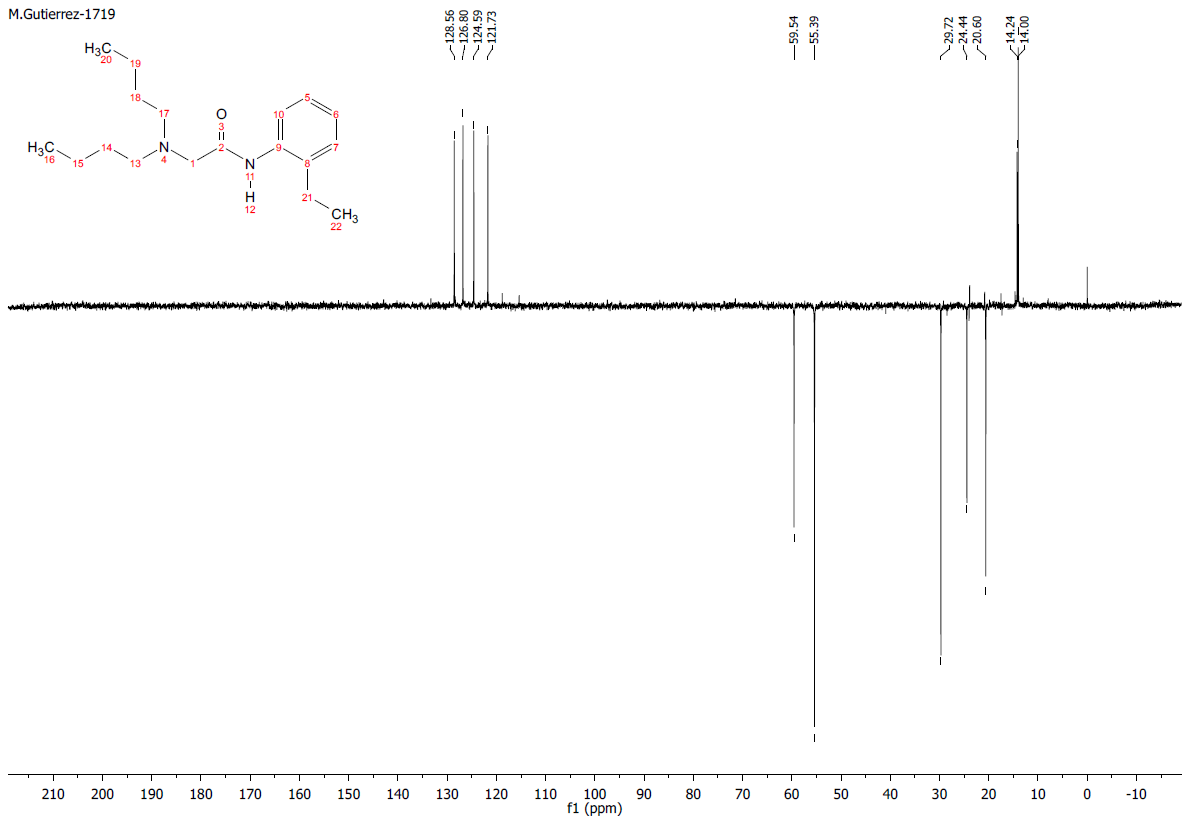


Figure S13. DEPT-135 (CDCl_3_) of 2-(dibutylamino)-*N*-(2-ethylphenyl)acetamide (6b)


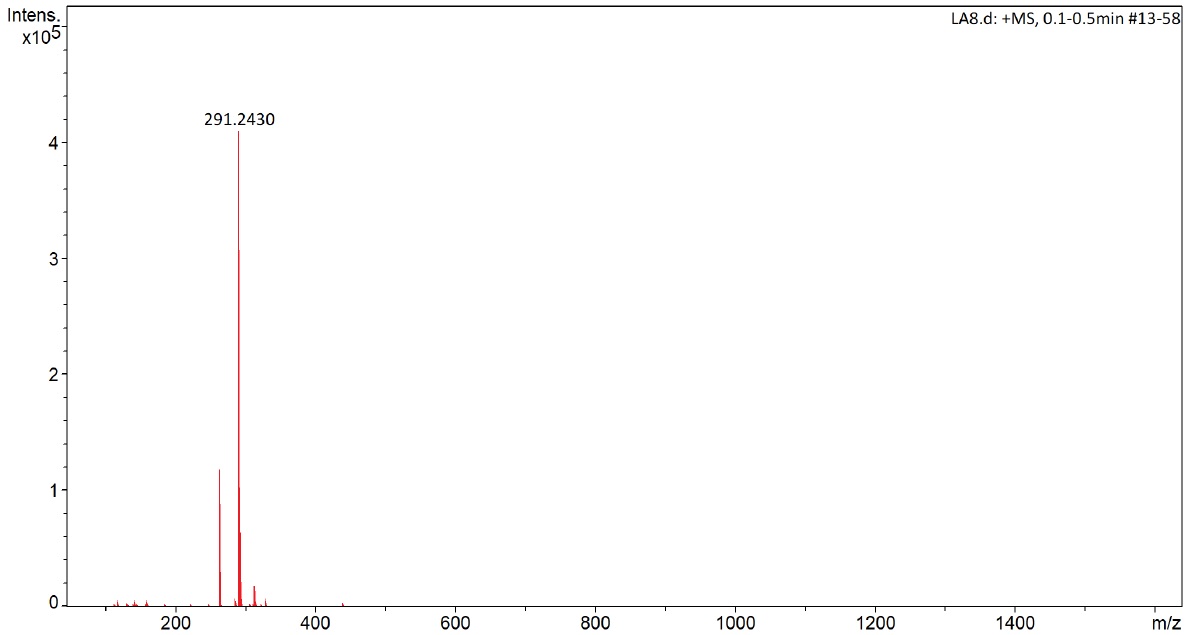


Figure S14. HRMS (ESI, m/z) of 2-(dibutylamino)-*N*-(2-ethylphenyl)acetamide (6b)

**
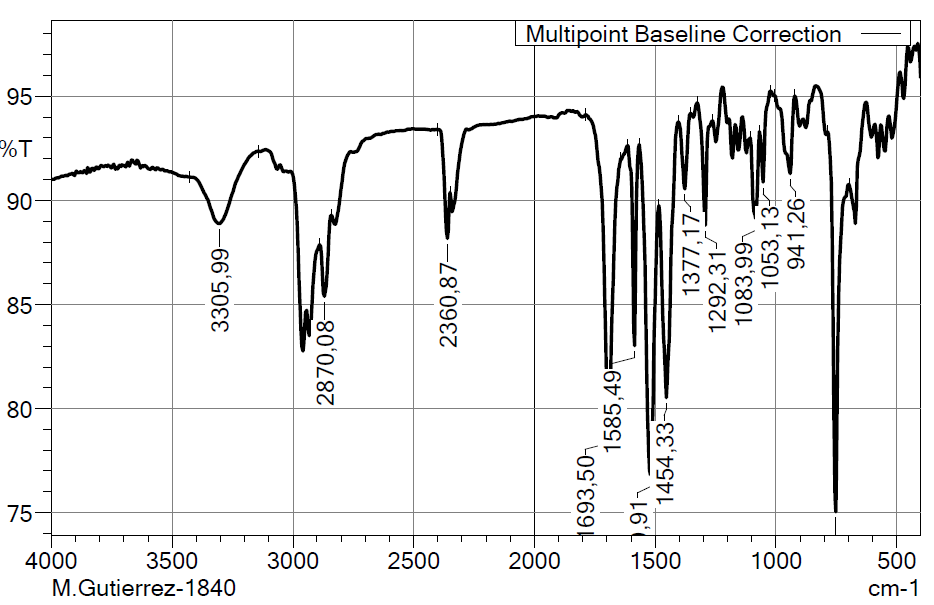
**

Figure S15. IR of 2-(dibutylamino)-*N*-(2-ethylphenyl)acetamide (6b)


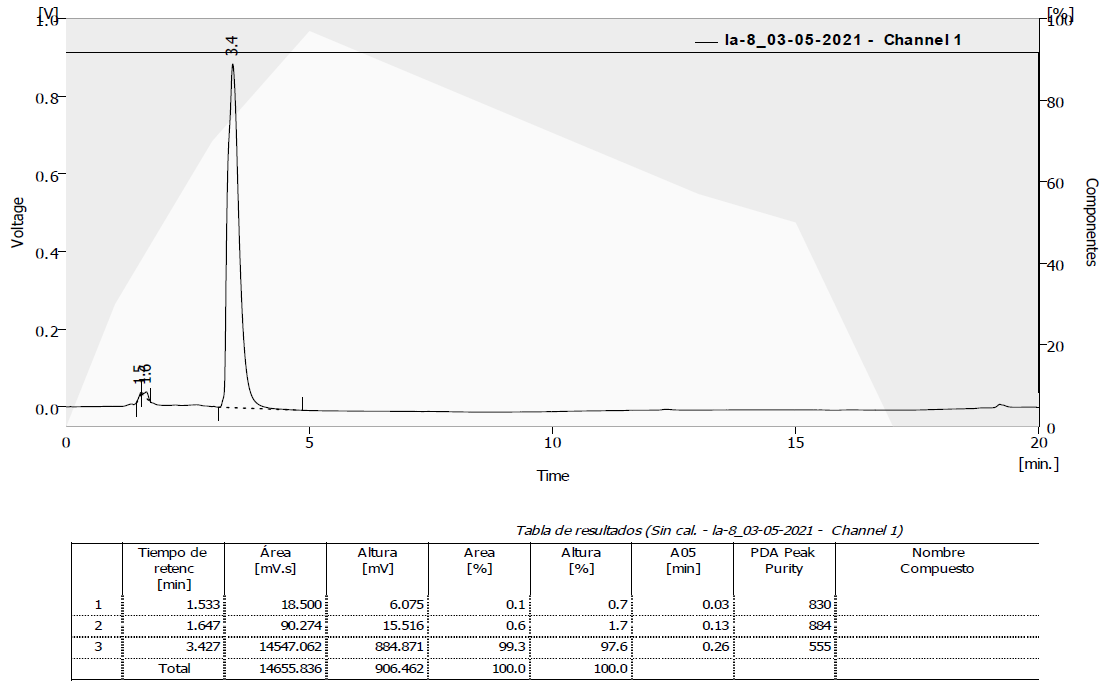


Figure S16. HPLC of of 2-(dibutylamino)-*N*-(2-ethylphenyl)acetamide (6b)


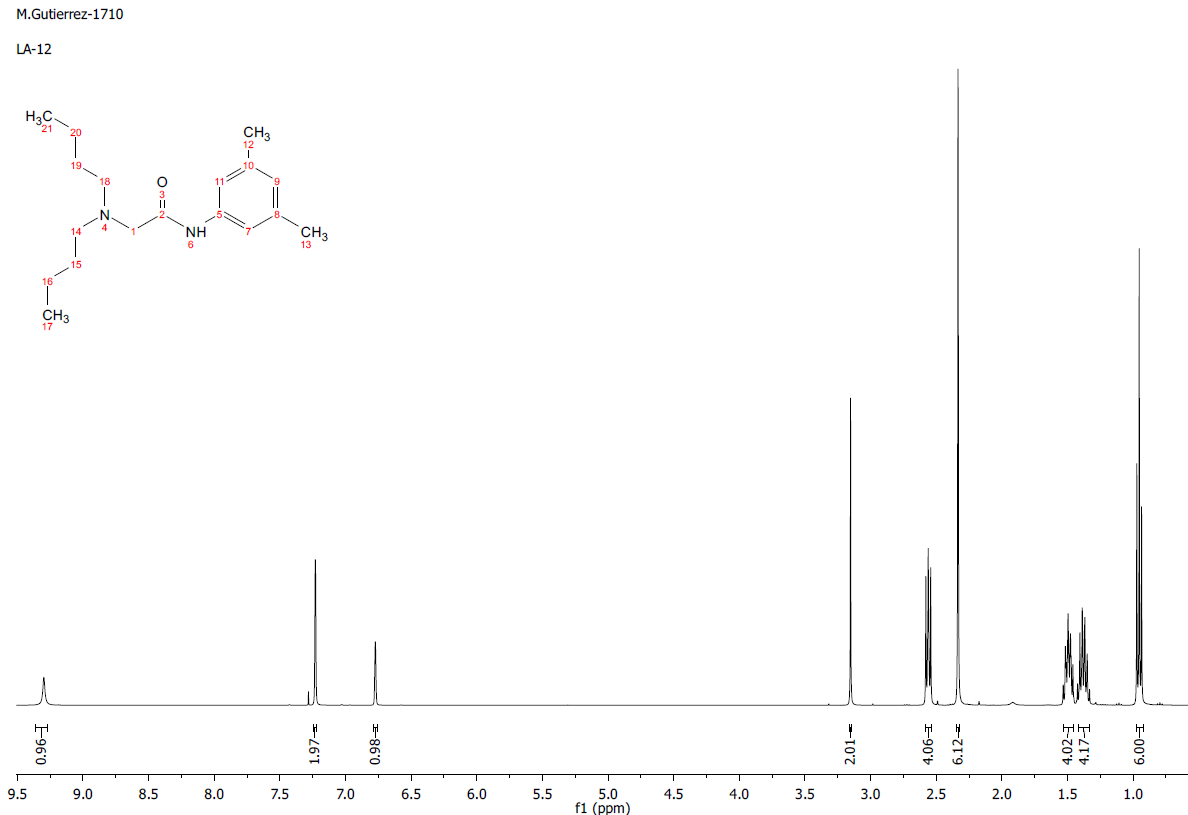


Figure S17. ^1^H-RMN (400 MHz, CDCl_3_) of 2-(dibutylamino)-*N*-(3,5-dimethylphenyl)acetamide (6c)


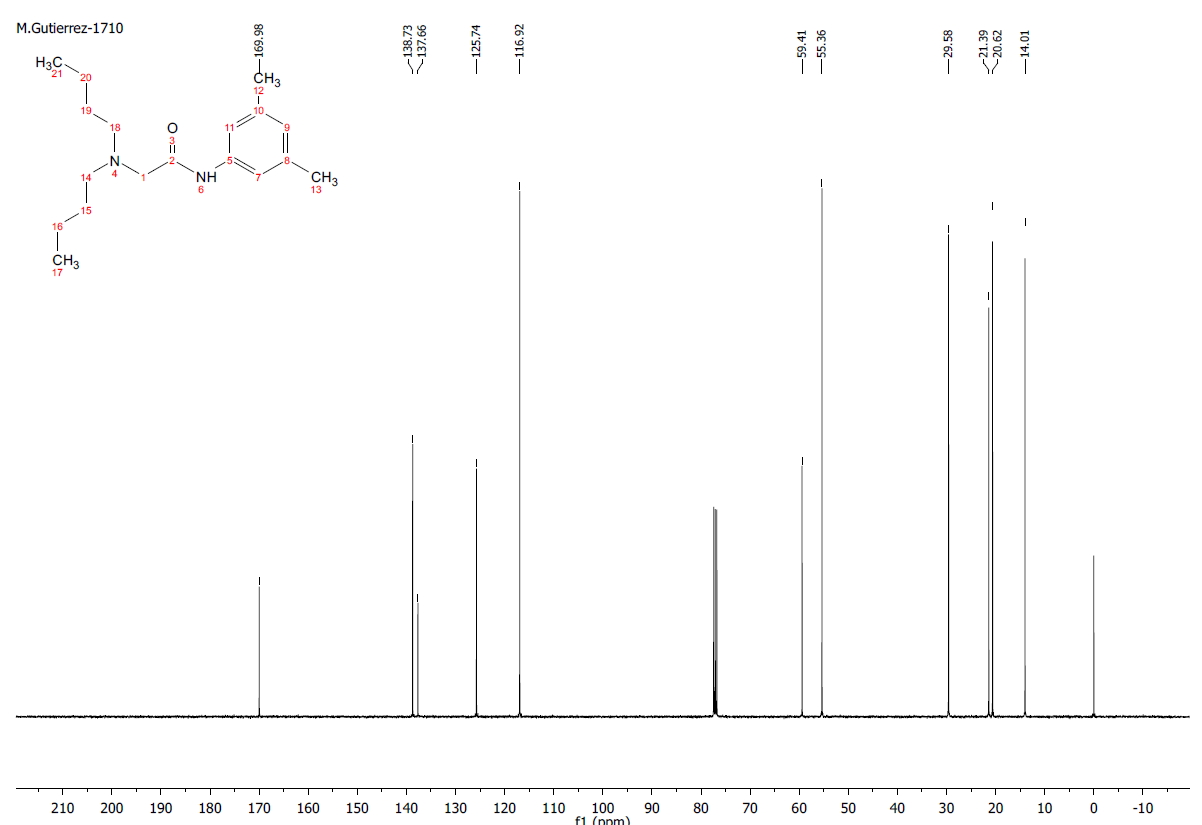


Figure S18. ^13^C-RMN (100 MHz, CDCl_3_) of 2-(dibutylamino)-*N*-(3,5-dimethylphenyl)acetamide (6c)


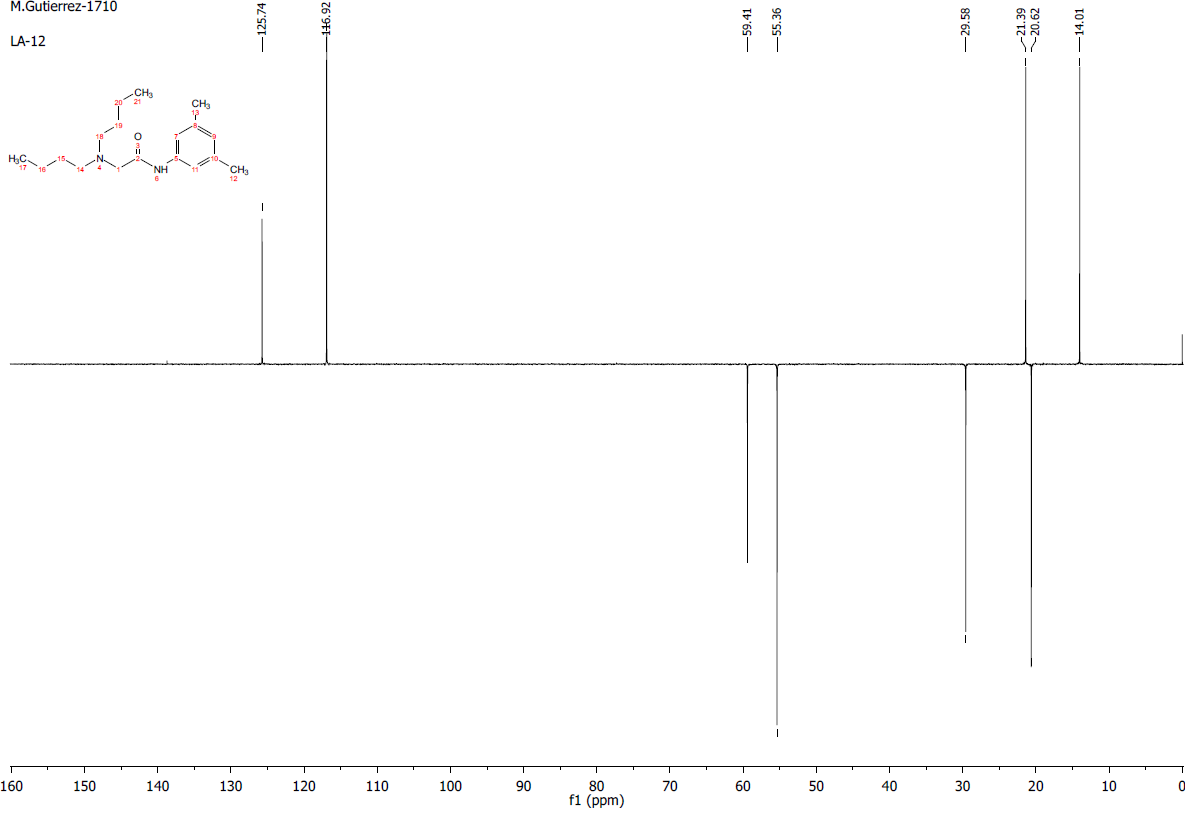


Figure S19. DEPT-135 (CDCl_3_) of 2-(dibutylamino)-*N*-(3,5-dimethylphenyl)acetamide (6c)


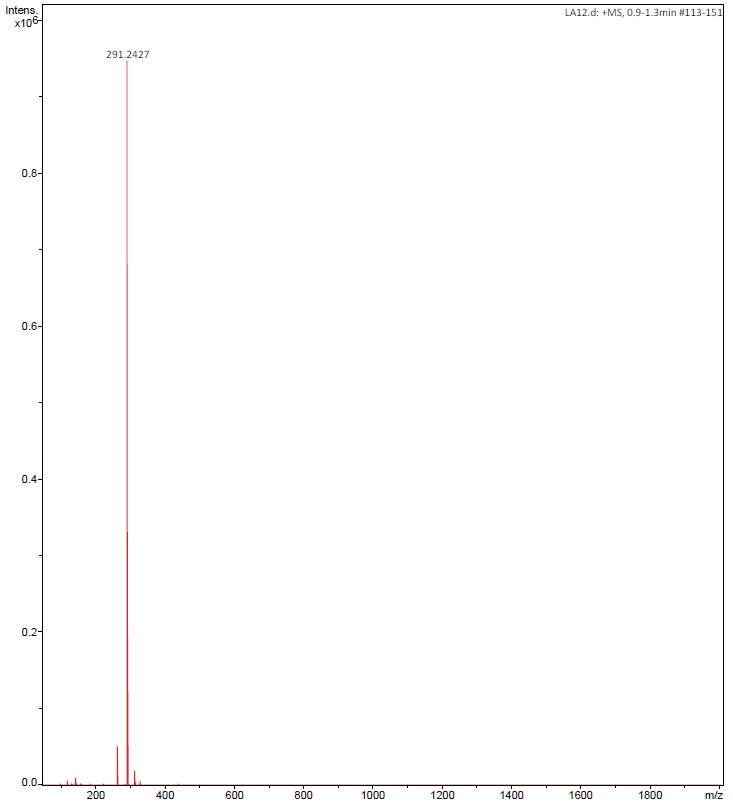


Figure S20. HRMS (ESI, m/z) of 2-(dibutylamino)-*N*-(3,5-dimethylphenyl)acetamide (6c)

**
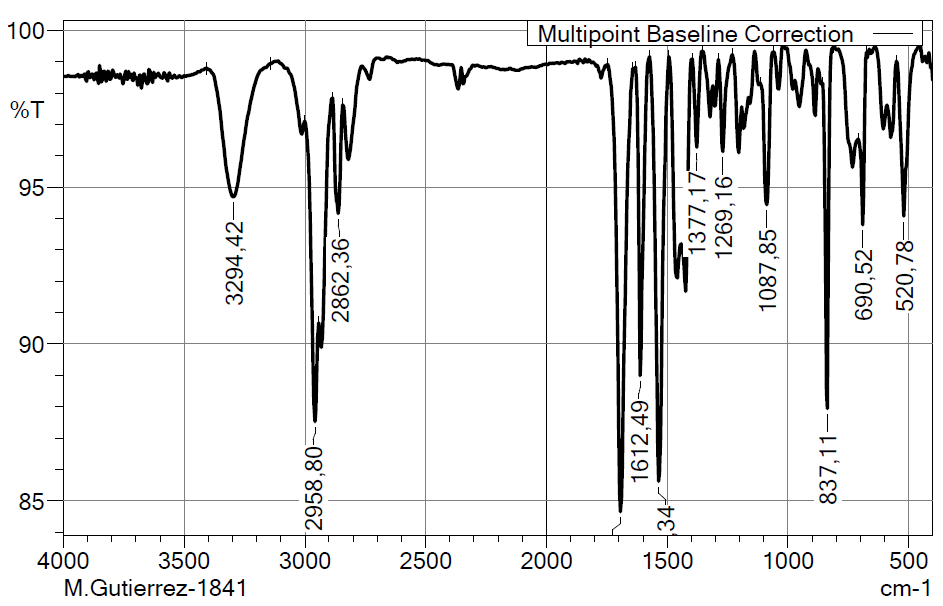
**

Figure S21. IR of 2-(dibutylamino)-*N*-(3,5-dimethylphenyl)acetamide (6c)


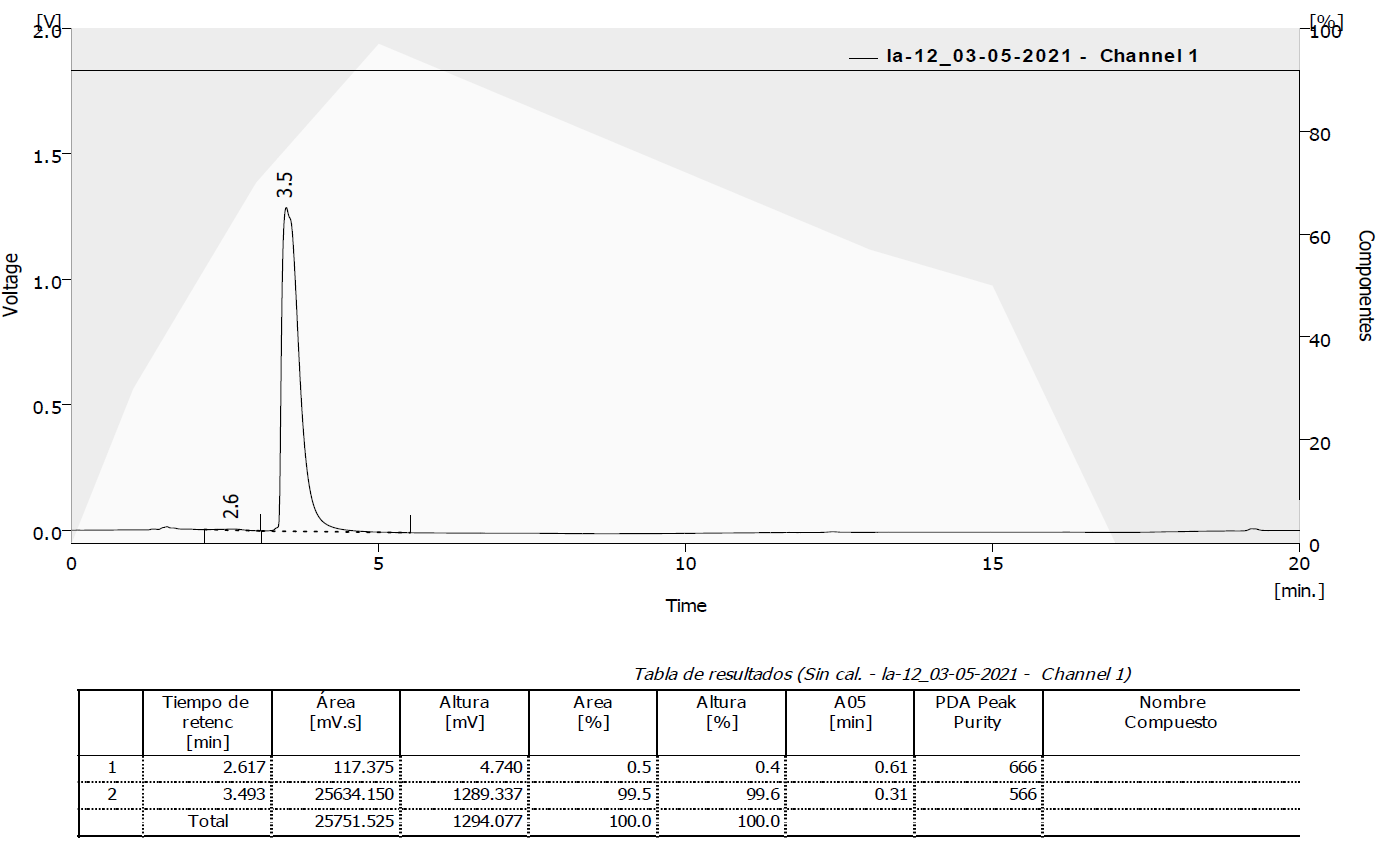


Figure S22. HPLC of of 2-(dibutylamino)-*N*-(3,5-dimethylphenyl)acetamide (6c)


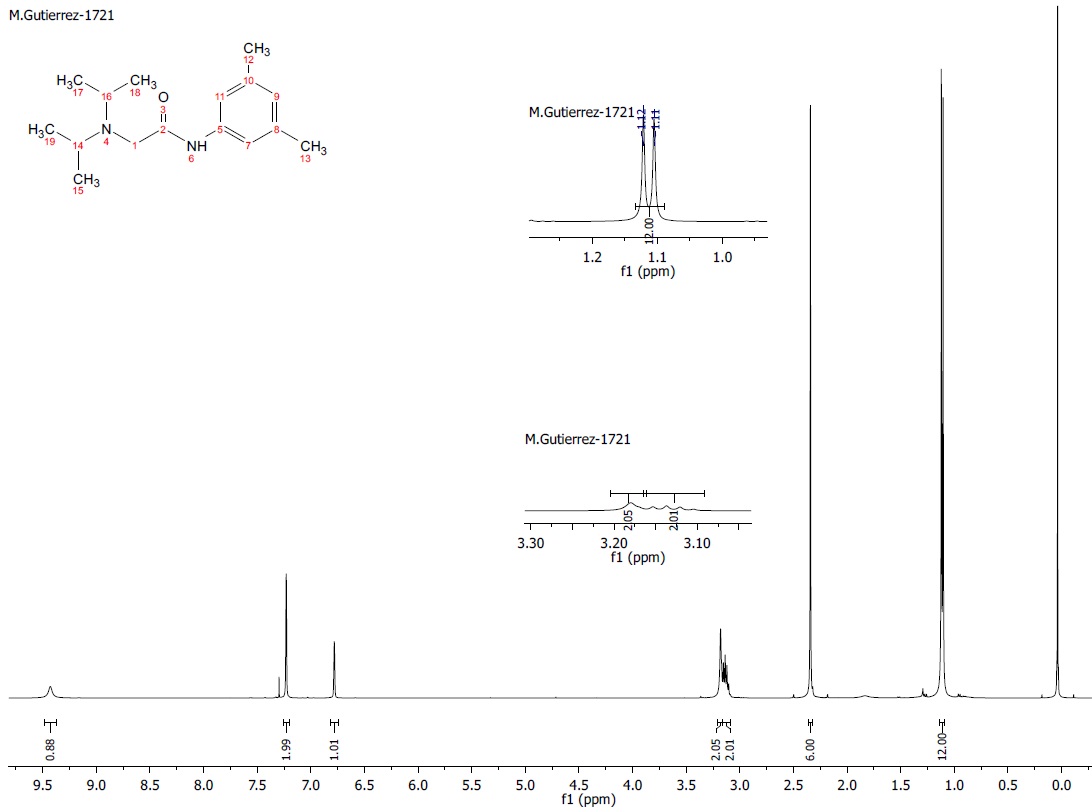


Figure S23. ^1^H-RMN (400 MHz, CDCl_3_) of 2-(diisopropylamino)-*N*-(3,5-dimetylphenyl)acetamide (6d)


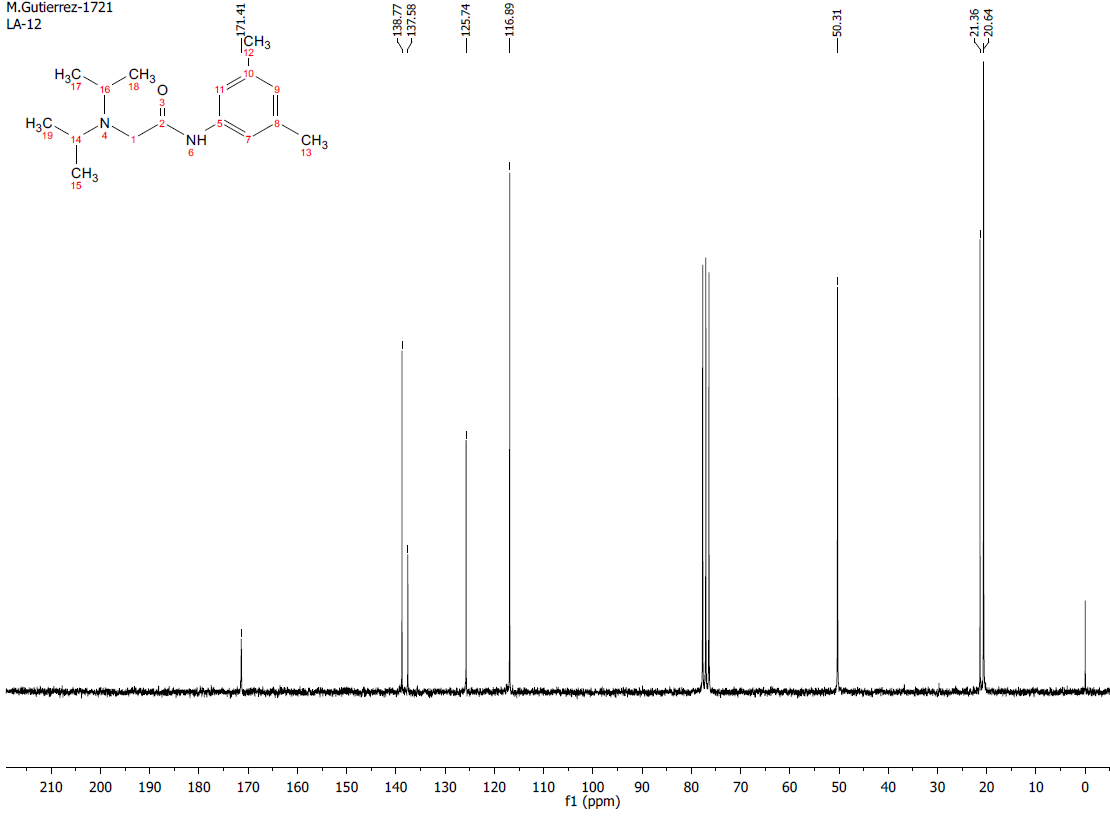


Figure S24. ^13^C-RMN (100 MHz, CDCl_3_) of 2-(diisopropylamino)-*N*-(3,5-dimetylphenyl)acetamide (6d)


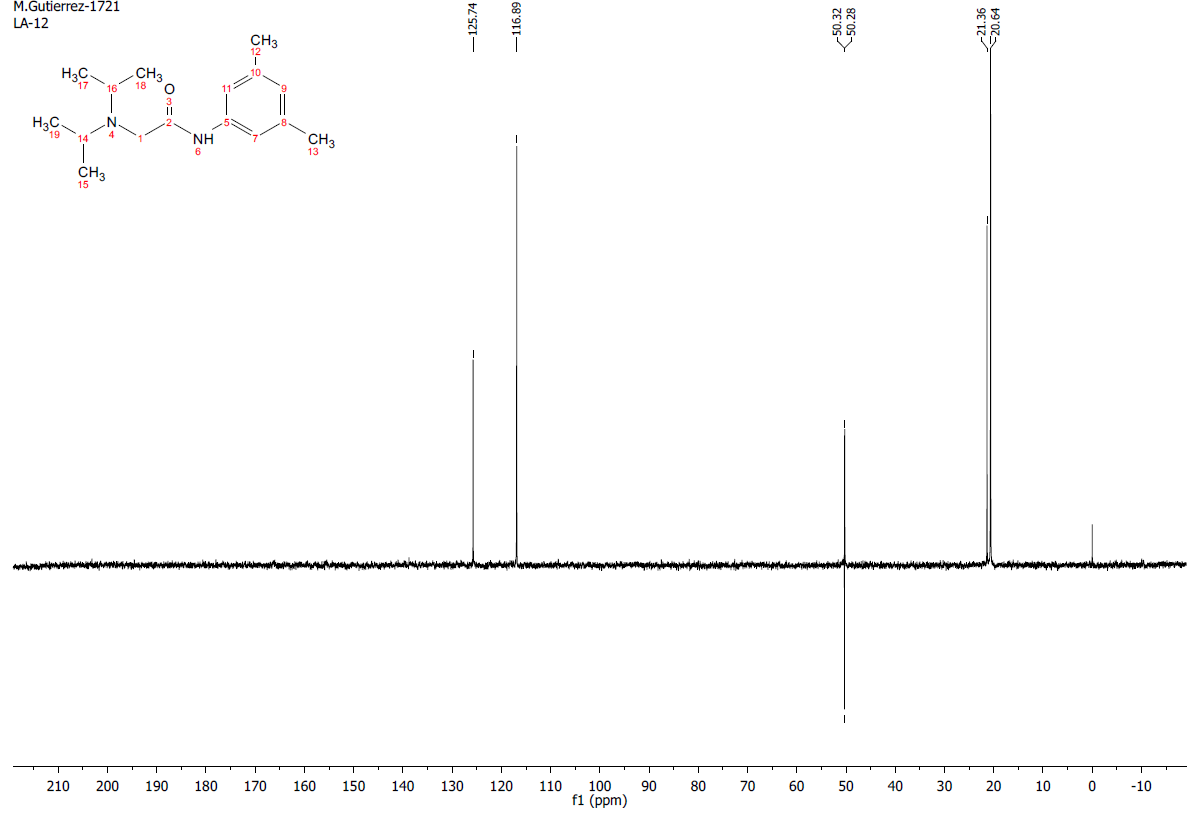


Figure S25. DEPT-135 (CDCl_3_) of 2-(diisopropylamino)-*N*-(3,5-dimetylphenyl)acetamide (6d)


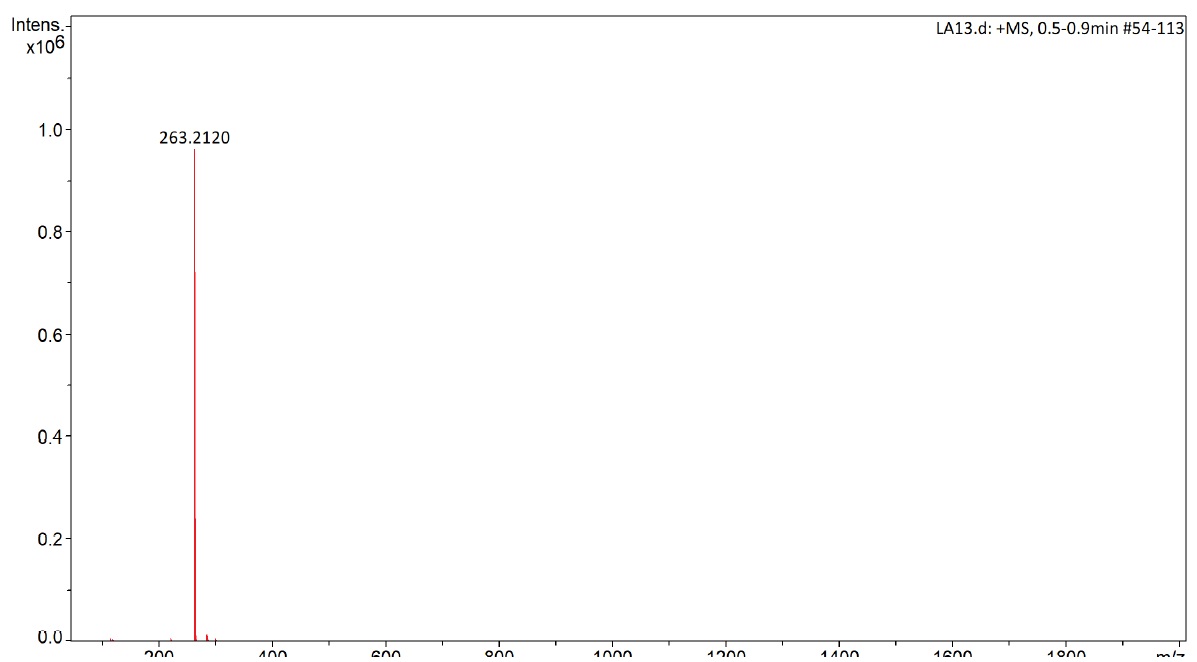


Figure S26. HRMS (ESI, m/z) of 2-(diisopropylamino)-*N*-(3,5-dimetylphenyl)acetamide (6d)

**
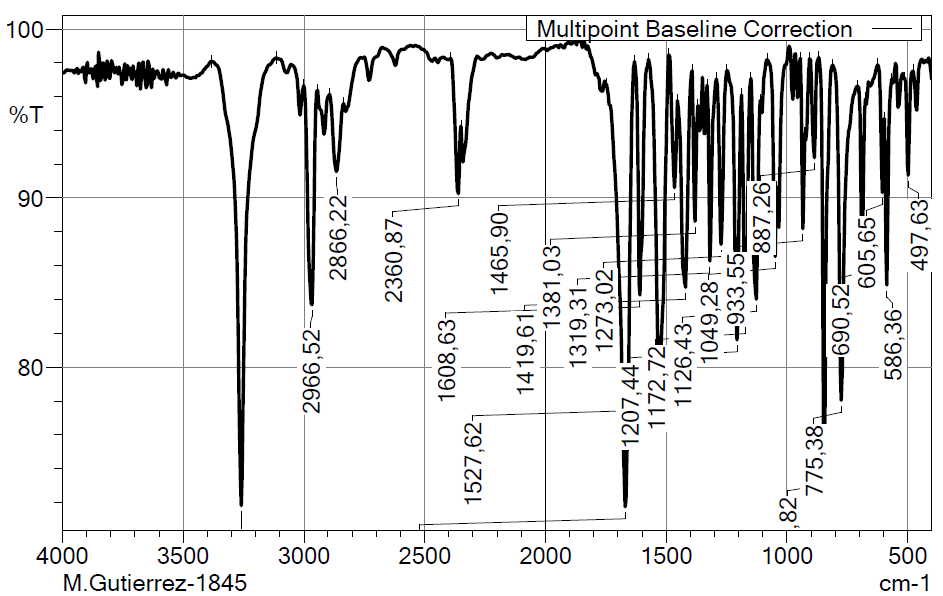
**

Figure S27. IR of of 2-(diisopropylamino)-*N*-(3,5-dimetylphenyl)acetamide (6d)


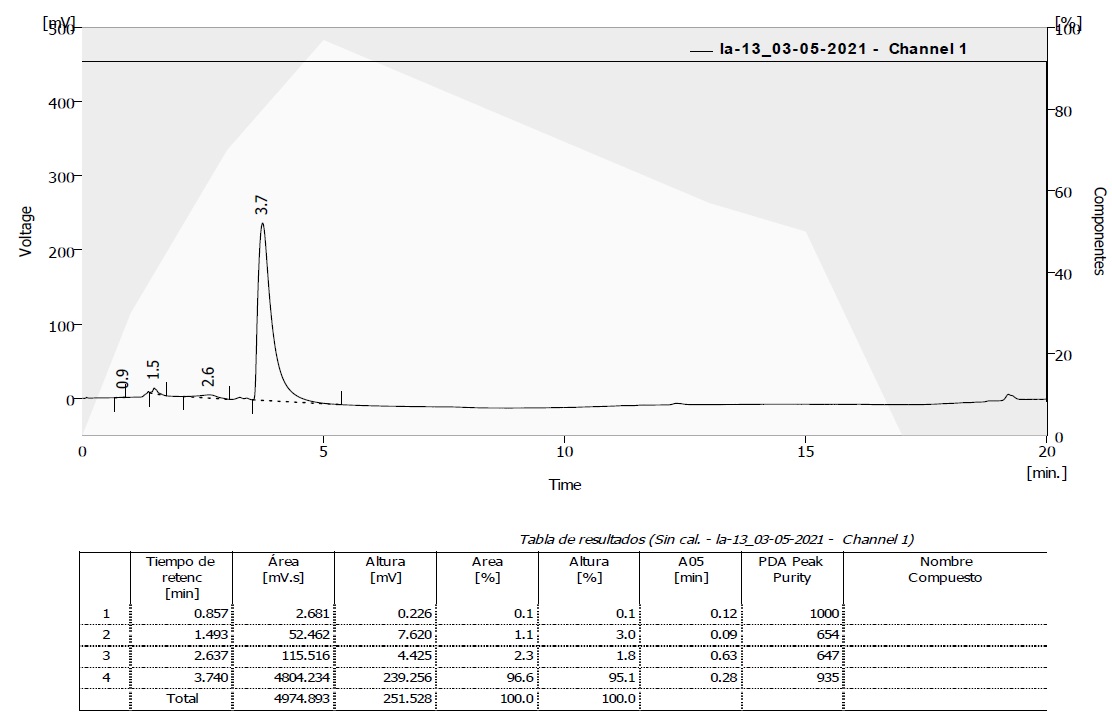


Figure S28. HPLC of 2-(diisopropylamino)-N-(3,5-dimetylphenyl)acetamide (6d)


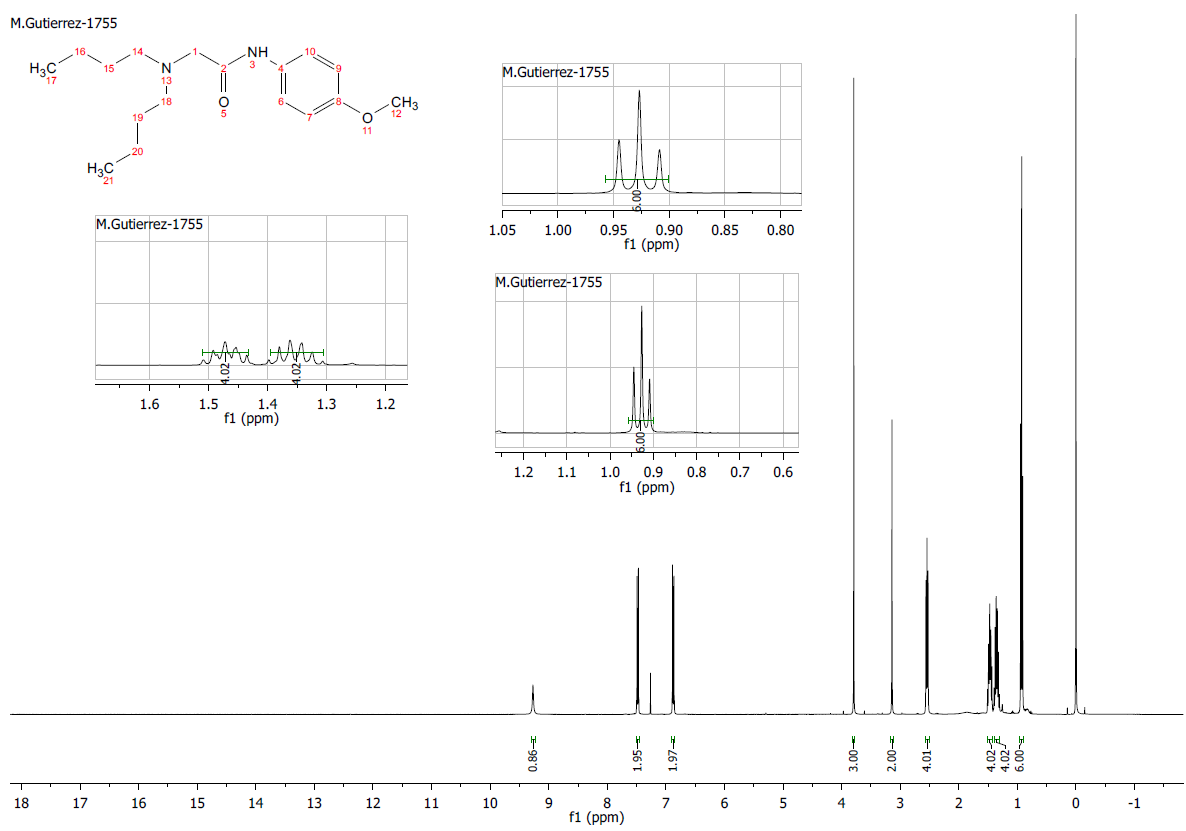


Figure S29. ^1^H-RMN (400 MHz, CDCl_3_) of 2-(dibutylamino)-*N*-(4-methoxiphenyl)acetamide (6e)


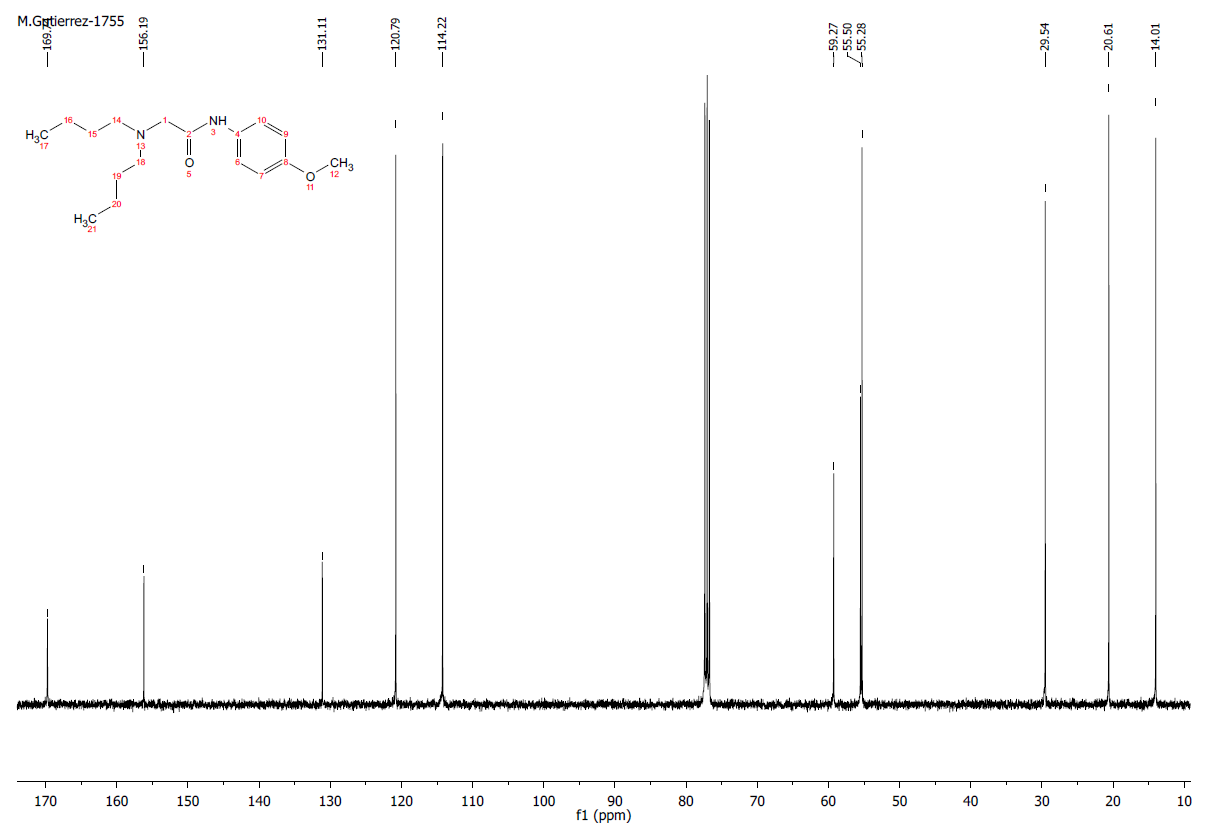


Figure S30. ^13^C-RMN (100 MHz, CDCl_3_) of 2-(dibutylamino)-*N*-(4-methoxiphenyl)acetamide (6e)


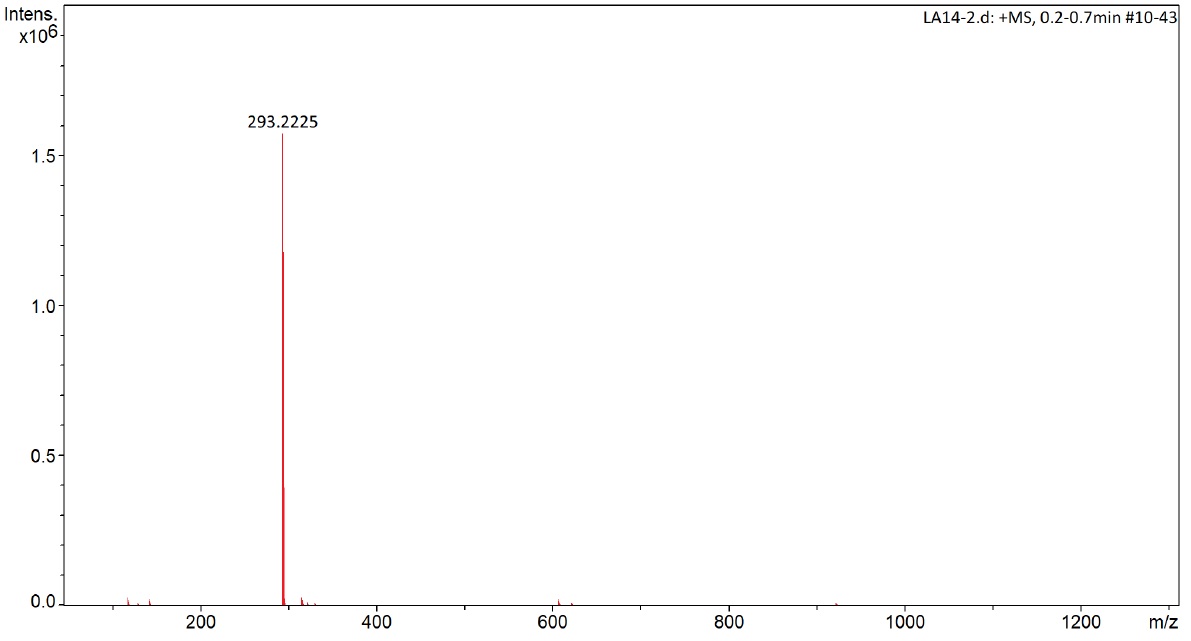


Figure S31. HRMS (ESI, m/z) of 2-(dibutylamino)-*N*-(4-methoxiphenyl)acetamide (6e)

**
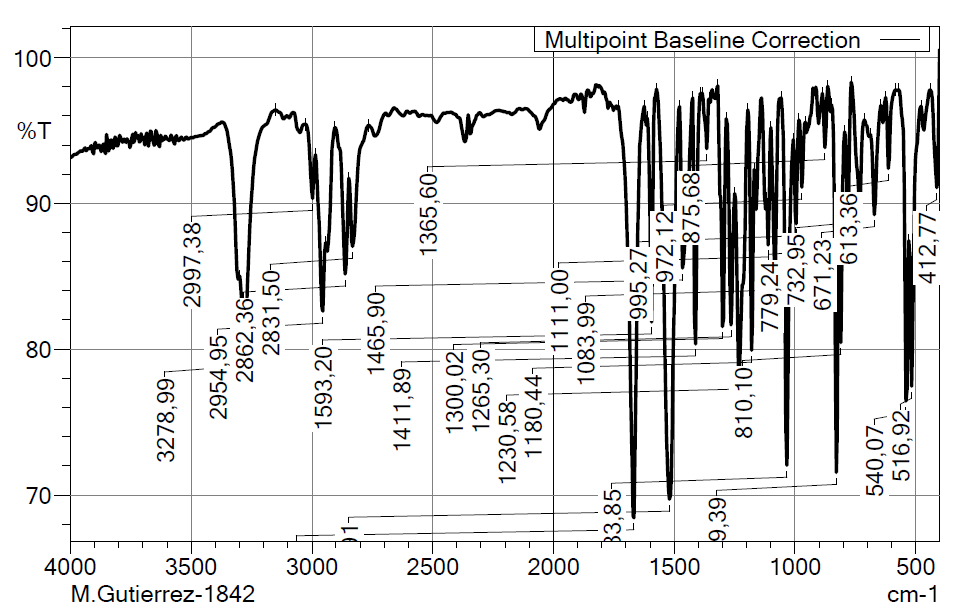
**

Figure S32. IR of 2-(dibutylamino)-*N*-(4-methoxiphenyl)acetamide (6e)


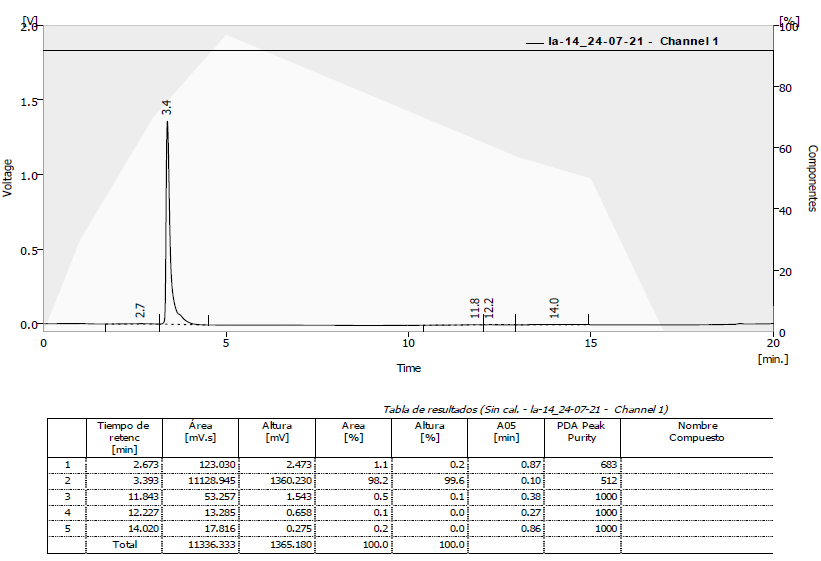


Figure S33. HPLC of 2-(dibutylamino)-*N*-(4-methoxiphenyl)acetamide (6e)


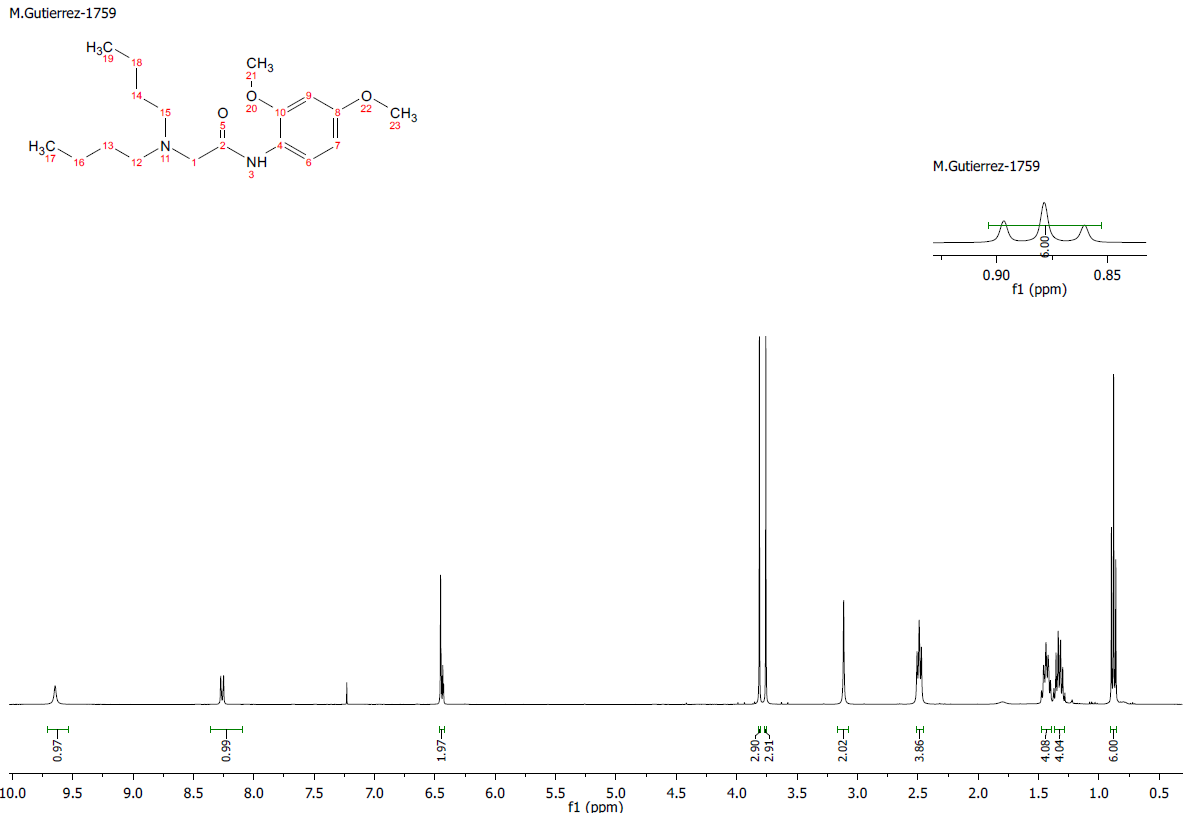


Figure S34. ^1^H-RMN (400 MHz, CDCl_3_)of 2-(dibutylamino)-*N*-(2,4-dimethoxiphenyl)acetamide (6f)


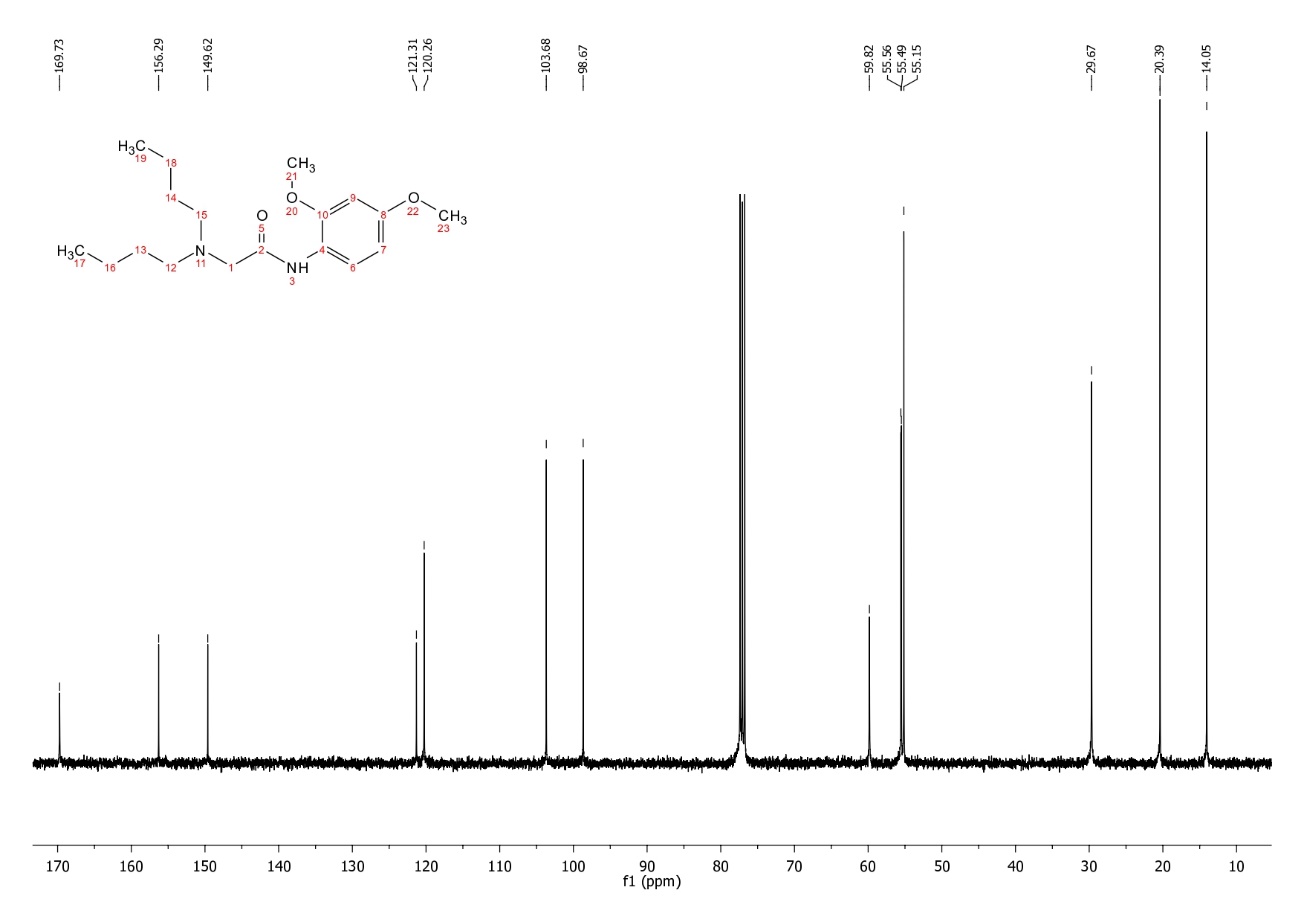


Figure S35. ^13^C-RMN (100 MHz, CDCl_3_) of 2-(dibutylamino)-*N*-(2,4-dimethoxiphenyl)acetamide (6f)


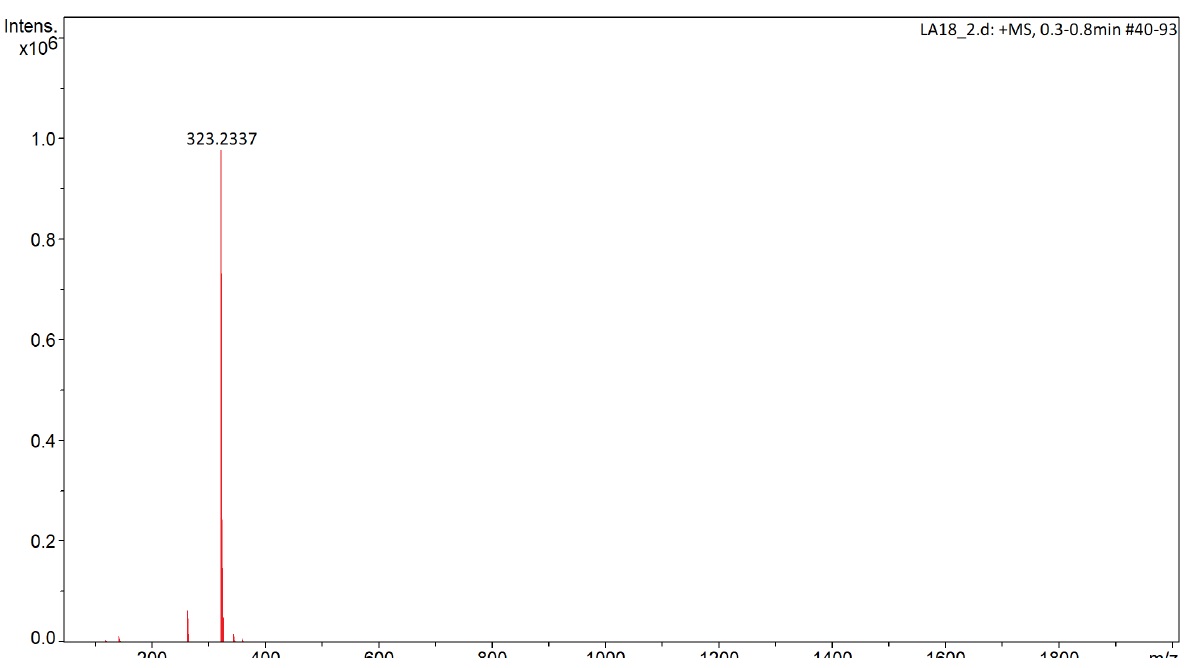


Figure S36. HRMS (ESI, m/z) of 2-(dibutylamino)-*N*-(2,4-dimethoxiphenyl)acetamide (6f)

**
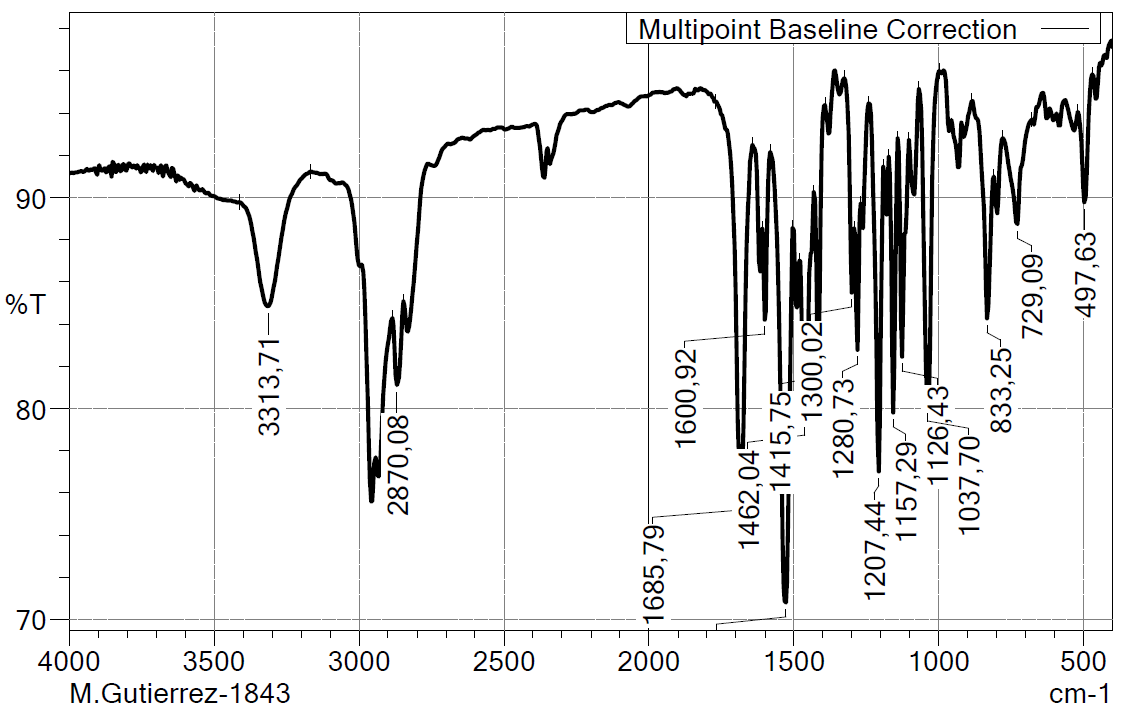
**

Figure S37. IR of 2-(dibutylamino)-*N*-(2,4-dimethoxiphenyl)acetamide (6f)


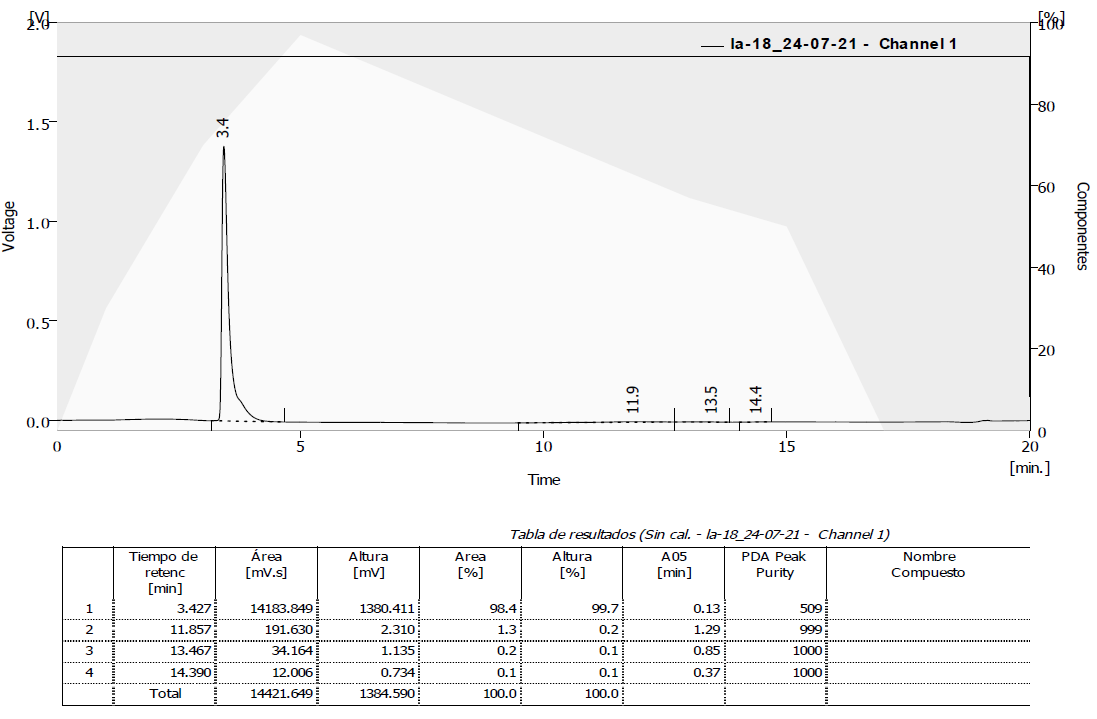


Figure S38. HPLC of 2-(dibutylamino)-*N*-(2,4-dimethoxiphenyl)acetamide (6f)


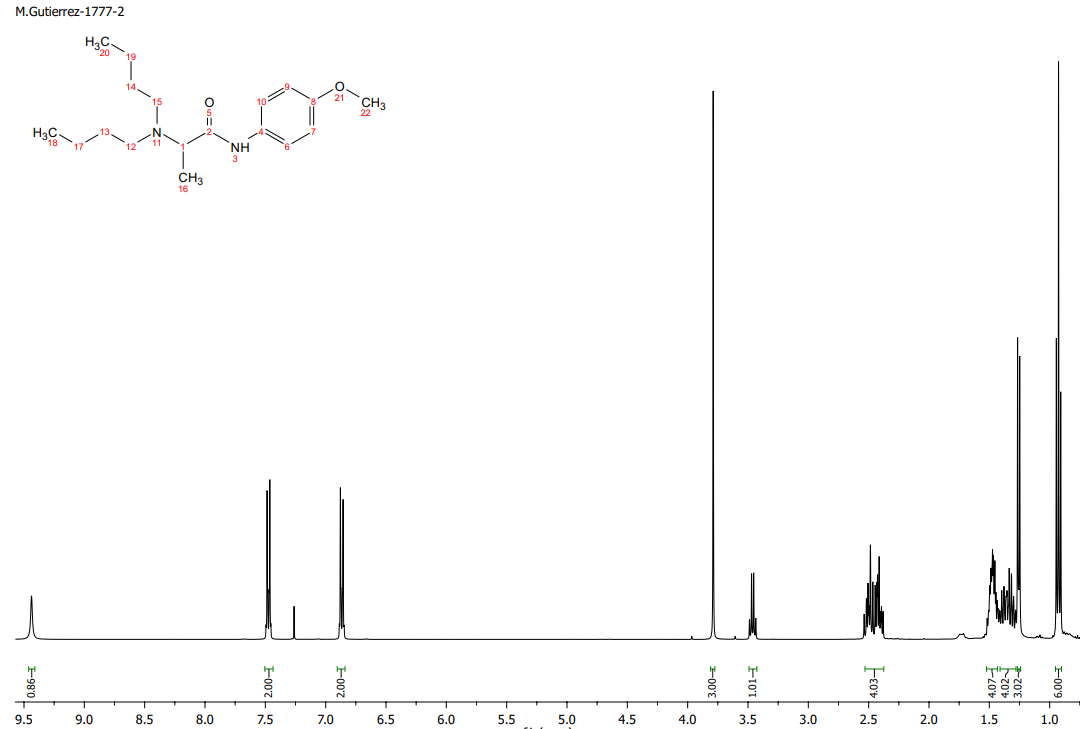


Figure S39. ^1^H-RMN (400 MHz, CDCl_3_)of 2-(dibutylamino)-*N*-(4-methoxiphenyl)propanamide (7a)


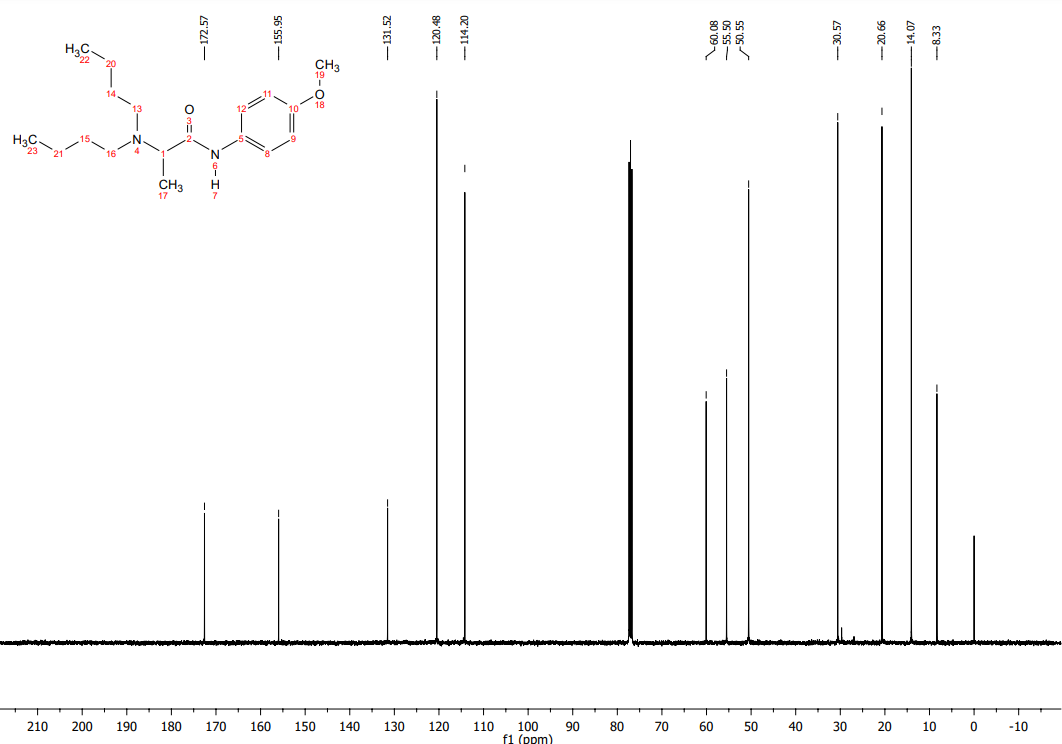


Figure S40. ^13^C-RMN (100 MHz, CDCl_3_) 2-(dibutylamino)-*N*-(4-methoxiphenyl)propanamide (7a)


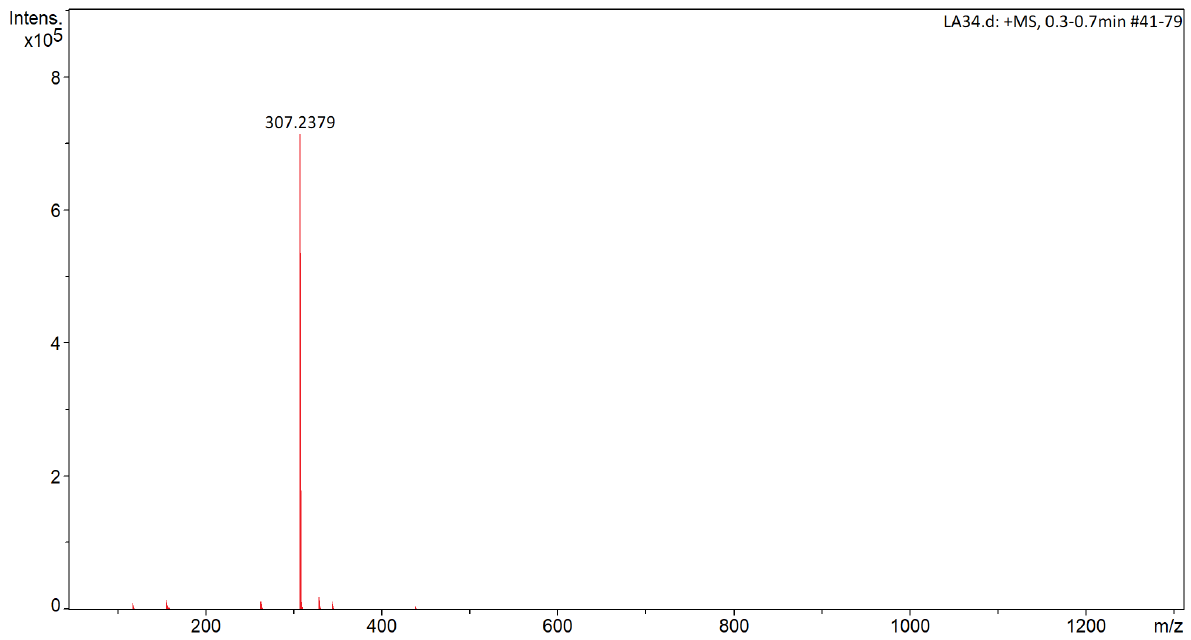


Figure S41. HRMS (ESI, m/z) of 2-(dibutylamino)-*N*-(4-methoxiphenyl)propanamide (7a)


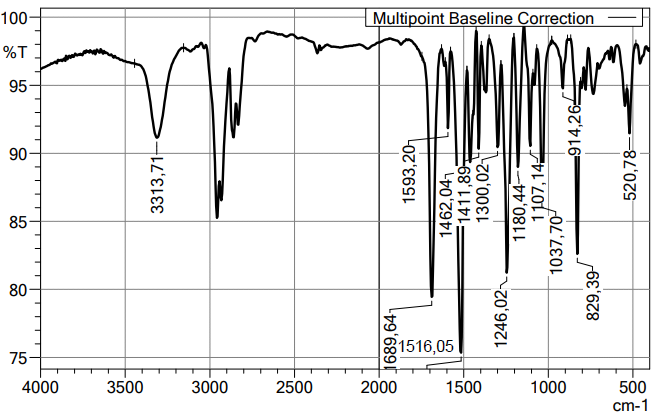


Figure S42. IR of 2-(dibutylamino)-*N*-(4-methoxiphenyl)propanamide (7a)


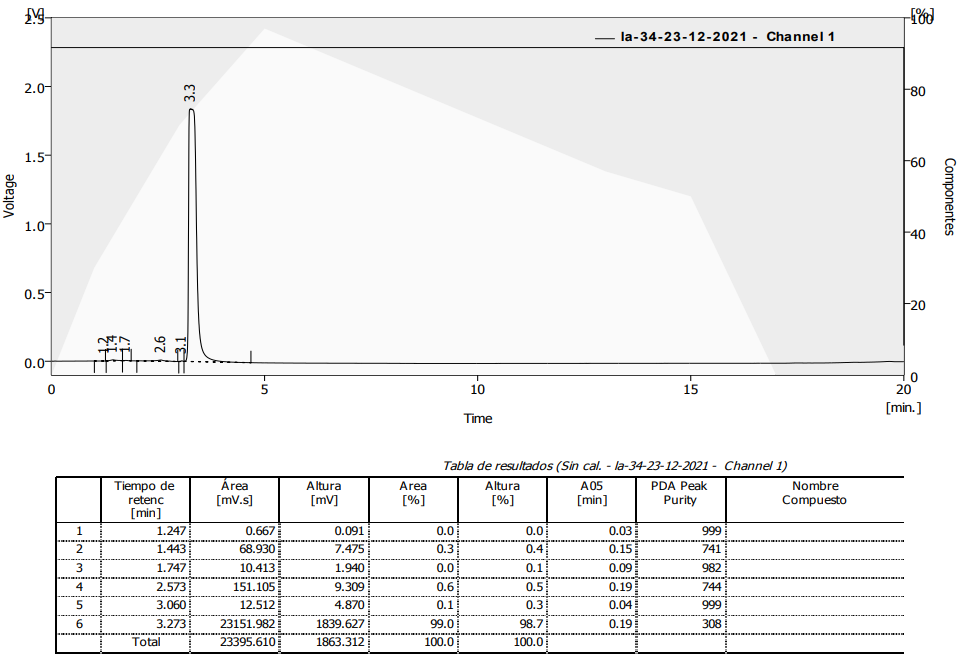


Figure S43. HPLC of 2-(dibutylamino)-*N*-(4-methoxiphenyl)propanamide (7a)


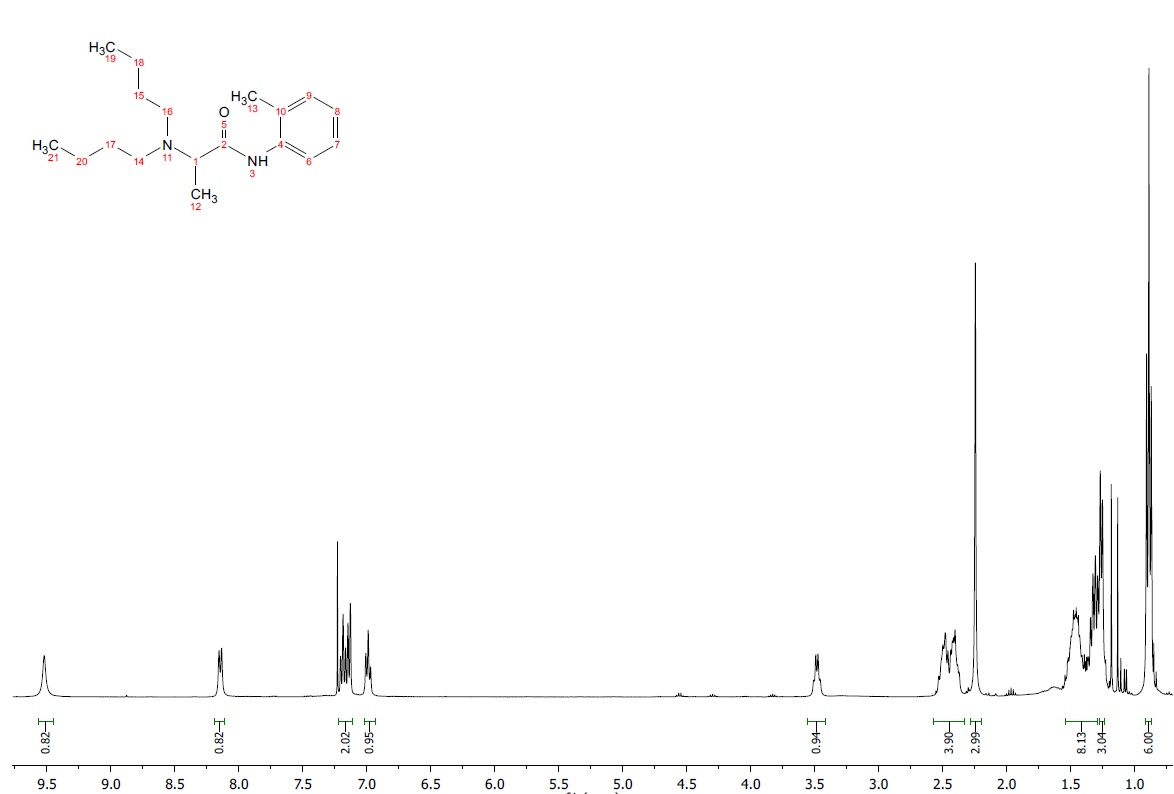


Figure S44. ^1^H-RMN (400 MHz, CDCl_3_) of 2-(dibutylamino)-*N*-(o-tolyl)propanamide (7b)


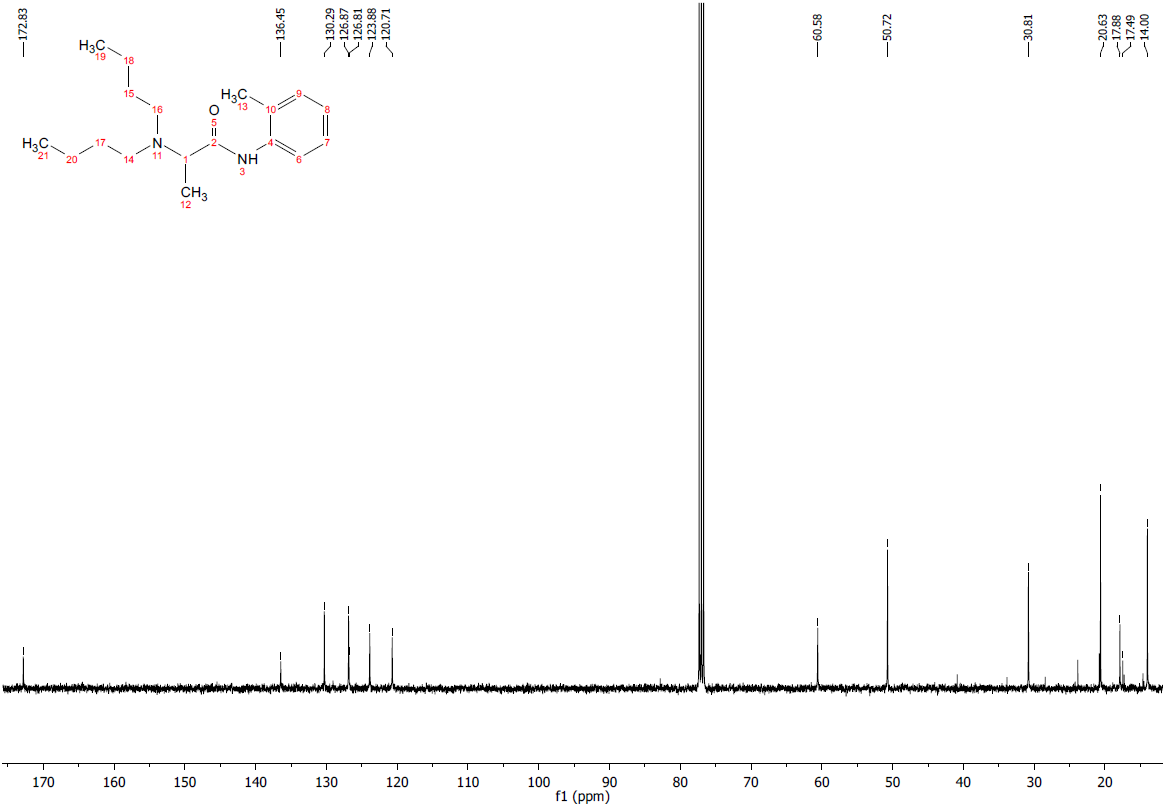


Figure S45. ^13^C-RMN (100 MHz, CDCl_3_) of 2-(dibutylamino)-*N*-(o-tolyl)propanamide (7b)


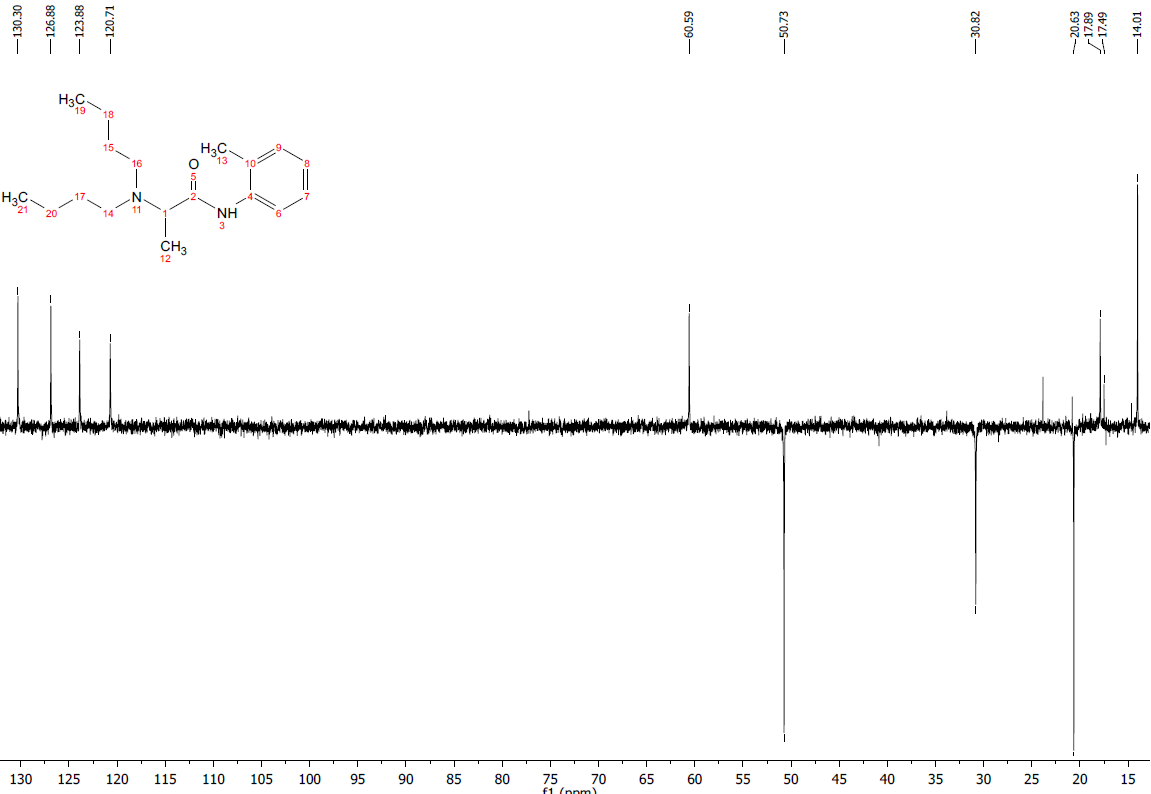


Figure S46. DEPT-135 (CDCl_3_) of 2-(dibutylamino)-*N*-(o-tolyl)propanamide (7b)


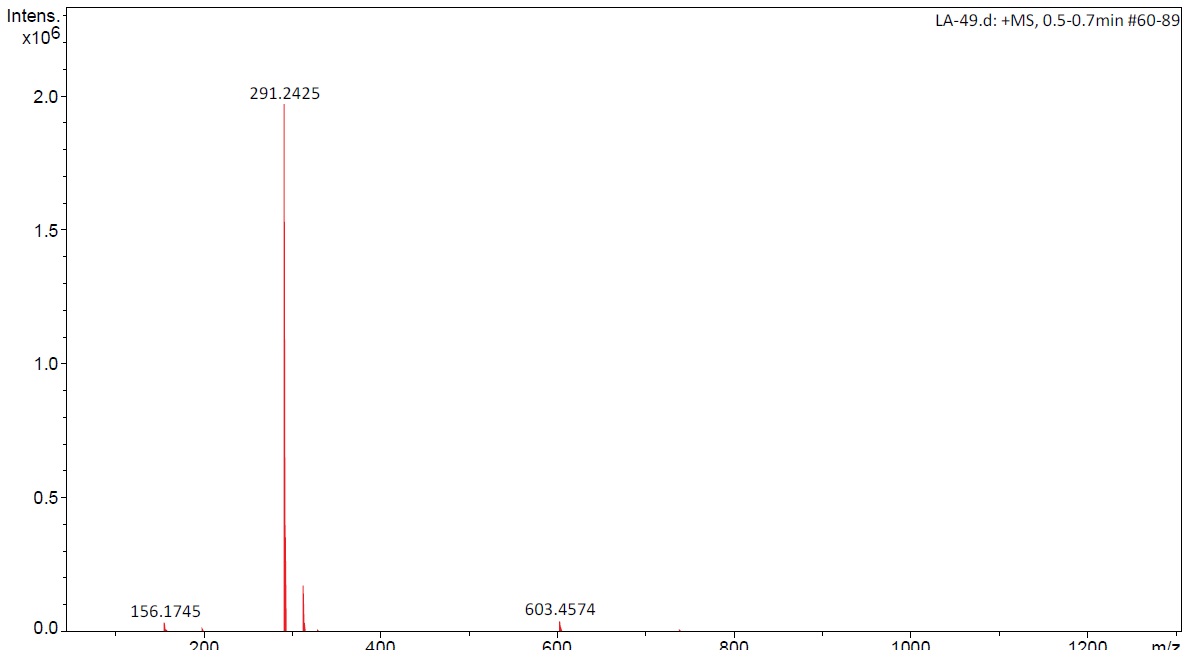


Figure S47. HRMS (ESI, m/z) of 2-(dibutylamino)-*N*-(o-tolyl)propanamide (7b)


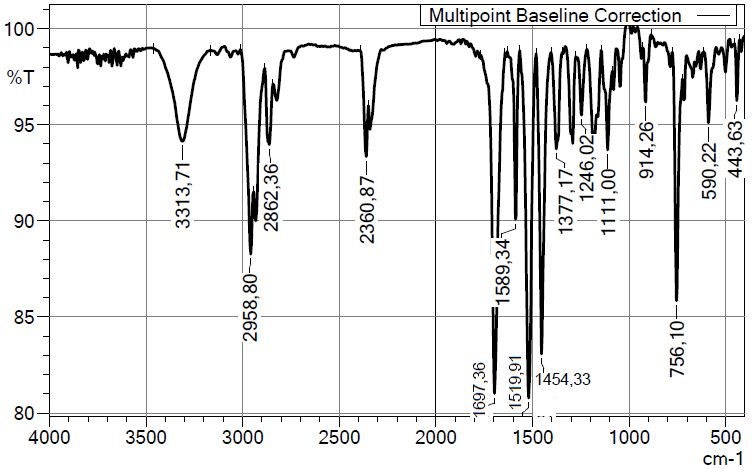


Figure S48. IR of 2-(dibutylamino)-*N*-(o-tolyl)propanamide (7b)


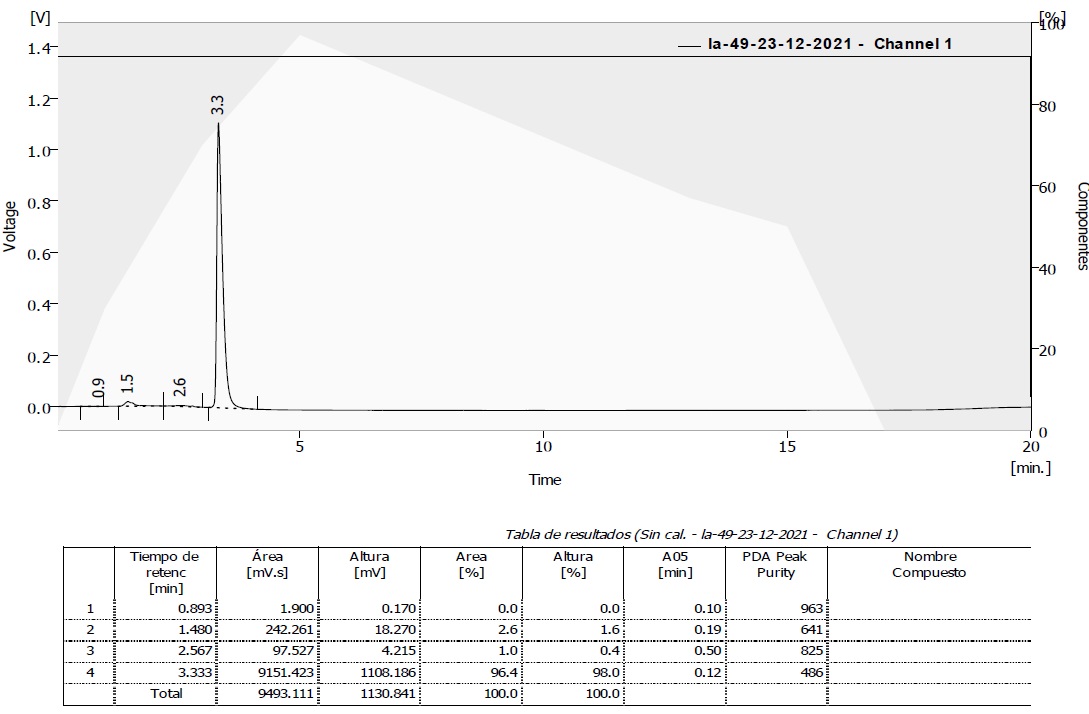


Figure S49. HPLC of 2-(dibutylamino)-*N*-(o-tolyl)propanamide (7b)


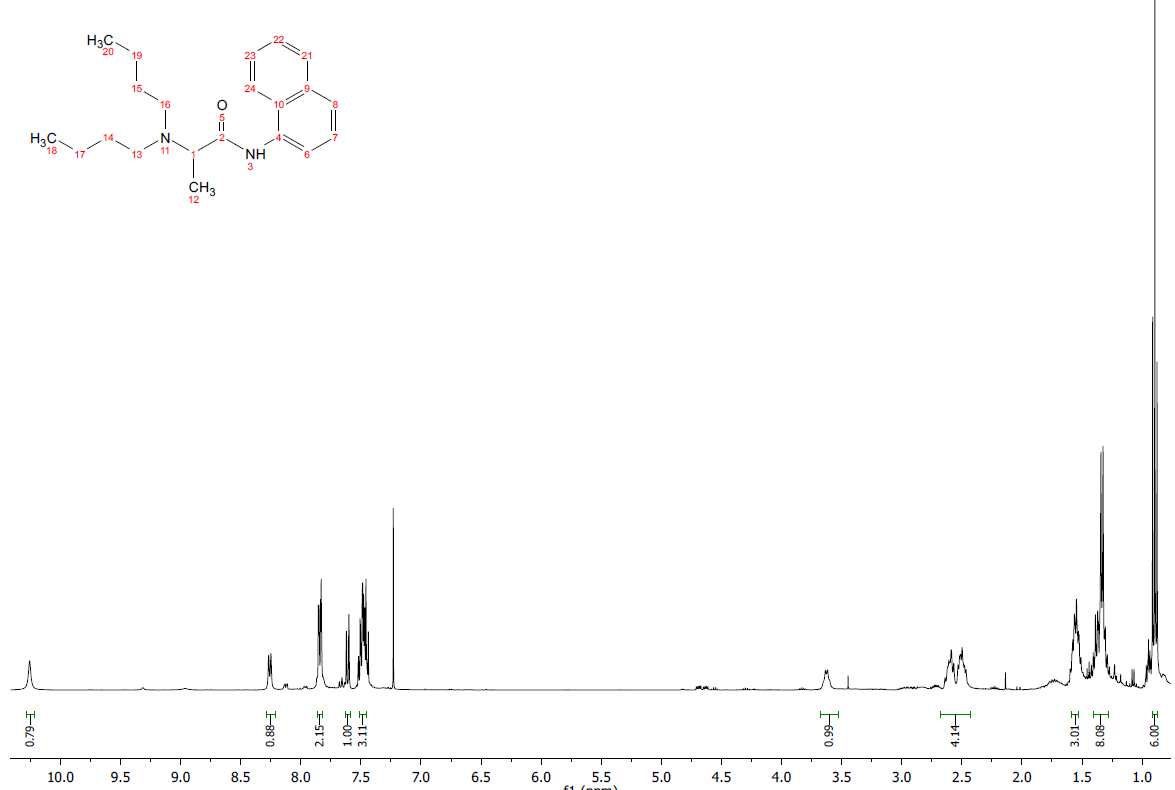


Figure S50. ^1^H-RMN (400 MHz, CDCl_3_)of 2-(dibutylamino)-*N*-(naphthalen-1-yl)propanamide (7c)


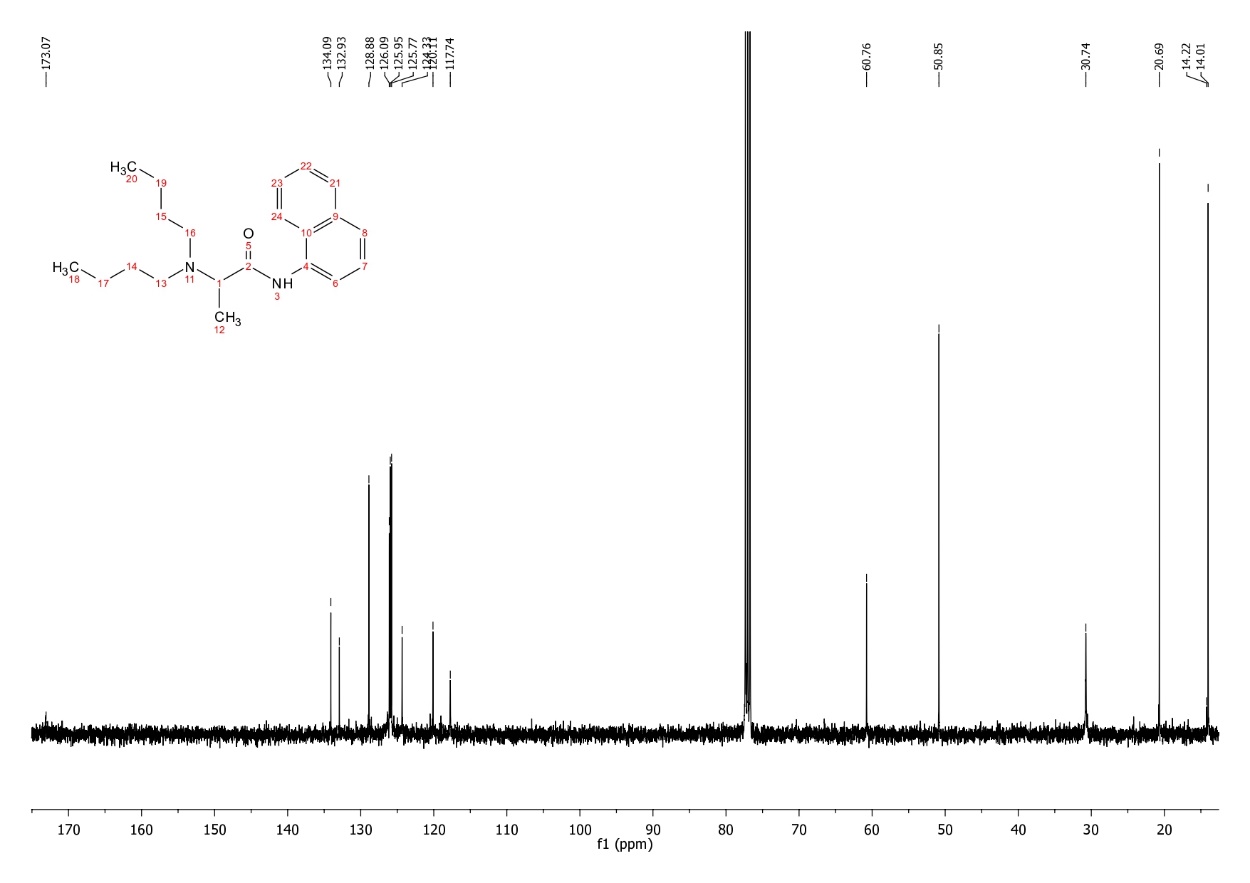


Figure S51. ^13^C-RMN (100 MHz, CDCl_3_) of 2-(dibutylamino)-*N*-(naphthalen-1-yl)propanamide (7c)


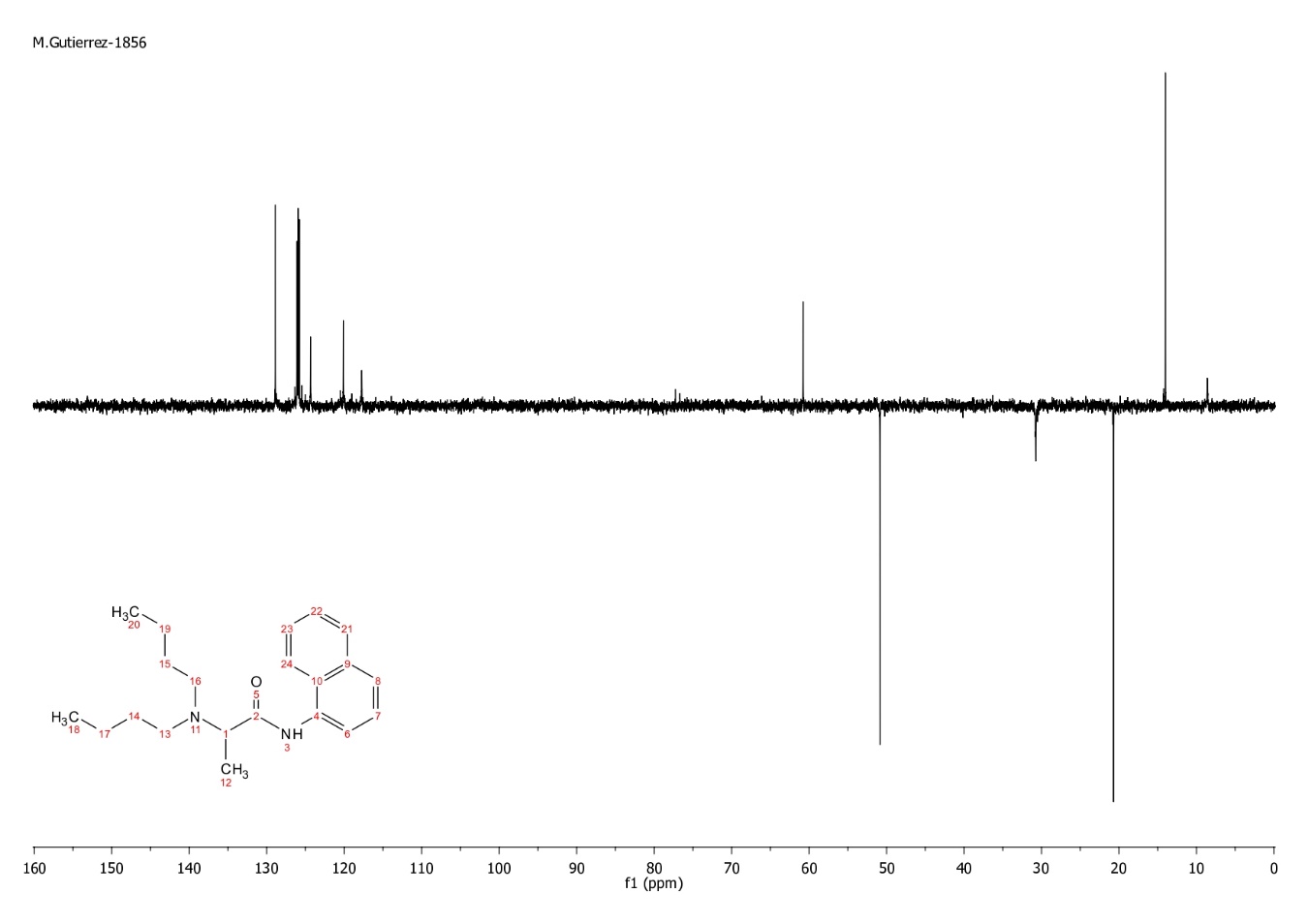


Figure S52. DEPT-135 (CDCl_3_) of 2-(dibutylamino)-*N*-(naphthalen-1-yl)propanamide (7c)


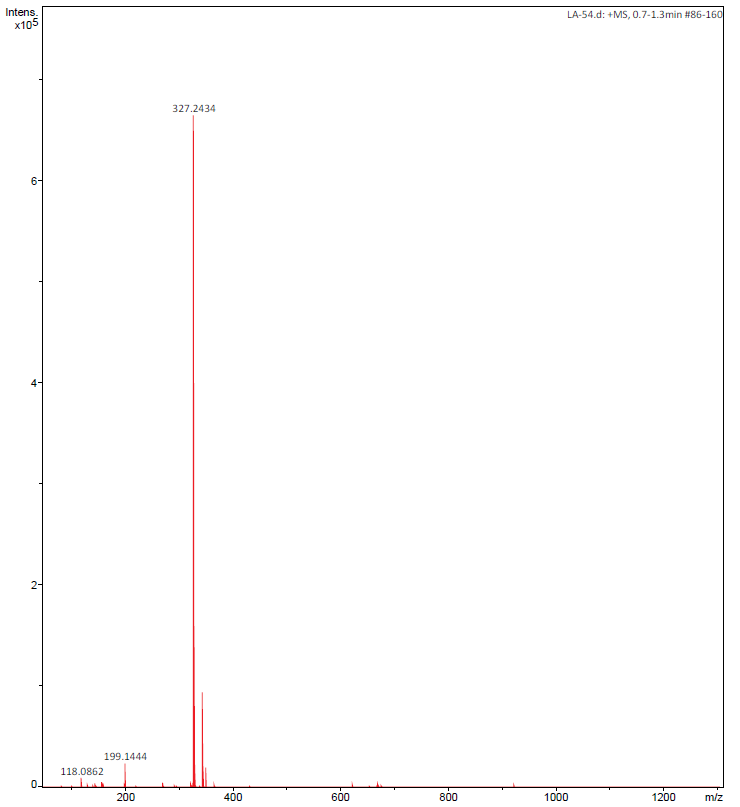


Figure S53. HRMS (ESI, m/z) of 2-(dibutylamino)-*N*-(naphthalen-1-yl)propanamide (7c)


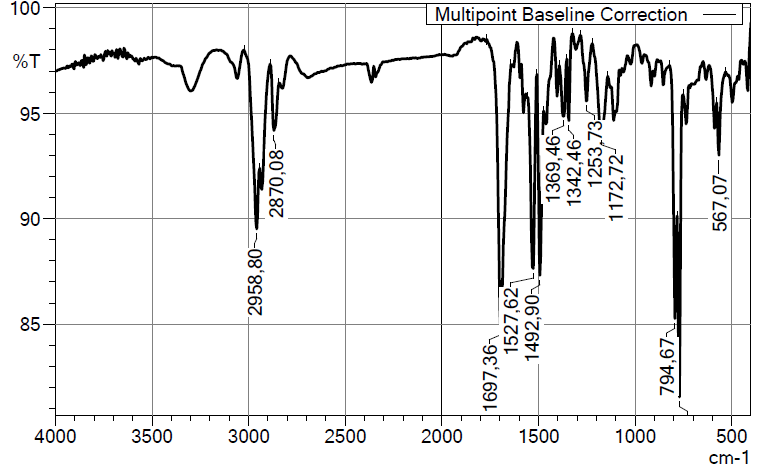


Figure S54. IR of 2-(dibutylamino)-*N*-(naphthalen-1-yl)propanamide (7c)


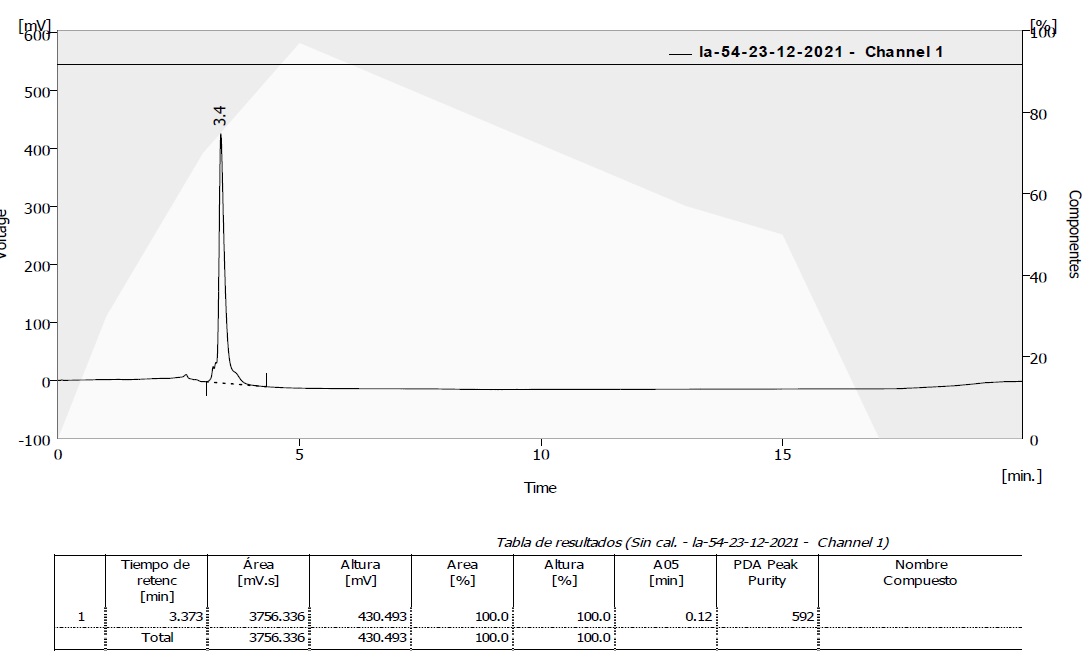


Figure S55. HPLC of 2-(dibutylamino)-*N*-(naphthalen-1-yl)propanamide (7c)


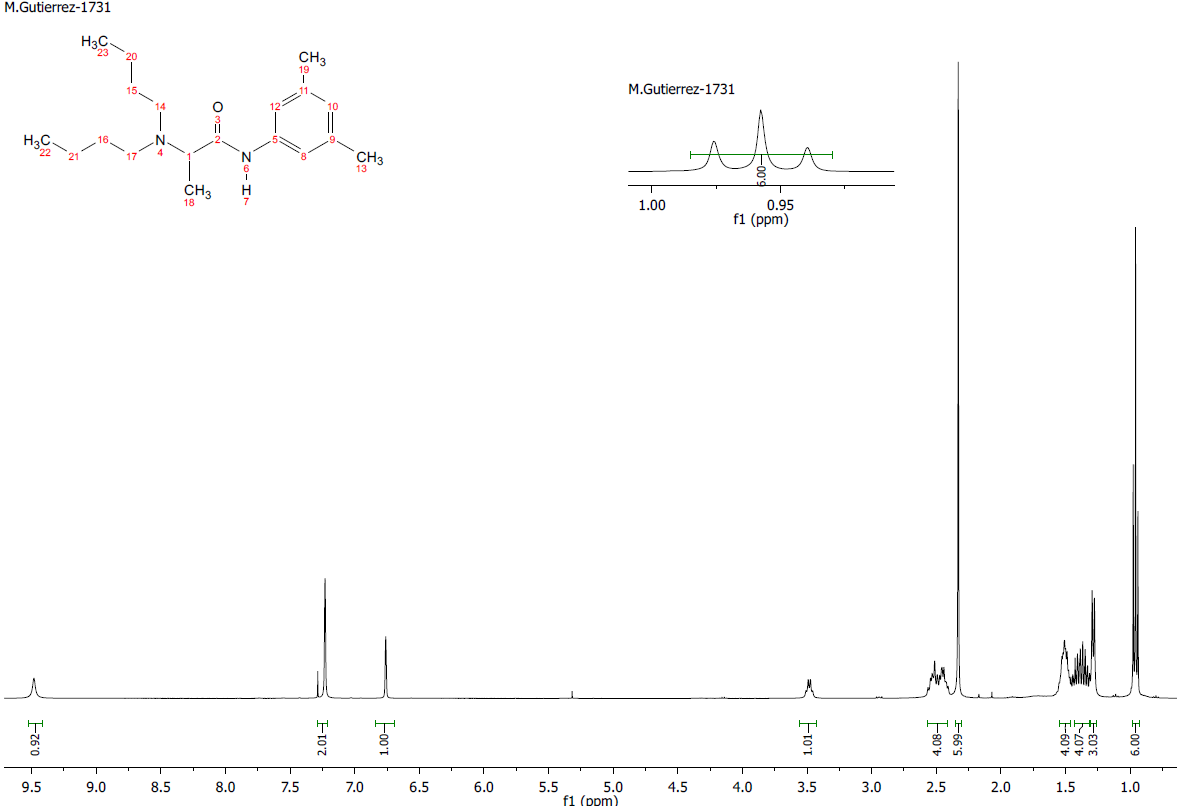


Figure S56. ^1^H-RMN (400 MHz, CDCl_3_)of 2-(dibutylamino)-*N*-(3,5-dimetilphenyl)propanamide (7d)


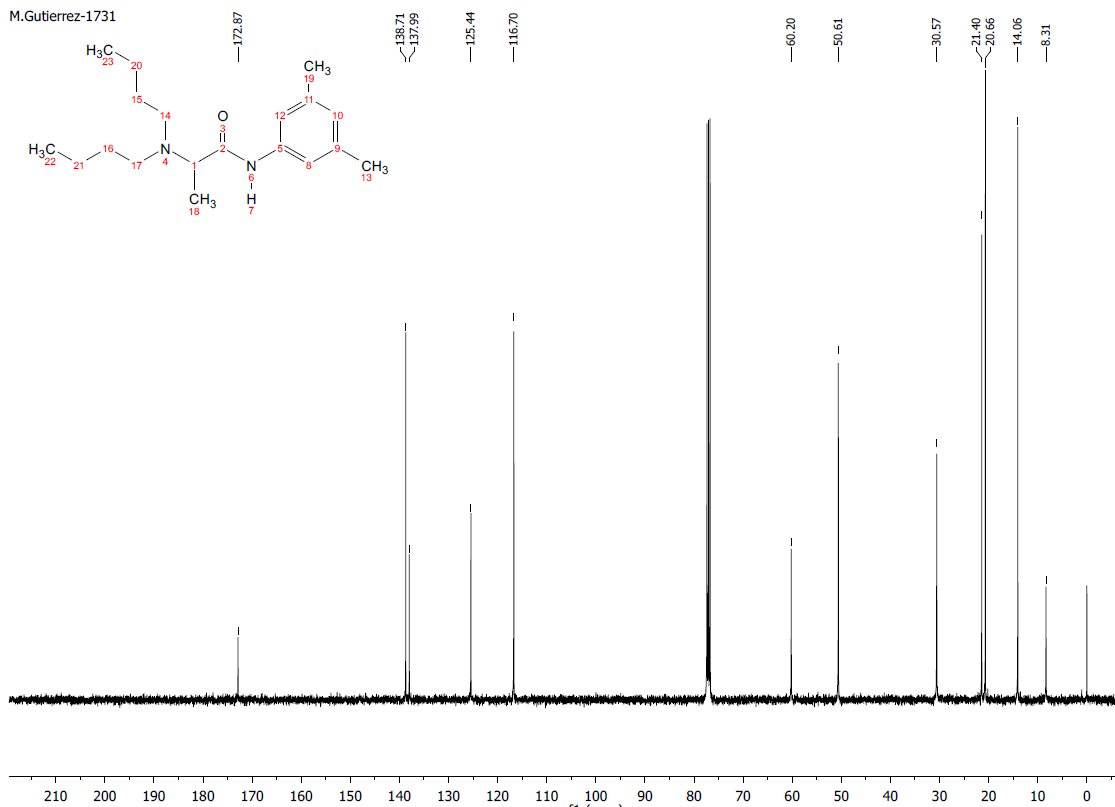


Figure S57. DEPT-135 (CDCl_3_) of 2-(dibutylamino)-*N*-(3,5-dimetilphenyl)propanamide (7d)


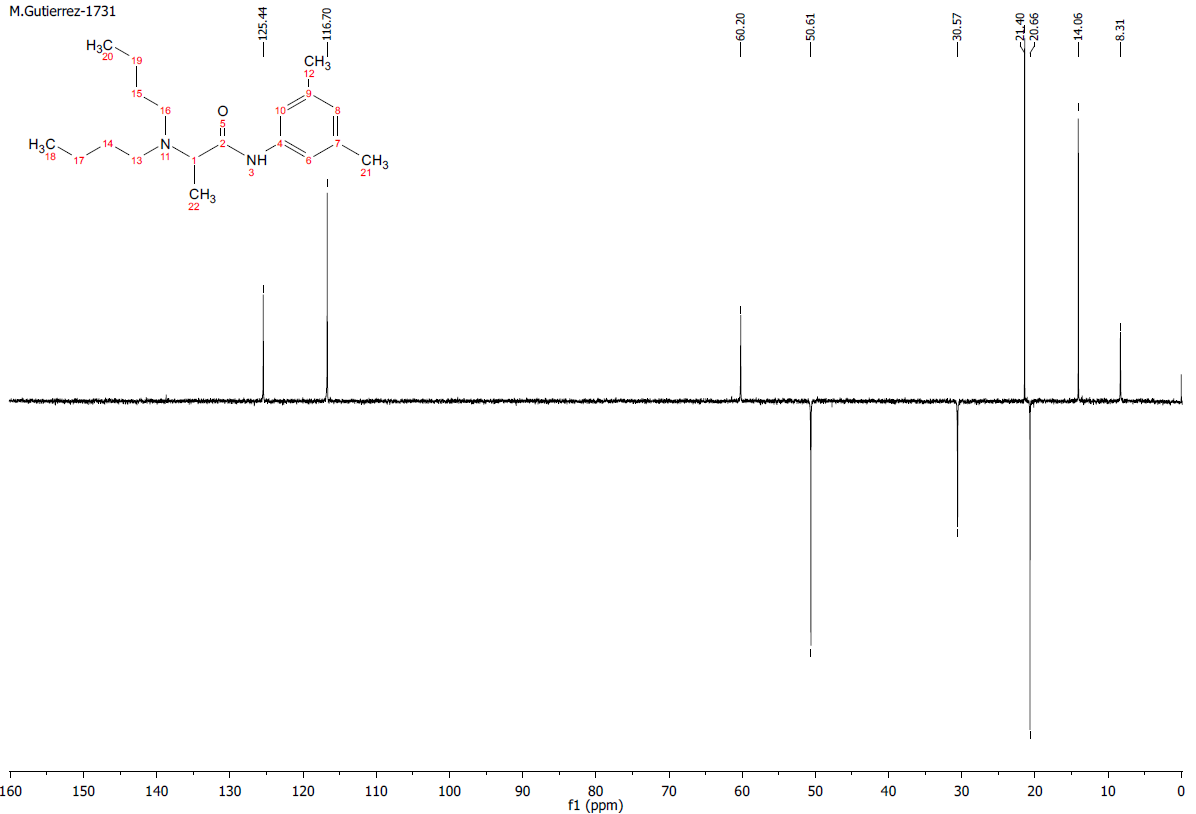


Figure S58. DEPT-135 (CDCl_3_) of 2-(dibutylamino)-*N*-(3,5-dimetilphenyl)propanamide (7d)


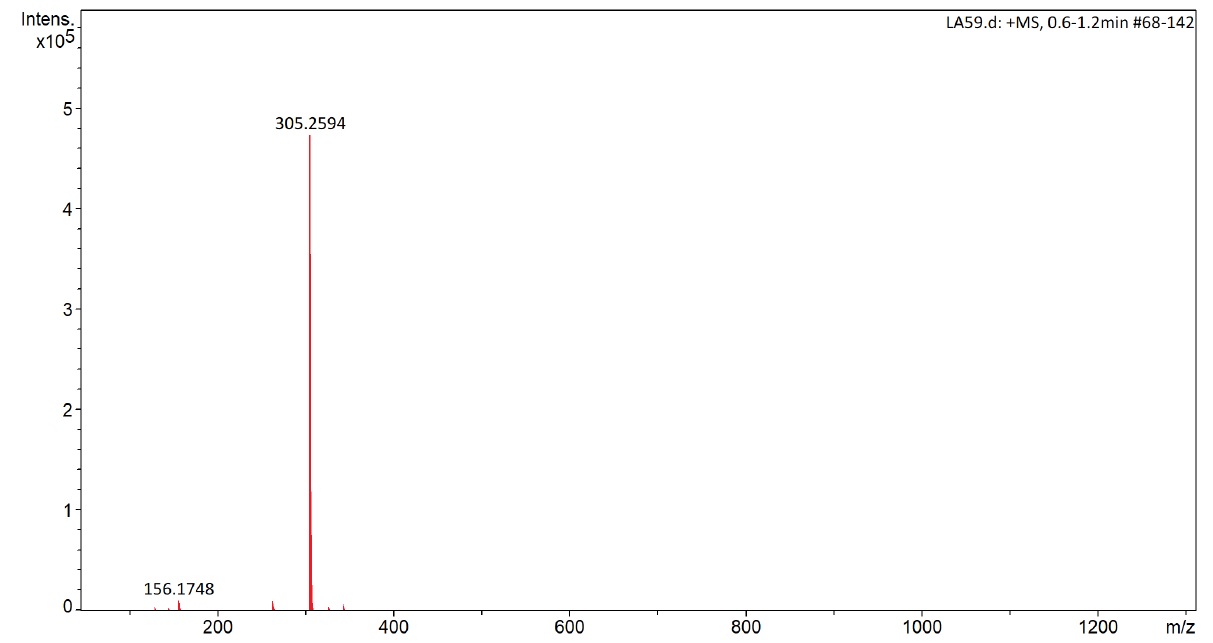


Figure S59. HRMS (ESI, m/z) of 2-(dibutylamino)-*N*-(3,5-dimetilphenyl)propanamide (7d)


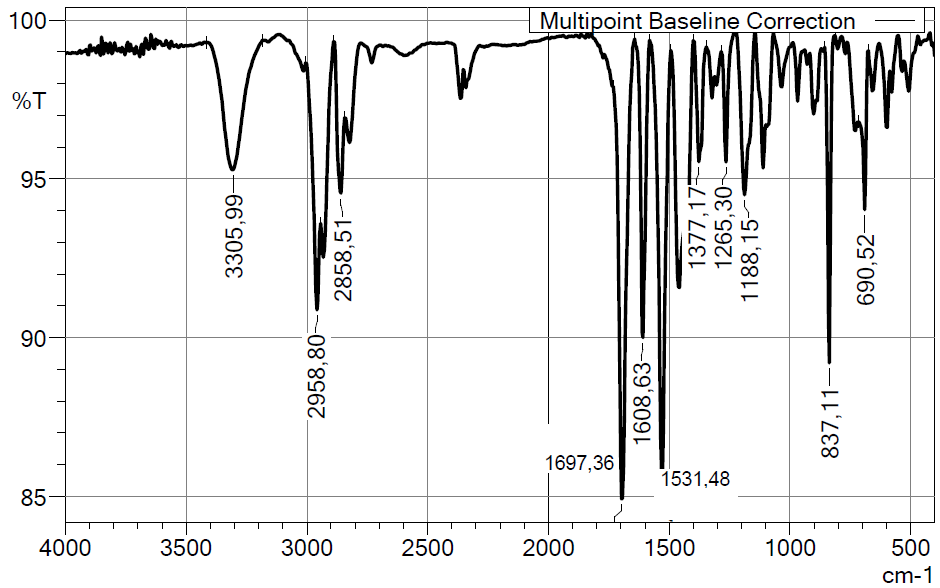


Figure S60. IR of 2-(dibutylamino)-*N*-(3,5-dimetilphenyl)propanamide (7d)


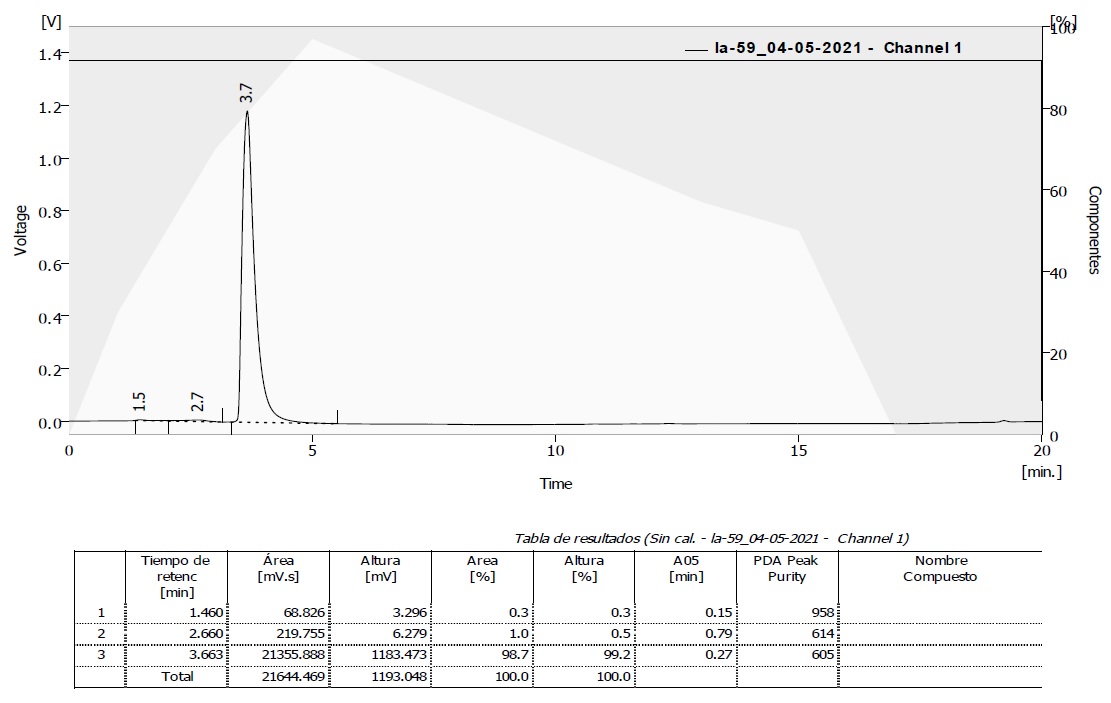


Figure S61. HPLC of 2-(dibutylamino)-*N*-(3,5-dimetilphenyl)propanamide (7d)

**Table S1. Ranking of poses of the 65 compounds in Kv1.5 – central cavity. INDEX:** Corresponds to the pose number of each compound within the 10 poses generated in the IFD protocol. **DGBIND:** corresponds to the binding free energy value calculated through MM-GBSA. **INT:** is the number of LA binding site residues within 5 Å of the ligand. **DGBIND_NORM:** corresponds to the normalization value of the binding free energy (most favorable energy values tend to be 1). **INT_NORM**: corresponds to the normalization value with respect to the number of interactions with the binding site residues (the greater the number of interactions tend to 1). **NORMT:** Sum of the normalization values divided by two. **RANK:** Position of the compound in the ranking according to the normalization value and number of interactions.

| **NAME** | **INDEX** | **DGBIND** | **INT** | **DGBIND_NORM** | **INT_NORM** | **NORMT** | **RANK** |
| --- | --- | --- | --- | --- | --- | --- | --- |
| 7a | 3 | -67.60 | 12 | 0.69 | 1.00 | 0.85 | 1 |
| bupivacaineS | 0 | -62.69 | 11 | 0.64 | 0.86 | 0.75 | 2 |
| bupivacaineR | 5 | -54.49 | 11 | 0.56 | 0.86 | 0.71 | 3 |
| 6f | 8 | -42.04 | 11 | 0.44 | 0.86 | 0.65 | 4 |
| ropivacaineS | 7 | -41.67 | 11 | 0.43 | 0.86 | 0.64 | 5 |
| 7h | 3 | -40.18 | 11 | 0.42 | 0.86 | 0.64 | 6 |
| 7x | 2 | -37.10 | 11 | 0.39 | 0.86 | 0.62 | 7 |
| 6x | 3 | -35.59 | 11 | 0.37 | 0.86 | 0.61 | 8 |
| 6z | 8 | -32.45 | 11 | 0.34 | 0.86 | 0.60 | 9 |
| 7d | 3 | -76.12 | 10 | 0.78 | 0.71 | 0.75 | 10 |
| 6d | 0 | -70.49 | 10 | 0.72 | 0.71 | 0.72 | 11 |
| 7c | 3 | -63.55 | 10 | 0.65 | 0.71 | 0.68 | 12 |
| 7b | 3 | -57.26 | 10 | 0.59 | 0.71 | 0.65 | 13 |
| 7g | 2 | -50.80 | 10 | 0.52 | 0.71 | 0.62 | 14 |
| 7s | 6 | -50.58 | 10 | 0.52 | 0.71 | 0.62 | 15 |
| ropivacaineR | 6 | -49.29 | 10 | 0.51 | 0.71 | 0.61 | 16 |
| 7aa | 7 | -48.08 | 10 | 0.50 | 0.71 | 0.61 | 17 |
| lidocaine | 5 | -47.46 | 10 | 0.49 | 0.71 | 0.60 | 18 |
| 7w | 6 | -46.85 | 10 | 0.48 | 0.71 | 0.60 | 19 |
| 7n | 3 | -46.65 | 10 | 0.48 | 0.71 | 0.60 | 20 |
| 7i | 2 | -46.57 | 10 | 0.48 | 0.71 | 0.60 | 21 |
| 6y | 9 | -44.51 | 10 | 0.46 | 0.71 | 0.59 | 22 |
| 7ad | 7 | -40.63 | 10 | 0.42 | 0.71 | 0.57 | 23 |
| 6a | 4 | -36.80 | 10 | 0.38 | 0.71 | 0.55 | 24 |
| 6ac | 2 | -35.34 | 10 | 0.37 | 0.71 | 0.54 | 25 |
| 6m | 8 | -32.97 | 10 | 0.34 | 0.71 | 0.53 | 26 |
| 6j | 7 | -30.67 | 10 | 0.32 | 0.71 | 0.52 | 27 |
| 7r | 4 | -30.01 | 10 | 0.31 | 0.71 | 0.51 | 28 |
| 6c | 8 | -24.17 | 10 | 0.26 | 0.71 | 0.48 | 29 |
| 6p | 7 | -21.75 | 10 | 0.23 | 0.71 | 0.47 | 30 |
| 6b | 1 | -75.05 | 9 | 0.77 | 0.57 | 0.67 | 31 |
| 7y | 0 | -55.02 | 9 | 0.57 | 0.57 | 0.57 | 32 |
| 7ac | 8 | -54.26 | 9 | 0.56 | 0.57 | 0.57 | 33 |
| 6q | 5 | -53.00 | 9 | 0.55 | 0.57 | 0.56 | 34 |
| 6s | 2 | -52.25 | 9 | 0.54 | 0.57 | 0.56 | 35 |
| 7p | 0 | -51.05 | 9 | 0.53 | 0.57 | 0.55 | 36 |
| 6u | 7 | -50.99 | 9 | 0.53 | 0.57 | 0.55 | 37 |
| 6o | 5 | -50.58 | 9 | 0.52 | 0.57 | 0.55 | 38 |
| 6r | 6 | -48.09 | 9 | 0.50 | 0.57 | 0.53 | 39 |
| 6g | 2 | -45.78 | 9 | 0.47 | 0.57 | 0.52 | 40 |
| 6e | 6 | -43.07 | 9 | 0.45 | 0.57 | 0.51 | 41 |
| 6aa | 5 | -41.59 | 9 | 0.43 | 0.57 | 0.50 | 42 |
| 7k | 6 | -40.86 | 9 | 0.42 | 0.57 | 0.50 | 43 |
| 7q | 4 | -39.27 | 9 | 0.41 | 0.57 | 0.49 | 44 |
| 6l | 7 | -38.61 | 9 | 0.40 | 0.57 | 0.49 | 45 |
| 7o | 9 | -31.95 | 9 | 0.33 | 0.57 | 0.45 | 46 |
| 7j | 0 | -29.65 | 9 | 0.31 | 0.57 | 0.44 | 47 |
| 6w | 4 | -24.23 | 9 | 0.26 | 0.57 | 0.41 | 48 |
| 7ab | 0 | -64.94 | 8 | 0.67 | 0.43 | 0.55 | 49 |
| 7z | 3 | -61.51 | 8 | 0.63 | 0.43 | 0.53 | 50 |
| 6n | 0 | -58.22 | 8 | 0.60 | 0.43 | 0.51 | 51 |
| 7v | 1 | -56.59 | 8 | 0.58 | 0.43 | 0.51 | 52 |
| 7l | 1 | -56.37 | 8 | 0.58 | 0.43 | 0.50 | 53 |
| 7t | 0 | -56.26 | 8 | 0.58 | 0.43 | 0.50 | 54 |
| 6t | 2 | -53.58 | 8 | 0.55 | 0.43 | 0.49 | 55 |
| 6ab | 2 | -52.49 | 8 | 0.54 | 0.43 | 0.48 | 56 |
| 6h | 5 | -52.00 | 8 | 0.54 | 0.43 | 0.48 | 57 |
| 7e | 4 | -50.38 | 8 | 0.52 | 0.43 | 0.47 | 58 |
| 7u | 2 | -47.43 | 8 | 0.49 | 0.43 | 0.46 | 59 |
| 6k | 5 | -37.53 | 8 | 0.39 | 0.43 | 0.41 | 60 |
| 7m | 3 | -32.14 | 8 | 0.34 | 0.43 | 0.38 | 61 |
| 6i | 1 | -64.25 | 7 | 0.66 | 0.29 | 0.47 | 62 |
| 6v | 4 | -54.64 | 7 | 0.56 | 0.29 | 0.42 | 63 |
| 6ad | 0 | -54.54 | 7 | 0.56 | 0.29 | 0.42 | 64 |
| 7f | 2 | -37.33 | 7 | 0.39 | 0.29 | 0.34 | 65 |

**Table S2. Ranking of poses of the 65 compounds in Kv1.5 – side-pockets. INDEX:** Corresponds to the pose number of each compound within the 10 poses generated in the IFD protocol. **DGBIN:** corresponds to the binding free energy value calculated through MM-GBSA. **INT:** is the number of site residues reported for the LA in the channel with which the ligand is interacting at 5 Å. **DGBIND_NORM:** corresponds to the normalization value of the binding free energy (at more favorable energy values it will tend to 1). **INT_NORM**: corresponds to the normalization value with respect to the number of interactions with the residues of the binding site (the greater the number of interactions it will tend to 1). **NORMT:** Sum of the normalization values divided by two. **RANK:** Position in the ranking of the compound according to the normalization value and number of interactions.

| **NAME** | **INDEX** | **DGBIND** | **INT** | **DGBIND_NORM** | **INT_NORM** | **NORMT** | **RANK** |
| --- | --- | --- | --- | --- | --- | --- | --- |
| 6a | 2 | -82.20 | 6 | 0.71 | 1.00 | 0.85 | 1 |
| 6e | 0 | -81.28 | 6 | 0.69 | 1.00 | 0.85 | 2 |
| 7c | 0 | -96.88 | 5 | 0.91 | 0.80 | 0.85 | 3 |
| 7d | 5 | -94.35 | 5 | 0.87 | 0.80 | 0.84 | 4 |
| 6d | 3 | -93.14 | 5 | 0.86 | 0.80 | 0.83 | 5 |
| 7b | 0 | -91.13 | 5 | 0.83 | 0.80 | 0.81 | 6 |
| 6c | 5 | -90.26 | 5 | 0.82 | 0.80 | 0.81 | 7 |
| 6b | 2 | -90.19 | 5 | 0.82 | 0.80 | 0.81 | 8 |
| 7a | 2 | -84.73 | 5 | 0.74 | 0.80 | 0.77 | 9 |
| 6f | 2 | -83.17 | 5 | 0.72 | 0.80 | 0.76 | 10 |
| 6y | 0 | -80.25 | 5 | 0.68 | 0.80 | 0.74 | 11 |
| ropivacaineS | 0 | -75.65 | 5 | 0.62 | 0.80 | 0.71 | 12 |
| 7j | 1 | -75.03 | 5 | 0.61 | 0.80 | 0.70 | 13 |
| 6s | 1 | -74.92 | 5 | 0.61 | 0.80 | 0.70 | 14 |
| 6z | 2 | -74.89 | 5 | 0.61 | 0.80 | 0.70 | 15 |
| 6o | 1 | -74.61 | 5 | 0.60 | 0.80 | 0.70 | 16 |
| 7t | 2 | -74.42 | 5 | 0.60 | 0.80 | 0.70 | 17 |
| bupivacaineR | 3 | -74.19 | 5 | 0.60 | 0.80 | 0.70 | 18 |
| 6p | 1 | -73.96 | 5 | 0.59 | 0.80 | 0.70 | 19 |
| bupivacaineS | 2 | -73.93 | 5 | 0.59 | 0.80 | 0.70 | 20 |
| 6t | 0 | -73.20 | 5 | 0.58 | 0.80 | 0.69 | 21 |
| 6v | 1 | -73.03 | 5 | 0.58 | 0.80 | 0.69 | 22 |
| 7h | 2 | -72.29 | 5 | 0.57 | 0.80 | 0.69 | 23 |
| 7ab | 4 | -71.47 | 5 | 0.56 | 0.80 | 0.68 | 24 |
| 6h | 1 | -70.83 | 5 | 0.55 | 0.80 | 0.68 | 25 |
| 6l | 2 | -70.80 | 5 | 0.55 | 0.80 | 0.68 | 26 |
| 7e | 0 | -70.74 | 5 | 0.55 | 0.80 | 0.68 | 27 |
| 6r | 1 | -70.67 | 5 | 0.55 | 0.80 | 0.67 | 28 |
| 6ad | 3 | -70.51 | 5 | 0.55 | 0.80 | 0.67 | 29 |
| 7z | 2 | -70.13 | 5 | 0.54 | 0.80 | 0.67 | 30 |
| 6u | 2 | -69.56 | 5 | 0.53 | 0.80 | 0.67 | 31 |
| 7k | 0 | -69.41 | 5 | 0.53 | 0.80 | 0.67 | 32 |
| 7m | 4 | -69.15 | 5 | 0.53 | 0.80 | 0.66 | 33 |
| 6x | 5 | -69.12 | 5 | 0.53 | 0.80 | 0.66 | 34 |
| 7i | 1 | -69.03 | 5 | 0.53 | 0.80 | 0.66 | 35 |
| 7v | 1 | -68.72 | 5 | 0.52 | 0.80 | 0.66 | 36 |
| 7p | 0 | -67.17 | 5 | 0.50 | 0.80 | 0.65 | 37 |
| 7aa | 3 | -66.94 | 5 | 0.50 | 0.80 | 0.65 | 38 |
| 7w | 0 | -66.78 | 5 | 0.50 | 0.80 | 0.65 | 39 |
| 6i | 1 | -65.59 | 5 | 0.48 | 0.80 | 0.64 | 40 |
| 7ac | 1 | -65.34 | 5 | 0.48 | 0.80 | 0.64 | 41 |
| 6aa | 1 | -65.23 | 5 | 0.48 | 0.80 | 0.64 | 42 |
| 7ad | 2 | -64.95 | 5 | 0.47 | 0.80 | 0.64 | 43 |
| 7g | 2 | -64.56 | 5 | 0.47 | 0.80 | 0.63 | 44 |
| 6n | 5 | -64.05 | 5 | 0.46 | 0.80 | 0.63 | 45 |
| 6m | 6 | -63.12 | 5 | 0.45 | 0.80 | 0.62 | 46 |
| 7f | 7 | -62.90 | 5 | 0.44 | 0.80 | 0.62 | 47 |
| 7n | 2 | -62.74 | 5 | 0.44 | 0.80 | 0.62 | 48 |
| 7r | 1 | -62.07 | 5 | 0.43 | 0.80 | 0.62 | 49 |
| 6ac | 2 | -61.47 | 5 | 0.42 | 0.80 | 0.61 | 50 |
| lidocaine | 4 | -60.68 | 5 | 0.41 | 0.80 | 0.61 | 51 |
| 7s | 7 | -60.40 | 5 | 0.41 | 0.80 | 0.60 | 52 |
| 7q | 0 | -60.38 | 5 | 0.41 | 0.80 | 0.60 | 53 |
| 6j | 1 | -58.85 | 5 | 0.39 | 0.80 | 0.59 | 54 |
| 6ab | 6 | -52.40 | 5 | 0.30 | 0.80 | 0.55 | 55 |
| 7u | 1 | -79.26 | 4 | 0.67 | 0.60 | 0.63 | 56 |
| 7x | 0 | -77.54 | 4 | 0.64 | 0.60 | 0.62 | 57 |
| 6q | 0 | -74.30 | 4 | 0.60 | 0.60 | 0.60 | 58 |
| 7l | 0 | -73.79 | 4 | 0.59 | 0.60 | 0.60 | 59 |
| 6g | 1 | -72.67 | 4 | 0.58 | 0.60 | 0.59 | 60 |
| ropivacaineR | 4 | -72.63 | 4 | 0.58 | 0.60 | 0.59 | 61 |
| 7y | 6 | -71.86 | 4 | 0.57 | 0.60 | 0.58 | 62 |
| 6k | 1 | -62.51 | 4 | 0.44 | 0.60 | 0.52 | 63 |
| 6w | 0 | -61.06 | 4 | 0.42 | 0.60 | 0.51 | 64 |
| 7o | 5 | -58.82 | 4 | 0.39 | 0.60 | 0.49 | 65 |

**Table S3. Ranking of poses of the 65 compounds in TASK-1. INDEX:** Corresponds to the pose number of each compound within the 10 poses generated in the IFD protocol. **DGBIN:** corresponds to the binding free energy value calculated through MM-GBSA. **INT:** is the number of site residues reported for the LA in the channel with which the ligand is interacting at 5 Å. **DGBIND_NORM:** corresponds to the normalization value of the binding free energy (at more favorable energy values it will tend to 1). **INT_NORM**: corresponds to the normalization value with respect to the number of interactions with the residues of the binding site (the greater the number of interactions it will tend to 1). **NORMT:** Sum of the normalization values divided by two. **RANK:** Position in the ranking of the compound according to the normalization value and number of interactions.

| **NAME** | **INDEX** | **DGBIND** | **INT** | **DGBIND_NORM** | **INT_NORM** | **NORMT** | **RANK** |
| --- | --- | --- | --- | --- | --- | --- | --- |
| 6d | 0 | -91.10 | 13 | 0.75 | 1.00 | 0.88 | 1 |
| 6c | 0 | -90.84 | 13 | 0.75 | 1.00 | 0.88 | 2 |
| 7b | 5 | -87.68 | 13 | 0.71 | 1.00 | 0.85 | 3 |
| 6p | 0 | -87.67 | 13 | 0.71 | 1.00 | 0.85 | 4 |
| 7a | 3 | -82.66 | 13 | 0.64 | 1.00 | 0.82 | 5 |
| 7m | 0 | -81.89 | 13 | 0.63 | 1.00 | 0.81 | 6 |
| 6a | 5 | -76.95 | 13 | 0.56 | 1.00 | 0.78 | 7 |
| 7ac | 0 | -75.86 | 13 | 0.55 | 1.00 | 0.77 | 8 |
| 7aa | 4 | -75.76 | 13 | 0.55 | 1.00 | 0.77 | 9 |
| 6f | 2 | -71.19 | 13 | 0.48 | 1.00 | 0.74 | 10 |
| 7ab | 5 | -61.22 | 13 | 0.35 | 1.00 | 0.67 | 11 |
| 6n | 9 | -44.56 | 13 | 0.12 | 1.00 | 0.56 | 12 |
| 7c | 1 | -99.72 | 12 | 0.87 | 0.89 | 0.88 | 13 |
| 7x | 0 | -93.08 | 12 | 0.78 | 0.89 | 0.83 | 14 |
| 6e | 2 | -90.38 | 12 | 0.74 | 0.89 | 0.82 | 15 |
| 7d | 3 | -88.61 | 12 | 0.72 | 0.89 | 0.80 | 16 |
| 7v | 3 | -82.11 | 12 | 0.63 | 0.89 | 0.76 | 17 |
| 7i | 6 | -82.02 | 12 | 0.63 | 0.89 | 0.76 | 18 |
| ropivacaineR | 2 | -79.12 | 12 | 0.59 | 0.89 | 0.74 | 19 |
| 6g | 7 | -77.72 | 12 | 0.57 | 0.89 | 0.73 | 20 |
| ropivacaineS | 3 | -76.23 | 12 | 0.55 | 0.89 | 0.72 | 21 |
| 7r | 2 | -74.76 | 12 | 0.53 | 0.89 | 0.71 | 22 |
| 6ad | 0 | -72.09 | 12 | 0.50 | 0.89 | 0.69 | 23 |
| 7g | 0 | -71.57 | 12 | 0.49 | 0.89 | 0.69 | 24 |
| 6x | 1 | -68.39 | 12 | 0.45 | 0.89 | 0.67 | 25 |
| 7h | 6 | -67.76 | 12 | 0.44 | 0.89 | 0.66 | 26 |
| 7s | 4 | -67.31 | 12 | 0.43 | 0.89 | 0.66 | 27 |
| 6q | 3 | -63.39 | 12 | 0.38 | 0.89 | 0.63 | 28 |
| 7j | 8 | -62.77 | 12 | 0.37 | 0.89 | 0.63 | 29 |
| 6o | 6 | -52.64 | 12 | 0.23 | 0.89 | 0.56 | 30 |
| 6b | 5 | -101.89 | 11 | 0.90 | 0.78 | 0.84 | 31 |
| 7w | 7 | -89.65 | 11 | 0.73 | 0.78 | 0.76 | 32 |
| bupivacaineR | 2 | -89.04 | 11 | 0.73 | 0.78 | 0.75 | 33 |
| 7l | 3 | -87.54 | 11 | 0.71 | 0.78 | 0.74 | 34 |
| 7y | 2 | -82.08 | 11 | 0.63 | 0.78 | 0.70 | 35 |
| 7n | 3 | -81.56 | 11 | 0.62 | 0.78 | 0.70 | 36 |
| 6v | 1 | -81.00 | 11 | 0.62 | 0.78 | 0.70 | 37 |
| 6h | 0 | -78.97 | 11 | 0.59 | 0.78 | 0.68 | 38 |
| 6y | 1 | -78.40 | 11 | 0.58 | 0.78 | 0.68 | 39 |
| 7k | 2 | -78.29 | 11 | 0.58 | 0.78 | 0.68 | 40 |
| 7t | 0 | -77.53 | 11 | 0.57 | 0.78 | 0.67 | 41 |
| 7p | 5 | -76.60 | 11 | 0.56 | 0.78 | 0.67 | 42 |
| 7z | 0 | -76.33 | 11 | 0.55 | 0.78 | 0.67 | 43 |
| 6s | 4 | -75.36 | 11 | 0.54 | 0.78 | 0.66 | 44 |
| 7ad | 0 | -74.37 | 11 | 0.53 | 0.78 | 0.65 | 45 |
| 7u | 7 | -72.85 | 11 | 0.51 | 0.78 | 0.64 | 46 |
| 6w | 4 | -72.84 | 11 | 0.51 | 0.78 | 0.64 | 47 |
| 6u | 1 | -72.12 | 11 | 0.50 | 0.78 | 0.64 | 48 |
| 7e | 6 | -70.89 | 11 | 0.48 | 0.78 | 0.63 | 49 |
| 6l | 3 | -68.93 | 11 | 0.45 | 0.78 | 0.62 | 50 |
| 6ab | 3 | -68.83 | 11 | 0.45 | 0.78 | 0.61 | 51 |
| 6j | 2 | -68.33 | 11 | 0.45 | 0.78 | 0.61 | 52 |
| 6i | 6 | -68.02 | 11 | 0.44 | 0.78 | 0.61 | 53 |
| 6ac | 1 | -67.92 | 11 | 0.44 | 0.78 | 0.61 | 54 |
| 7q | 6 | -66.11 | 11 | 0.42 | 0.78 | 0.60 | 55 |
| 7f | 2 | -65.84 | 11 | 0.41 | 0.78 | 0.59 | 56 |
| lidocaine | 6 | -64.83 | 11 | 0.40 | 0.78 | 0.59 | 57 |
| 6k | 2 | -64.55 | 11 | 0.39 | 0.78 | 0.59 | 58 |
| 6aa | 7 | -60.74 | 11 | 0.34 | 0.78 | 0.56 | 59 |
| 7o | 9 | -60.38 | 11 | 0.34 | 0.78 | 0.56 | 60 |
| 6z | 7 | -59.30 | 11 | 0.32 | 0.78 | 0.55 | 61 |
| bupivacaineS | 0 | -96.52 | 10 | 0.83 | 0.67 | 0.75 | 62 |
| 6t | 1 | -74.73 | 10 | 0.53 | 0.67 | 0.60 | 63 |
| 6r | 7 | -67.55 | 10 | 0.43 | 0.67 | 0.55 | 64 |
| 6m | 2 | -57.35 | 10 | 0.30 | 0.67 | 0.48 | 65 |

**Table S4. Ranking of poses of the 65 compounds in Nav1.5. INDEX:** Corresponds to the pose number of each compound within the 10 poses generated in the IFD protocol. **DGBIN:** corresponds to the binding free energy value calculated through MM-GBSA. **INT:** is the number of site residues reported for the LA in the channel with which the ligand is interacting at 5 Å. **DGBIND_NORM:** corresponds to the normalization value of the binding free energy (at more favorable energy values it will tend to 1). **INT_NORM**: corresponds to the normalization value with respect to the number of interactions with the residues of the binding site (the greater the number of interactions it will tend to 1). **NORMT:** Sum of the normalization values divided by two. **RANK:** Position in the ranking of the compound according to the normalization value and number of interactions.

| **NAME** | **INDEX** | **DGBIND** | **INT** | **DGBIND_NORM** | **INT_NORM** | **NORMT** | **RANK** |
| --- | --- | --- | --- | --- | --- | --- | --- |
| 6f | 4 | -102.24 | 4 | 1.00 | 1.00 | 1.00 | 1 |
| 6a | 1 | -92.92 | 4 | 0.85 | 1.00 | 0.93 | 2 |
| 6d | 0 | -92.75 | 4 | 0.85 | 1.00 | 0.93 | 3 |
| 6c | 0 | -89.60 | 4 | 0.80 | 1.00 | 0.90 | 4 |
| 7d | 2 | -89.04 | 4 | 0.79 | 1.00 | 0.90 | 5 |
| bupivacaineS | 0 | -87.89 | 4 | 0.77 | 1.00 | 0.89 | 6 |
| 6e | 1 | -87.69 | 4 | 0.77 | 1.00 | 0.89 | 7 |
| 7c | 3 | -86.31 | 4 | 0.75 | 1.00 | 0.87 | 8 |
| bupivacaineR | 0 | -86.08 | 4 | 0.75 | 1.00 | 0.87 | 9 |
| 6h | 0 | -84.00 | 4 | 0.71 | 1.00 | 0.86 | 10 |
| 6b | 2 | -82.51 | 4 | 0.69 | 1.00 | 0.84 | 11 |
| 6ad | 1 | -82.21 | 4 | 0.68 | 1.00 | 0.84 | 12 |
| 7t | 1 | -82.20 | 4 | 0.68 | 1.00 | 0.84 | 13 |
| 7a | 3 | -82.16 | 4 | 0.68 | 1.00 | 0.84 | 14 |
| ropivacaineR | 0 | -79.65 | 4 | 0.64 | 1.00 | 0.82 | 15 |
| ropivacaineS | 1 | -79.54 | 4 | 0.64 | 1.00 | 0.82 | 16 |
| 7w | 0 | -77.61 | 4 | 0.61 | 1.00 | 0.81 | 17 |
| 7m | 5 | -77.17 | 4 | 0.61 | 1.00 | 0.80 | 18 |
| 6z | 2 | -76.59 | 4 | 0.60 | 1.00 | 0.80 | 19 |
| 7j | 2 | -76.40 | 4 | 0.59 | 1.00 | 0.80 | 20 |
| 7z | 1 | -76.29 | 4 | 0.59 | 1.00 | 0.80 | 21 |
| 7x | 0 | -76.26 | 4 | 0.59 | 1.00 | 0.80 | 22 |
| 6u | 2 | -75.49 | 4 | 0.58 | 1.00 | 0.79 | 23 |
| 7l | 0 | -75.34 | 4 | 0.58 | 1.00 | 0.79 | 24 |
| 7i | 0 | -75.01 | 4 | 0.57 | 1.00 | 0.79 | 25 |
| 6x | 2 | -74.66 | 4 | 0.57 | 1.00 | 0.78 | 26 |
| 6v | 0 | -74.28 | 4 | 0.56 | 1.00 | 0.78 | 27 |
| 7b | 4 | -73.99 | 4 | 0.56 | 1.00 | 0.78 | 28 |
| 7y | 2 | -73.89 | 4 | 0.55 | 1.00 | 0.78 | 29 |
| 7u | 2 | -73.75 | 4 | 0.55 | 1.00 | 0.78 | 30 |
| 6p | 0 | -72.83 | 4 | 0.54 | 1.00 | 0.77 | 31 |
| 6t | 0 | -72.78 | 4 | 0.54 | 1.00 | 0.77 | 32 |
| 6s | 1 | -72.61 | 4 | 0.53 | 1.00 | 0.77 | 33 |
| 7ab | 0 | -72.35 | 4 | 0.53 | 1.00 | 0.76 | 34 |
| 6ac | 2 | -72.29 | 4 | 0.53 | 1.00 | 0.76 | 35 |
| 7e | 2 | -72.24 | 4 | 0.53 | 1.00 | 0.76 | 36 |
| 7v | 4 | -71.20 | 4 | 0.51 | 1.00 | 0.76 | 37 |
| 7o | 0 | -71.18 | 4 | 0.51 | 1.00 | 0.76 | 38 |
| 6l | 4 | -70.67 | 4 | 0.50 | 1.00 | 0.75 | 39 |
| 7aa | 3 | -70.65 | 4 | 0.50 | 1.00 | 0.75 | 40 |
| 6o | 1 | -70.52 | 4 | 0.50 | 1.00 | 0.75 | 41 |
| 6n | 1 | -70.06 | 4 | 0.49 | 1.00 | 0.75 | 42 |
| 7s | 2 | -69.92 | 4 | 0.49 | 1.00 | 0.75 | 43 |
| 6q | 4 | -68.97 | 4 | 0.48 | 1.00 | 0.74 | 44 |
| 7k | 1 | -68.80 | 4 | 0.47 | 1.00 | 0.74 | 45 |
| 6i | 1 | -68.67 | 4 | 0.47 | 1.00 | 0.74 | 46 |
| 6w | 0 | -68.62 | 4 | 0.47 | 1.00 | 0.74 | 47 |
| 7h | 0 | -68.50 | 4 | 0.47 | 1.00 | 0.73 | 48 |
| 6y | 2 | -68.31 | 4 | 0.47 | 1.00 | 0.73 | 49 |
| 7n | 3 | -68.15 | 4 | 0.46 | 1.00 | 0.73 | 50 |
| 7q | 0 | -67.84 | 4 | 0.46 | 1.00 | 0.73 | 51 |
| lidocaine | 1 | -67.59 | 4 | 0.45 | 1.00 | 0.73 | 52 |
| 7f | 2 | -67.55 | 4 | 0.45 | 1.00 | 0.73 | 53 |
| 7ac | 0 | -67.36 | 4 | 0.45 | 1.00 | 0.73 | 54 |
| 7p | 4 | -66.24 | 4 | 0.43 | 1.00 | 0.72 | 55 |
| 6j | 6 | -66.05 | 4 | 0.43 | 1.00 | 0.72 | 56 |
| 6ab | 3 | -65.52 | 4 | 0.42 | 1.00 | 0.71 | 57 |
| 7g | 0 | -64.80 | 4 | 0.41 | 1.00 | 0.71 | 58 |
| 6r | 0 | -64.32 | 4 | 0.40 | 1.00 | 0.70 | 59 |
| 7ad | 0 | -63.79 | 4 | 0.40 | 1.00 | 0.70 | 60 |
| 6g | 1 | -62.08 | 4 | 0.37 | 1.00 | 0.68 | 61 |
| 7r | 0 | -61.90 | 4 | 0.37 | 1.00 | 0.68 | 62 |
| 6k | 0 | -59.78 | 4 | 0.33 | 1.00 | 0.67 | 63 |
| 6aa | 1 | -56.88 | 4 | 0.29 | 1.00 | 0.64 | 64 |
| 6m | 5 | -53.53 | 4 | 0.23 | 1.00 | 0.62 | 65 |

**Table S5. Ranking of the 65 compounds in the TASK1, Nav1.5 and Kv1.5 channels (central cavity and side-pockets). The top 10 compounds selected for synthesis are in bold. POSITION:** Position in the ranking. **NAME:** compound. **DGBIND:** corresponds to the binding free energy value calculated through MM-GBSA in each channel. **INDEX:** Corresponds to the pose number of each compound within the 10 poses generated in the IFD protocol. **INT:** is the number of LA binding site residues within 5 Å of the ligand. **RANK:** Position in the ranking of the compound according to the normalization value and number of interactions. **NORMT:** Sum of the normalization values of the binding free energy and the normalization respect to the number of interactions with the binding site residues in each channel. **GLOBAL RANK:** Sum of the position in the ranking of the compound in the three channels. **GLOBAL NORMT:** Sum of the normalization values in the three channels.

| **POSITION** | **NAME** | **KV_CC DGBIND** | **KV_SP DGBIND** | **NAV DGBIND** | **TASK1 DGBIND** | **KV_CC INDEX** | **KV_SP INDEX** | **NAV INDEX** | **TASK1 INDEX** | **KV_CC INT** | **KV_SP INT** | **NAV INT** | **TASK1 INT** | **KV_CC NORMT** | **KV_SP NORMT** | **NAV NORMT** | **TASK1 NORMT** | **KV_CC RANK** | **KV_SP RANK** | **NAV RANK** | **TASK1 RANK** | **GLOBAL RANK** | **GLOBAL NORMT** |
| --- | --- | --- | --- | --- | --- | --- | --- | --- | --- | --- | --- | --- | --- | --- | --- | --- | --- | --- | --- | --- | --- | --- | --- |
| 1 | **6d** | -70.49 | -93.14 | -92.75 | -91.10 | 0 | 3 | 0 | 0 | 10 | 5 | 4 | 13 | 0.72 | 0.83 | 0.93 | 0.88 | 11 | 7 | 3 | 0 | 21 | 3.35 |
| 2 | **6f** | -42.04 | -83.17 | -102.24 | -71.19 | 8 | 2 | 4 | 2 | 11 | 5 | 4 | 13 | 0.65 | 0.76 | 1.00 | 0.74 | 4 | 19 | 0 | 9 | 32 | 3.15 |
| 3 | **7d** | -76.12 | -94.35 | -89.04 | -88.61 | 3 | 5 | 2 | 3 | 10 | 5 | 4 | 12 | 0.75 | 0.84 | 0.90 | 0.80 | 10 | 6 | 9 | 20 | 45 | 3.28 |
| 4 | **6a** | -36.80 | -82.20 | -92.92 | -76.95 | 4 | 2 | 1 | 5 | 10 | 6 | 4 | 13 | 0.55 | 0.85 | 0.93 | 0.78 | 38 | 0 | 2 | 6 | 46 | 3.11 |
| 5 | **7c** | -63.55 | -96.88 | -86.31 | -99.72 | 3 | 0 | 3 | 1 | 10 | 5 | 4 | 12 | 0.68 | 0.85 | 0.87 | 0.88 | 14 | 5 | 16 | 13 | 48 | 3.29 |
| 6 | **7a** | -67.60 | -84.73 | -82.16 | -82.66 | 3 | 2 | 3 | 3 | 12 | 5 | 4 | 13 | 0.85 | 0.77 | 0.84 | 0.82 | 0 | 17 | 31 | 4 | 52 | 3.28 |
| 7 | **6c** | -24.17 | -90.26 | -89.60 | -90.84 | 8 | 5 | 0 | 0 | 10 | 5 | 4 | 13 | 0.48 | 0.81 | 0.90 | 0.88 | 50 | 10 | 7 | 1 | 68 | 3.07 |
| 8 | ropivacaineS | -41.67 | -75.65 | -79.54 | -76.23 | 7 | 0 | 1 | 3 | 11 | 5 | 4 | 12 | 0.64 | 0.71 | 0.82 | 0.72 | 5 | 34 | 43 | 29 | 111 | 2.90 |
| 9 | **7b** | -57.26 | -91.13 | -73.99 | -87.68 | 3 | 0 | 4 | 5 | 10 | 5 | 4 | 13 | 0.65 | 0.81 | 0.78 | 0.85 | 18 | 9 | 83 | 2 | 112 | 3.10 |
| 10 | bupivacaineR | -54.49 | -74.19 | -86.08 | -89.04 | 5 | 3 | 0 | 2 | 11 | 5 | 4 | 11 | 0.71 | 0.70 | 0.87 | 0.75 | 3 | 42 | 17 | 69 | 131 | 3.03 |
| 11 | **6e** | -43.07 | -81.28 | -87.69 | -90.38 | 6 | 0 | 1 | 2 | 9 | 6 | 4 | 12 | 0.51 | 0.85 | 0.89 | 0.82 | 104 | 1 | 11 | 19 | 135 | 3.06 |
| 12 | **6b** | -75.05 | -90.19 | -82.51 | -101.89 | 1 | 2 | 2 | 5 | 9 | 5 | 4 | 11 | 0.67 | 0.81 | 0.84 | 0.84 | 55 | 11 | 27 | 57 | 150 | 3.16 |
| 13 | 6p | -21.75 | -73.96 | -72.83 | -87.67 | 7 | 1 | 0 | 0 | 10 | 5 | 4 | 13 | 0.47 | 0.70 | 0.77 | 0.85 | 51 | 43 | 96 | 3 | 193 | 2.79 |
| 14 | 6x | -35.59 | -69.12 | -74.66 | -68.39 | 3 | 5 | 2 | 1 | 11 | 5 | 4 | 12 | 0.61 | 0.66 | 0.78 | 0.67 | 8 | 68 | 78 | 40 | 194 | 2.73 |
| 15 | 7i | -46.57 | -69.03 | -75.01 | -82.02 | 2 | 1 | 0 | 6 | 10 | 5 | 4 | 12 | 0.60 | 0.66 | 0.79 | 0.76 | 29 | 70 | 74 | 25 | 198 | 2.81 |
| 16 | 7w | -46.85 | -66.78 | -77.61 | -89.65 | 6 | 0 | 0 | 7 | 10 | 5 | 4 | 11 | 0.60 | 0.65 | 0.81 | 0.76 | 27 | 84 | 52 | 67 | 230 | 2.81 |
| 17 | 7aa | -48.08 | -66.94 | -70.65 | -75.76 | 7 | 3 | 3 | 4 | 10 | 5 | 4 | 13 | 0.61 | 0.65 | 0.75 | 0.77 | 23 | 82 | 128 | 8 | 241 | 2.78 |
| 18 | 7h | -40.18 | -72.29 | -68.50 | -67.76 | 3 | 2 | 0 | 6 | 11 | 5 | 4 | 12 | 0.64 | 0.69 | 0.73 | 0.66 | 6 | 50 | 164 | 41 | 261 | 2.72 |
| 19 | bupivacaineS | -62.69 | -73.93 | -87.89 | -96.52 | 0 | 2 | 0 | 0 | 11 | 5 | 4 | 10 | 0.75 | 0.70 | 0.89 | 0.75 | 2 | 44 | 10 | 215 | 271 | 3.08 |
| 20 | 7x | -37.10 | -77.54 | -76.26 | -93.08 | 2 | 0 | 0 | 0 | 11 | 4 | 4 | 12 | 0.62 | 0.62 | 0.80 | 0.83 | 7 | 184 | 66 | 14 | 271 | 2.87 |
| 21 | 7j | -29.65 | -75.03 | -76.40 | -62.77 | 0 | 1 | 2 | 8 | 9 | 5 | 4 | 12 | 0.44 | 0.70 | 0.80 | 0.63 | 125 | 35 | 63 | 49 | 272 | 2.57 |
| 22 | ropivacaineR | -49.29 | -72.63 | -79.65 | -79.12 | 6 | 4 | 0 | 2 | 10 | 4 | 4 | 12 | 0.61 | 0.59 | 0.82 | 0.74 | 22 | 208 | 42 | 26 | 298 | 2.76 |
| 23 | 6z | -32.45 | -74.89 | -76.59 | -59.30 | 8 | 2 | 2 | 7 | 11 | 5 | 4 | 11 | 0.60 | 0.70 | 0.80 | 0.55 | 9 | 37 | 61 | 196 | 303 | 2.65 |
| 24 | 7ab | -64.94 | -71.47 | -72.35 | -61.22 | 0 | 4 | 0 | 5 | 8 | 5 | 4 | 13 | 0.55 | 0.68 | 0.76 | 0.67 | 141 | 53 | 104 | 10 | 308 | 2.67 |
| 25 | 6o | -50.58 | -74.61 | -70.52 | -52.64 | 5 | 1 | 1 | 6 | 9 | 5 | 4 | 12 | 0.55 | 0.70 | 0.75 | 0.56 | 85 | 39 | 131 | 53 | 308 | 2.56 |
| 26 | 7s | -50.58 | -60.40 | -69.92 | -67.31 | 6 | 7 | 2 | 4 | 10 | 5 | 4 | 12 | 0.62 | 0.60 | 0.75 | 0.66 | 21 | 121 | 140 | 42 | 324 | 2.63 |
| 27 | 6s | -52.25 | -74.92 | -72.61 | -75.36 | 2 | 1 | 1 | 4 | 9 | 5 | 4 | 11 | 0.56 | 0.70 | 0.77 | 0.66 | 78 | 36 | 100 | 112 | 326 | 2.68 |
| 28 | 6y | -44.51 | -80.25 | -68.31 | -78.40 | 9 | 0 | 2 | 1 | 10 | 5 | 4 | 11 | 0.59 | 0.74 | 0.73 | 0.68 | 31 | 25 | 171 | 102 | 329 | 2.74 |
| 29 | 6u | -50.99 | -69.56 | -75.49 | -72.12 | 7 | 2 | 2 | 1 | 9 | 5 | 4 | 11 | 0.55 | 0.67 | 0.79 | 0.64 | 82 | 65 | 71 | 129 | 347 | 2.64 |
| 30 | 7t | -56.26 | -74.42 | -82.20 | -77.53 | 0 | 2 | 1 | 0 | 8 | 5 | 4 | 11 | 0.50 | 0.70 | 0.84 | 0.67 | 172 | 40 | 30 | 105 | 347 | 2.72 |
| 31 | 7ac | -54.26 | -65.34 | -67.36 | -75.86 | 8 | 1 | 0 | 0 | 9 | 5 | 4 | 13 | 0.57 | 0.64 | 0.73 | 0.77 | 75 | 93 | 192 | 7 | 367 | 2.70 |
| 32 | 6h | -52.00 | -70.83 | -84.00 | -78.97 | 5 | 1 | 0 | 0 | 8 | 5 | 4 | 11 | 0.48 | 0.68 | 0.86 | 0.68 | 193 | 56 | 23 | 100 | 372 | 2.70 |
| 33 | 7v | -56.59 | -68.72 | -71.20 | -82.11 | 1 | 1 | 4 | 3 | 8 | 5 | 4 | 12 | 0.51 | 0.66 | 0.76 | 0.76 | 168 | 71 | 117 | 24 | 380 | 2.68 |
| 34 | 7z | -61.51 | -70.13 | -76.29 | -76.33 | 3 | 2 | 1 | 0 | 8 | 5 | 4 | 11 | 0.53 | 0.67 | 0.80 | 0.67 | 154 | 63 | 65 | 110 | 392 | 2.66 |
| 35 | 7m | -32.14 | -69.15 | -77.17 | -81.89 | 3 | 4 | 5 | 0 | 8 | 5 | 4 | 13 | 0.38 | 0.66 | 0.80 | 0.81 | 269 | 67 | 54 | 5 | 395 | 2.66 |
| 36 | 7g | -50.80 | -64.56 | -64.80 | -71.57 | 2 | 2 | 0 | 0 | 10 | 5 | 4 | 12 | 0.62 | 0.63 | 0.71 | 0.69 | 20 | 98 | 242 | 37 | 397 | 2.65 |
| 37 | 7n | -46.65 | -62.74 | -68.15 | -81.56 | 3 | 2 | 3 | 3 | 10 | 5 | 4 | 11 | 0.60 | 0.62 | 0.73 | 0.70 | 28 | 106 | 173 | 93 | 400 | 2.65 |
| 38 | 6n | -58.22 | -64.05 | -70.06 | -44.56 | 0 | 5 | 1 | 9 | 8 | 5 | 4 | 13 | 0.51 | 0.63 | 0.75 | 0.56 | 163 | 100 | 137 | 11 | 411 | 2.45 |
| 39 | 6ac | -35.34 | -61.47 | -72.29 | -67.92 | 2 | 2 | 2 | 1 | 10 | 5 | 4 | 11 | 0.54 | 0.61 | 0.76 | 0.61 | 39 | 112 | 106 | 156 | 413 | 2.53 |
| 40 | 7k | -40.86 | -69.41 | -68.80 | -78.29 | 6 | 0 | 1 | 2 | 9 | 5 | 4 | 11 | 0.50 | 0.67 | 0.74 | 0.68 | 110 | 66 | 157 | 103 | 436 | 2.58 |
| 41 | 6l | -38.61 | -70.80 | -70.67 | -68.93 | 7 | 2 | 4 | 3 | 9 | 5 | 4 | 11 | 0.49 | 0.68 | 0.75 | 0.62 | 115 | 57 | 127 | 147 | 446 | 2.53 |
| 42 | 6ad | -54.54 | -70.51 | -82.21 | -72.09 | 0 | 3 | 1 | 0 | 7 | 5 | 4 | 12 | 0.42 | 0.67 | 0.84 | 0.69 | 334 | 61 | 29 | 36 | 460 | 2.63 |
| 43 | 7y | -55.02 | -71.86 | -73.89 | -82.08 | 0 | 6 | 2 | 2 | 9 | 4 | 4 | 11 | 0.57 | 0.58 | 0.78 | 0.70 | 74 | 216 | 85 | 91 | 466 | 2.63 |
| 44 | 6q | -53.00 | -74.30 | -68.97 | -63.39 | 5 | 0 | 4 | 3 | 9 | 4 | 4 | 12 | 0.56 | 0.60 | 0.74 | 0.63 | 77 | 198 | 154 | 48 | 477 | 2.53 |
| 45 | 7p | -51.05 | -67.17 | -66.24 | -76.60 | 0 | 0 | 4 | 5 | 9 | 5 | 4 | 11 | 0.55 | 0.65 | 0.72 | 0.67 | 81 | 79 | 212 | 109 | 481 | 2.58 |
| 46 | 7r | -30.01 | -62.07 | -61.90 | -74.76 | 4 | 1 | 0 | 2 | 10 | 5 | 4 | 12 | 0.51 | 0.62 | 0.68 | 0.71 | 48 | 110 | 291 | 33 | 482 | 2.52 |
| 47 | 7ad | -40.63 | -64.95 | -63.79 | -74.37 | 7 | 2 | 0 | 0 | 10 | 5 | 4 | 11 | 0.57 | 0.64 | 0.70 | 0.65 | 33 | 96 | 253 | 115 | 497 | 2.55 |
| 48 | lidocaine | -47.46 | -60.68 | -67.59 | -64.83 | 5 | 4 | 1 | 6 | 10 | 5 | 4 | 11 | 0.60 | 0.61 | 0.73 | 0.59 | 24 | 117 | 185 | 175 | 501 | 2.52 |
| 49 | 7e | -50.38 | -70.74 | -72.24 | -70.89 | 4 | 0 | 2 | 6 | 8 | 5 | 4 | 11 | 0.47 | 0.68 | 0.76 | 0.63 | 206 | 59 | 108 | 135 | 508 | 2.54 |
| 50 | 7l | -56.37 | -73.79 | -75.34 | -87.54 | 1 | 0 | 0 | 3 | 8 | 4 | 4 | 11 | 0.50 | 0.60 | 0.79 | 0.74 | 170 | 201 | 73 | 75 | 519 | 2.63 |
| 51 | 6j | -30.67 | -58.85 | -66.05 | -68.33 | 7 | 1 | 6 | 2 | 10 | 5 | 4 | 11 | 0.52 | 0.59 | 0.72 | 0.61 | 47 | 128 | 214 | 153 | 542 | 2.44 |
| 52 | 6v | -54.64 | -73.03 | -74.28 | -81.00 | 4 | 1 | 0 | 1 | 7 | 5 | 4 | 11 | 0.42 | 0.69 | 0.78 | 0.70 | 331 | 47 | 81 | 97 | 556 | 2.59 |
| 53 | 7q | -39.27 | -60.38 | -67.84 | -66.11 | 4 | 0 | 0 | 6 | 9 | 5 | 4 | 11 | 0.49 | 0.60 | 0.73 | 0.60 | 113 | 122 | 178 | 170 | 583 | 2.42 |
| 54 | 6t | -53.58 | -73.20 | -72.78 | -74.73 | 2 | 0 | 0 | 1 | 8 | 5 | 4 | 10 | 0.49 | 0.69 | 0.77 | 0.60 | 183 | 46 | 97 | 270 | 596 | 2.55 |
| 55 | 7u | -47.43 | -79.26 | -73.75 | -72.85 | 2 | 1 | 2 | 7 | 8 | 4 | 4 | 11 | 0.46 | 0.63 | 0.78 | 0.64 | 224 | 176 | 87 | 126 | 613 | 2.51 |
| 56 | 6g | -45.78 | -72.67 | -62.08 | -77.72 | 2 | 1 | 1 | 7 | 9 | 4 | 4 | 12 | 0.52 | 0.59 | 0.68 | 0.73 | 99 | 207 | 288 | 28 | 622 | 2.53 |
| 57 | 6i | -64.25 | -65.59 | -68.67 | -68.02 | 1 | 1 | 1 | 6 | 7 | 5 | 4 | 11 | 0.47 | 0.64 | 0.74 | 0.61 | 305 | 91 | 159 | 155 | 710 | 2.46 |
| 58 | 6ab | -52.49 | -52.40 | -65.52 | -68.83 | 2 | 6 | 3 | 3 | 8 | 5 | 4 | 11 | 0.48 | 0.55 | 0.71 | 0.61 | 189 | 147 | 227 | 149 | 712 | 2.36 |
| 59 | 6r | -48.09 | -70.67 | -64.32 | -67.55 | 6 | 1 | 0 | 7 | 9 | 5 | 4 | 10 | 0.53 | 0.67 | 0.70 | 0.55 | 89 | 60 | 248 | 319 | 716 | 2.46 |
| 60 | 6w | -24.23 | -61.06 | -68.62 | -72.84 | 4 | 0 | 0 | 4 | 9 | 4 | 4 | 11 | 0.41 | 0.51 | 0.74 | 0.64 | 128 | 331 | 161 | 127 | 747 | 2.30 |
| 61 | 6aa | -41.59 | -65.23 | -56.88 | -60.74 | 5 | 1 | 1 | 7 | 9 | 5 | 4 | 11 | 0.50 | 0.64 | 0.64 | 0.56 | 109 | 95 | 379 | 190 | 773 | 2.34 |
| 62 | 7o | -31.95 | -58.82 | -71.18 | -60.38 | 9 | 5 | 0 | 9 | 9 | 4 | 4 | 11 | 0.45 | 0.49 | 0.76 | 0.56 | 123 | 365 | 118 | 193 | 799 | 2.26 |
| 63 | 7f | -37.33 | -62.90 | -67.55 | -65.84 | 2 | 7 | 2 | 2 | 7 | 5 | 4 | 11 | 0.34 | 0.62 | 0.73 | 0.59 | 414 | 105 | 186 | 171 | 876 | 2.28 |
| 64 | 6m | -32.97 | -63.12 | -53.53 | -57.35 | 8 | 6 | 5 | 2 | 10 | 5 | 4 | 10 | 0.53 | 0.62 | 0.62 | 0.48 | 43 | 103 | 420 | 350 | 916 | 2.25 |
| 65 | 6k | -37.53 | -62.51 | -59.78 | -64.55 | 5 | 1 | 0 | 2 | 8 | 4 | 4 | 11 | 0.41 | 0.52 | 0.67 | 0.59 | 260 | 311 | 320 | 177 | 1068 | 2.18 |
|  |  |  |  |  |  |  |  |  |  |  |  |  |  |  |  |  |  |  |  |  |  |  |  |

**Table S6. Statistical analysis of GLM models and Welch´s ANOVA test for figures 4, 5, 7, and S1. Only statistically significant differences are specified.**

| Figure | Comparison | Model type | Type II ANOVA test | Estimate ± SE | z-value | p-value | Significance level (code) |
| --- | --- | --- | --- | --- | --- | --- | --- |
| Figure 4 | TASK-1 – K_v_1.5 | GLM-Gamma | χ²=128.040, df=1, p=<2.2e-16 | 51.071 ± 5.265 | 9.700 | <2e-16 | *** |
|  | 6f – 6d |  | χ²=15.897, df=9, p=0.069 | 39.525 ± 12.299 | 3.214 | 0.042 | * |
| Figure 5 | Na_v_1.5 – K_v_1.5 |  | χ²=51.883, df=2, p=5.418e-12 | 23.474 ± 4.716 | 4.977 | < 0.001 | *** |
|  | TASK-1 – K_v_1.5 |  |  | 42.471 ± 6.071 | 6.996 | < 0.001 | *** |
|  | TASK-1 – Na_v_1.5 |  |  | 18.997 ± 6.426 | 2.956 | 0.008 | ** |
|  | 6f – 7a |  | χ²=15.581, df=4, p=0.004 | -25.468 ± 7.903 | -3.222 | 0.011 | * |
|  |  |  | Welch´s ANOVA |  |  |  |  |
| Figure 7 | TASK-1 – K_v_1.5 | N/A | F_5, 12.501_ = 170.875, p=4.71e-11 | -28.334 ± 4.137 | N/A | 0.035 | * |
|  | TASK-1 – Kir2.1 |  |  | -85.099 ± 2.910 |  | < 0.001 | *** |
|  | TASK-1 – TREK-1 |  |  | -92.806 ± 7.070 |  | < 0.001 | *** |
|  | TASK-1 – TASK-4 |  |  | -124.106 ± 2.957 |  | < 0.001 | *** |
|  |  |  | Type II ANOVA test |  |  |  |  |
| Figure S1 | Kv1.5 10 µM – Kv1.5 100 µM | GLM-Gamma | χ²=14.701, df=1, p=<1.3e-4 | 71.020 ± 26.230 | 2.707 | 0.007 | ** |
|  | Na_v_1.5 10 µM – Na_v_1.5 100 µM | GLM-Gaussian | χ²=218.440, df=1, p=<2.2e-16 | 61.241 ± 4.144 | 14.78 | <2e-16 | *** |

**Table S7. Patient Characteristics.** AADs, antiarrhythmic drugs; ACE, angiotensin-converting enzyme; AT1, angiotensin II receptor type 1; BMI, body mass index; CVD, cardiovascular disease; CABG, coronary artery bypass grafting; hsTroponin, high sensitive Troponin; LA, left atrial; LVEF, left ventricular ejection fraction; SGLT2, sodium-glucose linked transporter 2; TSH, thyrotropin. **p*<0.05 versus SR; from ANOVA followed by Tukey multiple comparisons procedure for continuous variables and from Fisher’s exact test for categorical variables.

*Demographics:*

Age, y (mean ± SEM) 66.5 ± 3.7

Female, n (%) 2 (20 %)

Height, cm (mean ± SEM) 174.3 ± 2.7

Body weight, kg (mean ± SEM) 85.3 ± 4.9

BMI, kg/m^2^ (mean ± SEM) 27.9 ± 1.2

*Rhythm Status, n (%):*

Sinus Rhythm 5 (50 %)

Atrial Fibrillation (AF) 5 (50 %)

Paroxysmal AF 2 (20 %)

Permanent/Chronic AF 3 (30 %)

*Indication for heart surgery, n (%)*

CABG 9 (90 %)

Aortic valve replacement 2 (20 %)

Mitral valve replacement 2 (20 %)

*Cardiovascular history, n (%)*

Coronary artery disease 9 (90 %)

Prior myocardial infarction 0

Dilated cardiomyopathy 0

Peripheral arterial disease 1 (10 %)

Prior transient ischemic attack 0

Prior stroke 0

Pulmonary artery hypertension 3 (30 %)

*Cardiovascular risk factors, n (%)*

Hypertension 9 (90 %)

Diabetes mellitus 5 (50 %)

Hyperlipidemia 5 (50 %)

Current or former smoker 3 (30 %)

Family History of CVD 2 (20 %)

|  |  |  |
| --- | --- | --- |

*P-wave -Parameters in SR ECGs:*

P-Wave Duration, ms (mean ± SEM) 110 ± 3.33

P-Wave Area, ms*mV(mean ± SEM) 8.43 ± 0.78

PWTFV1, ms*mV (mean ± SEM) 0.05 ± 0.007

|  |
| --- |

*Echocardiography:*

LVEF, % (mean ± SEM) 49.8 ± 4.25

LA diameter, mm (mean ± SEM) 39.57 ± 1.92

Aortic stenosis, n (%) I° 3 (30 %), II° 0,III° 3 (30 %)

Aortic regurgitation, n (%) 0

Mitral stenosis, n (%) I° 0, II° 2 (20 %), III° 1 (10 %)

Mitral regurgitation, n (%) I° 2 (20 %)

Tricuspid valve regurgitation, n (%) I° 3 (30 %), II° 0, III° 2 (20 %)

Pulmonic valve regurgitation, n (%) 0

*Medical History, n (%)*

History of cancer 1 (10 %)

Chronic obstructive pulmonary disease 2 (20 %)

*Laboratory Values:*

Haemglobin, g/dl (mean ± SEM) 11.46 ± 0.47

hsTroponin T, ng/dl (mean ± SEM) 229.38 ± 110.39

Creatinine, mg/dl (mean ± SEM) 1.085 ± 0.22

TSH, mIU/l (mean ± SEM) 1.751 ± 0.25

*Concomitant medication, n (%)*

ACE-inhibitors 4 (40 %)

AT1-antagonists 4 (40 %)

Valsartan + sacubitril 0

β-blockers 9 (90 %)

SGLT2-inihibitors 2 (20 %)

Statins 8 (80 %)

Class I AADs 0

Class III AADs 0

Class IV AADs 0

Digitalis 1 (10 %)

Diuretics 6 (60 %)

Oral anticoagulation 6 (60 %)

Platelet Inhibitor 4 (40 %)

Insulin 2 (20 %)
